# Modelling the potential impact of global hepatitis B vaccination on the burden of chronic hepatitis B in the United States

**DOI:** 10.1111/jvh.13982

**Published:** 2024-07-22

**Authors:** David W. Hutton, Mehlika Toy, Danwei Yang, Hanwen Zhang, Senad Handanagic, Paige A. Armstrong, Annemarie Wasley, Nicolas A. Menzies, Hang Pham, Joshua A. Salomon, Samuel K. So

**Affiliations:** 1 Department of Health Management and Policy, University of Michigan, Ann Arbor, Michigan, USA; 2 Asian Liver Center, Department of Surgery, Stanford University School of Medicine, Stanford, California, USA; 3 Division of Viral Hepatitis, Centers for Disease Control and Prevention, Atlanta, Georgia, USA; 4 Global Immunizations Division, Centers for Disease Control and Prevention, Atlanta, Georgia, USA; 5 Department of Global Health and Population, Harvard T.H. Chan School of Public Health, Boston, Massachusetts, USA; 6 Department of Health Policy, Stanford University School of Medicine, Stanford, California, USA; 7 Center for Health Policy, Freeman Spogli Institute for International Studies, Stanford University, Stanford, California, USA

**Keywords:** economics, hepatitis B, vaccination, World Health Organization

## Abstract

About 80% of persons with chronic hepatitis B virus (HBV) infection in the United States are non-US-born. Despite improvements in infant hepatitis B vaccination globally since 2000, work remains to attain the World Health Organization’s (WHO) global 2030 goal of 90% vaccination. We explore the impacts on the United States of global progress in hepatitis B vaccination since 2000 and of achieving WHO hepatitis B vaccination goals. We simulated immigrants with HBV infection arriving to the United States from 2000 to 2070 using models of the 10 countries from which the largest numbers of individuals with HBV infection were born. We estimated costs in the United States among these cohorts using a disease simulation model. We simulated three scenarios: a scenario with no progress in infant vaccination for hepatitis B since 2000 (baseline), current (2020) progress and achieving WHO 2030 goals for hepatitis B vaccination. We estimate current hepatitis B vaccination progress since the 2000 baseline in these 10 countries will lead to 468,686 fewer HBV infections, avoid 35,582 hepatitis B-related deaths and save $4.2 billion in the United States through 2070. Achieving the WHO 2030 90% hepatitis B infant vaccination targets could lead to an additional 16,762 fewer HBV infections, 989 fewer hepatitis B-related deaths and save $143 million through 2070. Global hepatitis B vaccination since 2000 reduced prevalence of HBV infection in the United States. Achieving the WHO 2030 infant vaccination goals globally could lead to over one hundred million dollars in additional savings.

## 1 | INTRODUCTION

Hepatitis B virus (HBV) infection is a leading cause of liver-related death and can kill 15%–25% of those chronically infected.^1^ Many with chronic hepatitis B in the United States are non-US-Born^2–5^ One study suggested 1.3 million persons with chronic hepatitis B infection came to the United States from 1974 through 2008.^5^ An updated systematic review and meta-analysis estimated that 1.47 million non-US-born were living in the United States with chronic hepatitis B in 2018, that 78% of persons with chronic hepatitis B were non-US born, and that 10 countries accounted for 60% of immigrants currently in the United States with chronic hepatitis B.^3^ Another study suggested that the Philippines, China and Vietnam together accounted for 37% of the total burden of imported cases of chronic hepatitis B.^5^ Another recent study estimated there were 1.4 million non-US-born with chronic hepatitis B in the United States in 2020.^6^ These studies agree global hepatitis elimination efforts may have a substantial impact on hepatitis B in the United States. However, none have compared the current status quo to a counterfactual with no vaccination increases over time nor have they explored the potential impact improving global hepatitis B vaccination coverage could have on the United States.

Hepatitis B infant vaccination is very effective at reducing chronic infections.^7^ Vaccination programmes are highly cost-effective strategies decreasing liver-related disease burden and costs.^8–11^ Vaccination is particularly valuable at birth and early childhood since the chance of chronic hepatitis B virus infection decreases with age.^12^ In 2016, the World Health Assembly adopted the Global Health Sector Strategy (GHSS) on viral hepatitis and called for the elimination of hepatitis B and hepatitis C as a public health problem by 2030.^13^ The WHO recommends one dose of hepatitis B vaccine at birth followed by two additional doses.^14^ The programmatic vaccination targets for 2030 hepatitis B elimination are 90% of newborns receiving timely birth-dose (first-dose within 24 h of birth) and 90% three-dose completion.

There has been substantial progress in infant hepatitis B vaccination coverage globally since 2000.^15^ By 2020, global infant three-dose hepatitis B vaccine coverage was 82% and 43% of infants were given a dose of hepatitis B vaccine within 24 h of birth.^16^ A 2017 study estimated that during 2001–2020, approximately 200 million cases of hepatitis B and 11.9 million deaths were averted due to infant hepatitis B vaccination among 73 GAVI, the Vaccine Alliance-supported low- and middle-income countries.^17^ However, some countries still have low coverage, like Nigeria with 56% and Haiti with 51% three-dose coverage.^15^

Our study had two aims: (1) to describe how the global progress in hepatitis B vaccination since 2000 has impacted the United States and (2) to explore how achieving the WHO hepatitis B 2030 vaccination targets might improve health and healthcare costs in the United States. This helps clarify the value to the United States of prior and future global hepatitis B vaccination efforts.

## 2 | METHODS

**FIGURE 1:**
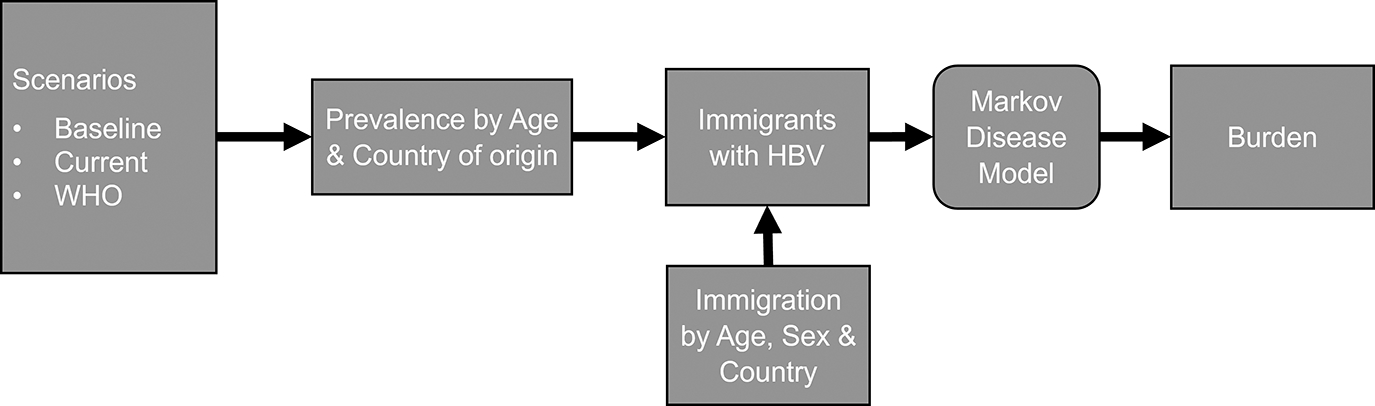
Overview of model components contributing to burden of chronic hepatitis B in immigrants to the United States. HBV, Hepatitis B; WHO, World Health Organization.

To estimate the impacts of global hepatitis B vaccination efforts on the burden of disease in the United States, we used a simulation modelling approach (Figure 1). We combined models of country-specific prevalence under different scenarios with estimates of annual numbers of immigrants from those countries to estimate numbers of immigrants by age and sex with chronic hepatitis B over the period of 2000–2070 to look about 50 years into the future since chronic hepatitis B outcomes may take decades to manifest. These estimates were incorporated into a hepatitis B Markov model to estimate health outcomes and costs (Appendix S2: Figure 1).

### 2.1 | Country-specific prevalence models

We built country-specific models to estimate historical and future chronic hepatitis B prevalence by age from a variety of countries. A systematic review and meta-analysis by Wong et al.^3^ identified China, Vietnam the Philippines, India, the Dominican Republic, Taiwan, South Korea, Mexico, Nigeria and Haiti as being the top 10 countries in numbers of immigrants in the United States with chronic hepatitis B. Nine of these countries are also estimated to be in the top 10 from another recent analysis.^6^ These countries encompass a broad array of geographies around the globe and account for 60% of immigrants in the United States with chronic hepatitis B^3^ and 51% of all immigrants.^18^ To focus our efforts, we built simulation models for each of these countries based on historical prevalence and reported hepatitis B birth-dose and three-dose vaccination coverage. Briefly, the models simulate individual birth cohorts from 1960 to 2070 and simulate chronic hepatitis B virus infection at birth, childhood and adulthood with and without vaccination evaluating infection, health and economic outcomes from 2020 through 2070. The models were based on prior hepatitis B models^19,20^ and are compared against observed historical hepatitis B prevalence by age to ensure validity. The models simulate three-dose vaccination preventing infections throughout childhood and adulthood, and birth-dose vaccination prevents mother- to-child transmission. Estimates of vaccination coverage came from WHO/UNICEF estimates.^15^ More details of the models can be found in Appendix S1.

### 2.2 | Immigration

We combined country hepatitis B prevalence estimates with country historical immigration and emigration estimates and projections of future immigration and emigration. Historical immigration and emigration estimates were based upon an analysis of American Community Survey data of the non-US-born population at different times adjusted for undercoverage based on Jensen et al.^21^ Estimates include undocumented individuals. Immigration and emigration from 2020 through 2022 were based on relative percentage changes in immigration by country since 2019 based on data from United States Homeland Security estimates during the COVID-19 pandemic.^22^ We assumed immigration after 2022 will follow United States Census Bureau 2017 national population projections of non-US-born immigration to the United States by sending region.^23^ The number of non-US-born individuals of a specific age from a specific country in the United States at any time is calculated each year by adding in immigration, subtracting emigration and subtracting expected mortality for that year. Immigration estimates and trends from each country are the same in all vaccination scenarios.

### 2.3 | Markov disease model

Starting from the estimated cohorts of individuals with chronic hepatitis B arriving in the United States from each of the 10 countries analysed, we simulated disease and cost outcomes using a Markov model of hepatitis B. At a high level, it started with individuals defined by hepatitis B disease status, age and treatment status, and simulated lifetime outcomes such as cirrhosis, liver cancer, hepatitis-B-related deaths, quality-adjusted life years (QALYs) and health system costs. Individuals could ‘exit’ the model through death or emigration, using country-specific emigration estimates. More details of the hepatitis B Markov structure and parameterization can be found in Appendix S2.

### 2.4 | Elimination scenarios and analyses

We modelled the impact of three different hepatitis B elimination scenarios.

The first was a ‘Baseline’ scenario, accounting for vaccination trends until 2000, but with no progress since that year. In that scenario, infant hepatitis B vaccination levels were frozen at 2000 levels and hepatitis B prevalence was simulated forward with those levels of vaccination.

The second scenario was the ‘Current’ scenario with actual historical levels of hepatitis B vaccination since the year 2000, with no changes in vaccination after 2020.

The final scenario was the ‘WHO’ scenario which represents achieving the WHO 2030 programmatic goals for hepatitis B vaccination. Vaccination patterns during 2000–2020 are the same as in the ‘current’ scenario. We then assumed a linear increase in vaccination from 2020 to 2030 to achieve the goal of 90% birth-dose and three-dose vaccination completion by 2030, and maintaining those levels.

In our analyses, we first compared the ‘Baseline’ scenario with the ‘Current’ scenario. We estimated the numbers of immigrants arriving to the United States each year and the impact on overall chronic hepatitis B prevalence in the United States. We also calculated hepatitis B disease-related outcomes and health system costs. We next compared the ‘Current’ scenario with the ‘WHO’ scenario. We also estimated the numbers of immigrants arriving to the United States, hepatitis B disease-related outcomes and health system costs.

### 2.5 | Scenario: Extrapolation to the rest of the world

In a scenario analysis, we also extrapolated from these ‘top ten’ countries to simulate hepatitis B burden in the rest of the world to estimate the overall impact of global vaccination and immigration on hepatitis B in the United States. We started with estimates of hepatitis B prevalence in immigrants to the United States coming from the rest of the world as compared to immigrants from the ‘top ten’ countries in 2018 as reported in Wong et al.^3^ We assumed changes in hepatitis B prevalence and burden in immigrants from the rest of the world would be proportional to changes in prevalence in these ‘top ten’ countries. This assumption was applied across all time periods both before and after 2018 and applied to all scenarios. This assumption means policy changes in hepatitis B in the ‘top ten’ would lead to proportional changes in the rest of the world. For example, if the number of individuals with chronic hepatitis B from the ‘top ten’ countries decreased by 5%, the number of individuals with chronic hepatitis B from the rest of the world also decreased by 5%.

## 3 | RESULTS

### 3.1 | Baseline

**FIGURE 2:**
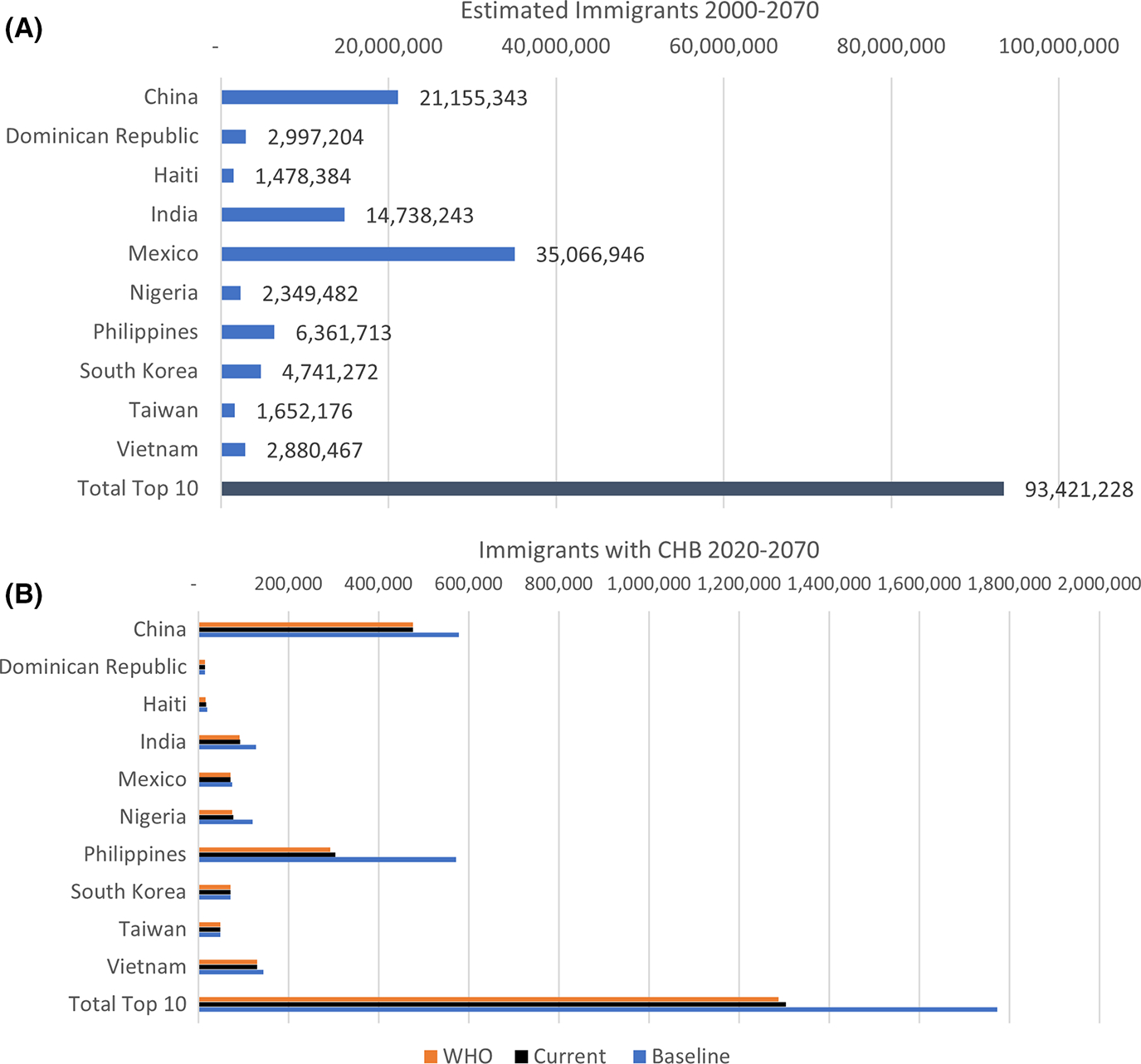
(A) Estimated Immigration from 2000 to 2070: Total Immigrants. The countries are ranked alphabetically. ‘top 10’ refers to China, Vietnam, the Philippines, India, the Dominican Republic, Taiwan, South Korea, Mexico, Nigeria and Haiti all together. (B) Estimated Immigrants with CHB under the scenarios from 2000 to 2070: ‘top 10’ refers to China, Vietnam, the Philippines, India, the Dominican Republic, Taiwan, South Korea, Mexico, Nigeria and Haiti all together. CHB, Chronic Hepatitis B. More detailed numbers for this table can be found in Appendix S2: Table 4.

Under the baseline scenario, there are an estimated 1,773,058 people arriving from these top 10 countries to the United States with chronic hepatitis B during 2000–2070, 618,429 people arriving during 2000–2019, 603,221 from 2020 to 2040 and 551,408 from 2041 to 2070 (Figure 2, Appendix S2: Figures 3–5, Appendix S2: Table 4). Out of the top 10 countries, 33% of these immigrants with chronic hepatitis B come from China, 32% from the Philippines, 8% from Vietnam and 7% each from India and Nigeria.

### 3.2 | Current versus Baseline

We estimate that increases in global infant hepatitis B vaccination coverage since 2000 will lead to 468,686 fewer individuals with chronic hepatitis B in the United States from these top 10 countries from 2000 to 2070 (Table 1, Appendix S2: Figure 6). This effect increases over time with an estimated 12,744 fewer individuals with chronic hepatitis B immigrating during 2000–2019, 127,439 fewer immigrants with chronic hepatitis B during 2020–2040 and an additional 328,503 fewer from 2041 to 2070 if vaccination levels remain at 2020 levels (Table 2, Appendix S2: Table 4, Appendix S2: Figures 4–6).

The benefits to the United States from increases in global hepatitis B vaccination varied by country due to differences in baseline epidemiology, changes in vaccination and levels of immigration. Some countries like South Korea and Taiwan already had high levels of vaccination by the year 2000. Others like Haiti, India and Nigeria had very low vaccination coverage in 2000. Immigrants from the Philippines account for 31% of the reduction in immigrants with chronic hepatitis B (Appendix S2: Figure 6a), and China accounts for 12% of the reduction. The next-most important contributors to the reduced persons with chronic hepatitis B virus infections are from Nigeria, India and Vietnam.

Achieving 2020 hepatitis B vaccination coverage and maintaining those levels until 2070 is estimated to lead to 35,582 fewer hepatitis B-related deaths from these top 10 countries by 2070 (Table 1, Appendix S2: Figure 7a) and will result in a gain of 355,017 QALYs (Table 1, Appendix S2: Figure 8a) when compared to the baseline scenario. We estimate that increases in global vaccination coverage since 2000 have led to $53 million in savings from 2000 to 2020 and will lead to over $4.2 billion in cost savings from the entire time period 2000 to 2070 (Table 1, Appendix S2: Figure 9a, Table 2). Vaccination progress from the Philippines, China, Nigeria, India and Vietnam account for $4.1 billion of those cost savings (Table 2).

### 3.3 | Value of achieving WHO goals compared to current

The WHO 2030 elimination targets involve increasing vaccination coverage to 90% of newborns receiving timely birth-dose and 90% having three-dose completion. We estimate that achieving WHO’s 2030 vaccination targets and maintaining them until 2070 will lead to an additional 16,762 fewer immigrants with chronic hepatitis B immigrating to the United States between 2020 and 2070 (Tables 1 and 2, Appendix S2: Table 4, Appendix S2: Figures 4–6b).

The impact of hepatitis B vaccination on the number of immigrants infected with HBV varies widely from county to country due to differences in immigration levels, and the differential improvements in vaccination necessary to achieve WHO 2030 vaccination targets. Some countries like Taiwan, South Korea and China have already achieved 90% vaccination coverage, while others have not yet reached the target. Of all the chronic hepatitis B virus infections that would be averted by reaching the 2030 targets compared to 2020 levels, the Philippines accounts for 37% of the total (Appendix S2: Figure 6b). In our model, Nigeria accounts for 10% of the averted infections due to increasing vaccination levels. The next-most important potential contributors to reduced hepatitis B virus infections are India, Haiti and Vietnam.

Compared to maintaining the 2020 hepatitis B vaccination coverage, achieving the WHO 2030 vaccination targets would lead to an additional 9108 QALYs gained, and 989 hepatitis B-related deaths averted compared to current coverage levels (Tables 1 and 2, Appendix S2: Figures 7b and 8b). We project that the increases in vaccination coverage are likely to lead to an additional $143 million in healthcare cost savings between 2020 and 2070 compared to maintaining 2020 vaccination coverage levels (Table 2, Appendix S2: Figure 9b). Due to immigration levels and changes in vaccination, achieving WHO hepatitis B vaccination coverage goals in the Philippines would lead to 11,333 fewer immigrants with hepatitis B, $95.1 million in savings and 636 fewer hepatitis B-related deaths in the United States (Table 2). Achieving 2030 WHO hepatitis B vaccination coverage goals in Nigeria would lead to 2959 fewer immigrants with chronic HBV, 207 fewer hepatitis B-related deaths and $27.2 million in cost savings in the United States (Table 2).

### 3.4 | Potential impacts of global hepatitis B elimination efforts

Assuming other countries would have similar relative changes in hepatitis B prevalence, if we add in individuals immigrating to the United States from other countries beyond the top 10, we estimate that under the baseline scenario 3,270,574 people with chronic hepatitis B would immigrate to the United States from 2000 to 2070, leading to 340,814 hepatitis B-related deaths in immigrants to the United States.

Increases in global infant hepatitis B vaccination coverage since 2000 are estimated to result in 864,536 fewer individuals with chronic hepatitis B in the United States, 65,635 fewer hepatitis B-related deaths and 654,862 QALYs gained and will lead to over $7.8 billion in cost savings from 2000 to 2070 (Table 2, Appendix S2: Tables 4 and 5, Appendix S2: Figures 3–9).

Achieving WHO’s 2030 vaccination targets globally will lead to an additional 30,919 fewer immigrants with chronic hepatitis B immigrating to the United States, an additional 16,800 QALYs gained, 1825 hepatitis B-related deaths averted and an additional 263.6 million in healthcare cost savings between 2020 and 2070 compared to maintaining 2020 hepatitis B vaccination coverage (Table 2, Figure 4, Appendix S2: Tables 4 and 5, Appendix S2: Figures 3–9).

Continuing efforts to maintain levels of vaccination achieved in 2020 and additional efforts to achieve WHO 2030 vaccination targets globally are anticipated to reduce the overall total number of persons with chronic hepatitis B in the United States by half compared to what they would have been under the baseline scenario by 2070 (Appendix S2: Figure 10).

## 4 | DISCUSSION

During 2018–2019, 1.6 to 2.5 million people in the United States lived with chronic hepatitis B, with 1.5 million born outside of the United States, and about 60% of that 1.5 million immigrating from 10 countries.^2,3^ We estimated progress in and maintenance of hepatitis B vaccination since 2000 in those top 10 countries leads to accelerating impact over time. Because immigrants often arrive in the United States at older ages (64% over the age of 30% and 84% over the age of 20^24^), the benefits of infant hepatitis B immunization since 2000 will grow as individuals vaccinated at the time of birth in the early 2000s begin to immigrate to the United States. If these top 10 countries maintain progress in infant hepatitis B vaccination, it will lead to 127,439 fewer immigrants arriving with chronic hepatitis B from 2020 to 2040 and an additional 328,503 fewer immigrants by 2070 and result in savings of $4.2 billion in costs. Achieving WHO 2030 vaccination targets could lead to an additional 16,762 fewer immigrants with chronic hepatitis B between 2020 and 2070 and $143 million in additional savings.

Sustained high infant hepatitis B vaccination levels consistent with WHO targets would reduce chronic hepatitis B prevalence in countries that currently have inadequate vaccination coverage like the Philippines, Nigeria, India and Haiti. Our results highlight the interconnectedness of global disease control and burden in the United States. Other countries with large immigration inflows from similar areas are also likely to see benefits.

Our findings are consistent with other literature on how immigration affects hepatitis B in the United States. Two papers estimate that the Philippines, China and Vietnam contribute about 40% of infections to the United States^5,6^ which we also find (Appendix S2: Table 4).

Other studies estimate about 50,000 individuals with chronic HBV infection immigrate each year.^5,6^ We estimate about 25,000–35,000 individuals each year from the top 10 countries and about 50,000–65,000 from all countries (Appendix S2: Table 4, Appendix S2: Figure 4b). There are likely several reasons for differences in estimates, but we included undocumented immigration while other studies only include legal permanent residents. The study projecting until 2030 estimated the total number of HBV infections in 2030 will be 1.9 million,^6^ matching our projection of 1.9 million under the current scenario (Appendix S2: Figure 10). Other models estimate about 8000 to 13,000 annual deaths from hepatitis B^5,6^ whereas we estimate 4000–5000 annual deaths from hepatitis B around 2020 under the current scenario. All models are different, but our model includes HBV treatment, which may lead to lower mortality estimates. If our model underestimates deaths, we may underestimate the benefits of further global vaccination increases on the United States. The impact of hepatitis B vaccination takes time: what we see now reflects immunization decades ago when most immigrants were born. We expect to see 10 times the benefits of reduced infection during the period 2020–2040 as compared to 2000 to 2019. The benefits of reaching the WHO 2030 vaccination targets will also take time to be seen in the United States. We anticipate increasing rates of infant vaccination to achieve WHO targets in these top 10 countries will lead to 2004 fewer immigrants with hepatitis B during the period 2020 to 2040 but will lead to 14,758 fewer immigrants with hepatitis B from 2041 to 2070. There will likely be additional benefits even beyond 2070. So, although our analysis forecasts about 50 years from the current time, our analysis may undervalue the impact of the WHO vaccination goals. Continued efforts to support global infant hepatitis B vaccination and catch-up vaccination will reduce the burden in the United States.

Different countries impact the United States based on different epidemiology, immigration levels and vaccination progress. Over 40% of the impact of current progress on reducing the number of HBV-infected immigrants arriving to the United States since 2000 are due to improved vaccination in China and the Philippines (Appendix S2: Figure 4). Progress in improving vaccination levels in the Philippines since 2000 accounts for 31% of the impacts (Appendix S2: Figure 6a). Although the Philippines does not contribute as many immigrants as some other countries, vaccination rates were very low in 2000 (under 10% 3-dose coverage), but have increased since then, but there still exists much room for improvement (about 57% 3-dose coverage in 2021). China accounts for 12% because of the increasing vaccination levels, but also because of the large numbers of immigrants. Immigrants from Vietnam, India and Nigeria together account for about 12% of the overall cost savings, and further increases in coverage in these countries would result in additional future savings. COVID-19 led to an immunization backslide.^25^ In some countries, including several outlined in this paper, immunization lags far below global targets, particularly for birth-dose vaccination, which is important for prevention of mother- to-child and early childhood transmission. For example, Haiti has about 50% three-dose coverage and does not have birth dose in its immunization schedule. Nigeria has chronic hepatitis B prevalence of >10% but only 57% three-dose coverage and 52% timely birth-dose coverage. Achieving WHO immunization targets in the Philippines, Nigeria, Haiti and India could account for most of the benefits to the United States over the next 50 years. Additional efforts to increase vaccination coverage, particularly in these countries, could have the greatest impact on the United States.

### 4.1 | Limitations

Our analysis has several limitations, mainly due to the modelling approach and underlying assumptions. Since we start with year-2000 levels of vaccination, we miss the impact in countries that were already achieving high coverage before 2000 (e.g. South Korea and Taiwan) and thus we underestimate the total impact of global hepatitis B vaccination on the United States. Countries like China, Taiwan and South Korea implemented hepatitis B immunoglobulin for infants born to HBV+ mothers, as well as antiviral treatment of pregnant women to prevent mother- to-child transmission. Although our model does not include these interventions, it includes birth-dose vaccination, which is highly effective at preventing mother- to-child transmission.^19,20^ Our model used 2020 estimates of ‘current’ vaccination coverage, but these vaccination levels were lower due to the COVID-19 pandemic.^26^ We also censor impact after 2070 which may miss benefits, particularly for progress achieved under the WHO scenario. Our analysis is based upon estimates from Wong on the number and percentage of people who are foreign-born and have chronic HBV infection in the United States. The modelling of the prevalence in the countries of origin may be inaccurate, and prevalence in immigrants may be different from general prevalence. Current United States surveillance systems do not capture the place of birth and are insufficient for tracking this information. We do not account for reduced domestic transmission of hepatitis B in the United States due to vaccination, so we may underestimate the value of vaccination. To the extent future immigration does not follow census projections of future immigration, our conclusions would change. We simulate 10 countries that constitute the top 10 contributors to the prevalence of hepatitis B in the United States in 2018. Although prevalence in 2018 is correlated with annual number of incoming immigrants, it may perfectly match with the top countries with the highest immigration with chronic hepatitis B in the future. To extrapolate to global hepatitis B elimination, we assume the contribution from the rest of the world will be proportional to that burden observed in 2018. If this burden evolves disproportionately to a different mix of countries, this may affect our conclusions. Census forecasts of future immigration are based on relative population growth around the globe and suggest there may be more immigration to the United States from Africa in the future, which may be slightly different from the mix as seen in the United States in 2018 which is more heavily weighted towards immigrants from Asia. We make projections through 2070, which is important to estimate long-term impact, but the long timeline leads to substantial uncertainty in demography, immigration patterns, epidemiology and medical advancements. This analysis incorporates WHO global hepatitis B vaccination goals, but not screening or antiviral treatment goals of >90% of people with chronic hepatitis B diagnosed and >80% of people diagnosed are treated. Improved treatment could also decrease transmission of infection and mortality. Global progress in screening, vaccinating and linking to treatment could all have tremendous benefits to the United States.^10,27^

The increase in global hepatitis B vaccination since 2000 has provided large health and economic benefits to the United States and should continue to provide benefits in the future, if vaccination levels are maintained. Achieving the WHO 2030 vaccination targets could reduce thousands of hepatitis B-related deaths and save one billion dollars in healthcare costs in the United States.

## Supplementary Material

Appendix 2

Appendix 1

## Figures and Tables

**TABLE 1:** Results for the baseline and current scenarios and benefits to the United States of increasing hepatitis B immunization in the top 10 countries over the period 2000–2070.

	Immigrants w/CHB	Costs ($ Millions)	QALYs (millions)	CC	DC	HCC	HBV deaths
Totals							
Baseline	1,773,057	6,831,190	1054.62	131,765	36,762	121,430	184,761
Current	1,304,371	6,826,965	1054.98	109,706	29,345	97,024	149,179
WHO	1,287,610	6,826,822	1054.99	109,199	29,134	96,315	148,190
Comparative benefits							
Current vs. Baseline	−468,686	−4225	0.36	−22,059	−7417	−24,406	−35,582
WHO vs. Baseline	−485,448	−4368	0.37	−22,566	−7628	−25,115	−36,571
WHO vs. Current	−16,762	−143	0.01	−507	−211	−709	−989

*Note:* ‘top 10 countries’ refers to China, Vietnam, the Philippines, India, the Dominican Republic, Taiwan, South Korea, Mexico, Nigeria and Haiti. Abbreviations: CC, Compensated Cirrhosis; DC, Decompensated Cirrhosis; HBV, Hepatitis B; HCC, Hepatocellular Carcinoma; QALYs, Quality-Adjusted Life Years.

**TABLE 2:** Comparative benefits of policies over the Time Period 2000–2070.

	Total Immigrants 2000–2070	Fewer immigrants w/CHB	Costs saved (millions)	QALYs gained	CC averted	DC averted	HCC averted	HBV deaths averted
Benefits of ‘Current’ over ‘Baseline’								
China	21,155,343	101,550	799	68,259	4354	1395	4735	6929
Dominican Republic	2,997,204	407	5	540	32	9	33	48
Haiti	1,478,384	2593	23	1752	113	39	130	184
India	14,738,243	35,264	290	22,583	1476	504	1683	2430
Mexico	35,066,946	4474	43	4605	293	86	303	445
Nigeria	2,349,482	43,176	474	45,450	2736	1089	2789	4581
Philippines	6,361,713	267,821	2433	196,022	12,159	4024	13,736	19,531
South Korea^a^	4,741,272	−75	0	−61	−4	−1	−4	−6
Taiwan	1,652,176	70	0	24	2	1	2	3
Vietnam	2,880,467	13,406	157	15,842	899	272	999	1438
Total Top 10	93,421,228	468,686	4225	355,017	22,059	7417	24,406	35,582
Rest of World	78,903,154	395,850	3568	299,846	18,631	6264	20,613	30,053
Total	172,324,382	864,536	7794	654,862	40,690	13,681	45,018	65,635
Benefits of ‘WHO’ over ‘Current’								
China^b^	21,155,343	-	-	-	-	-	-	-
Dominican Republic	2,997,204	17	0.2	13	1	0	1	1
Haiti	1,478,384	1028	8.4	536	30	13	42	59
India	14,738,243	989	7.4	440	27	11	37	52
Mexico	35,066,946	45	0.4	27	2	1	2	3
Nigeria	2,349,482	2959	27.2	1950	103	42	146	207
Philippines	6,361,713	11,333	95.1	5827	329	138	458	636
South Korea^b^	4,741,272	-	-	-	-	-	-	-
Taiwan^b^	1,652,176	-	-	-	-	-	-	-
Vietnam	2,880,467	391	4.2	314	16	6	22	31
Total Top 10	93,421,228	16,762	142.9	9108	507	211	709	989
Rest of World	78,903,154	14,157	120.7	7693	428	178	599	835
Total	172,324,382	30,919	263.6	16,800	935	389	1307	1825

*Note:* ‘top 10’ refers to China, Vietnam, the Philippines, India, the Dominican Republic, Taiwan, South Korea, Mexico, Nigeria and Haiti all together. Abbreviations: CC, Compensated Cirrhosis; DC, Decompensated Cirrhosis; HBV, Hepatitis B; HCC, Hepatocellular Carcinoma; QALYs, Quality-Adjusted Life Years.

a South Korea had slightly higher vaccination rates in 2000 than in some of the immediate years following it which leads to the conclusion that if their rates were frozen at 2000, they might have had fewer cases of hepatitis B in a few of the following years. See Appendix S1 for more details.

b Taiwan, Korea and China have already exceeded the WHO vaccination goals as of 2020.

## Data Availability

The data that support the findings of this study are available from the corresponding author upon reasonable request.

## Abbreviations:

GHSS: Global Health Sector Strategy
HBV: Hepatitis B Virus
QALY: quality-adjusted life-year
WHO: World Health Organization
