# Supplementary material for: Modelling the potential impact of global hepatitis B vaccination on the burden of chronic hepatitis B in the United States
Source: J Viral Hepat. Author manuscript; Available in PMC 2024 Nov 5. (PMC11534504; doi:10.1111/jvh.13982)
Supplement: Appendix 2 [file NIHMS2011358-supplement-Appendix_2.docx]

# Appendix 2: Additional Methodological Details and Results

## Additional Methodological Details

**Markov model of hepatitis B disease**

**Appendix Figure 1. Markov Schematic**

Early Detected HCC

HBsAg loss

Liver

Transplantation

Inactive CHB

HBsAg-positive

Active CHB

HBeAg-negative

Active CHB

HBeAg-positive

Cirrhosis

Viral Suppression Decompensated

Cirrhosis

Viral Suppression

CHB

Viral Suppression Cirrhosis

Decompensated

Cirrhosis

Hepatocellular Carcinoma

Treated

Active CHB

HBeAg-positive

Treated

Active CHB

HBeAg-negative

**Appendix Table 1: Key cost and utility inputs**

| **Variable** | **Base Case** | **Range** | **References** |
| --- | --- | --- | --- |
| **Linkage to and Treatment Costs** |  |  |  |
| Antiviral drug costs per year* | $387 | $325-$16,464† | Redbook (May, 2022) [24] |
| Total annual monitoring costs‡ | $221 | $111-332 | Medicare reimbursement |
| Clinic visit x 2 | $74 | $37-$111 | Medicare reimbursement |
| ALT x 2 | $7 | $4-$11 | Medicare reimbursement |
| HBV DNA x 1 | $59 | $29-$88 | Medicare reimbursement |
| Annual Disease Management Costs** |  |  |  |
| Chronic Hepatitis B | $1,945 | $202-$7,816 | Liu et al. 2012 [13] |
| Cirrhosis | $5,792 | $202-$7,096 | Liu et al. 2012 [13] |
| Decompensated cirrhosis | $15,340 | $4,901-$37,081 | Liu et al. 2012 [13] |
| Symptom detected HCC | $96,345 | $77,076-$115,614 | Parikh et al. 2020 |
| Screen detected HCC non-cirrhosis | $40,167 | $32,134-$48,200 | Parikh et al. 2020 |
| Screen detected HCC cirrhosis | $85,256 | $68,205-$102,307 | Parikh et al. 2020 |
| Liver Transplantation 1st year | $208,954 | $167,163-$250,746 | Liu et al. 2012 [13] |
| Liver Transplantation 2nd year | $26,085 | $23,958-$35,937 | Liu et al. 2012 [13] |
| **Health State Utilities** |  |  |  |
| Active CHB | 0.91 | (0.80-0.92) | Woo et al. (EQ-5D) [16] |
| Cirrhosis | 0.88 | (0.78-0.88) | Woo et al. (EQ-5D) [16] |
| Inactive CHB | 1.00 | (0.90-1.00 | Assumption |
| Decompensated cirrhosis | 0.73 | (0.49-0.82) | Woo et al. (EQ-5D) [16] |
| Symptom detected HCC | 0.67 | (0.54-0.80) | Parikh et al. 2020 |
| Screen detected HCC non-cirrhosis | 0.81 | (0.65-0.85) | Woo et al. (EQ-5D) [16] |
| Screen detected HCC cirrhosis | 0.70 | (0.66-0.84) | Parikh et al. 2020 |
| Liver Transplantation | 0.84 | (0.72-0.84) | Woo et al. (EQ-5D) [16] |
| HBsAg seroclearance | 1.00 | (0.95-1.00) | Assumption |
| Viral suppression | 1.00 | (0.95-1.00) | Assumption |
|  |  |  |  |

* Assuming 60% on generic TDF and 40% on generic ETV [12].

** Adjusted to 2022 USD using the 2020 Medical Consumer Price Index (CPI) [14].

† This is the range for one-way sensitivity analysis, but for the probabilistic sensitivity analysis, it varies from $325 to $1,460.

‡ Annual monitoring is the total cost including 2x clinic visit, 2x ALT and 1x HBV DNA level as recommended by AASLD [25].

Abbreviations: AFP, alpha fetoprotein; ALT, alanine aminotransferase; CBC, complete blood count; CHB, chronic hepatitis B; HBeAg, hepatitis B e antigen; LFT, liver function tests; OPTN, Organ Procurement and Transplantation Network.

For all other healthcare costs by age, we used estimates from Jiao and Basu(1) and inflated them to 2022 values using the GDP deflator.

**Appendix Table 2. Natural History Transition Estimates**

| Transition (per year) | Natural History Estimate | Range | Reference |
| --- | --- | --- | --- |
| From Active CHB HBeAg-positive |  |  |  |
| HBsAg loss | 0.60% | (0.3-0.9) | [1] |
| To Cirrhosis | 1.60% | (1.3-1.9) | [2] |
|  |  |  |  |
| To HCC | 1.47% | (0.40-2.55) | [3] |
|  |  |  |  |
| To HBV-related Death | 0.11% | (0.09-0.14) | [3] |
| Inactive | 7% | (4.0-10.0) | [4] |
| From Active CHB HBeAg-negative |  |  |  |
| HBsAg loss | 0.60% | (0.3-0.9) | [1] |
| To Active HBeAg-positive (reversion) | 0.16% | (0.08-0.24) | [5-7] |
| To Cirrhosis | 2.80% | (1.3-4.3) | [2] |
| To HCC | 0.72% | (0.21-1.23) | [3] |
|  |  |  |  |
| To HBV-related Death | 0.11% | (0.09-0.14) | [3] |
| Inactive | 1.60% | (0.0-6.0) | [4] |
| From Compensated Cirrhosis |  |  |  |
| To HBsAg loss | 0.60% | (0.3-0.9) | [1] |
| To Decompensated Cirrhosis | 3.90% | (1.95-5.85) | [8] |
| To HCC | 3.16% | (2.58-3.74) | [3] |
| To HBV-related Death | 4.89% | (3.16-6.63) | [3] |
| To Viral Suppression | 6.30% | (3.15-9.45) | [9] |
| From Decompensated Cirrhosis |  |  |  |
| To Liver Transplantation | 1.20% | (1.0-3.0) | [10] |
| To HCC | 7.10% | (3.55-10.65) | [8] |
|  |  |  |  |
| To HBV-related Death | 15% | (7.50-22.5) | [8] |
| From HCC |  |  |  |
| To Liver Transplantation | 7% | (5.0-9.0) | [10] |
| To HBV-related Death | 42.5% |  | [11] |
| From Viral Suppression Cirrhosis |  |  |  |
| HBsAg loss | 1% | (0.5-1.5) | [1] |
| To HCC | 1.58% | (1.29-1.87) | [3] |
| To HBV-related Death | 2.44% | (1.58-3.31) | [3] |
| From Liver Transplantation Decompensated Cirrhosis |  |  |  |
| To HBV-related death year 1 | 17% | (8.5-48.0) | [12] |
| To HBV-related death year 2+ | 2.50% | (1.25-24.0) | [12] |
| From Liver Transplantation HCC |  |  |  |
| To HBV-related death year 1 | 16% | (8.0-48.0) | [12] |
| To HBV-related death year 2+ | 2% | (2.0-25.0) | [12] |
| From Inactive |  |  |  |
| To HBsAg loss | 1.25% | (0.98-1.54) | [13] |
| To active CHB, HBeAg-negative |  |  |  |
| Age-group < 30 | 0.89% | (0.71-1.06) | [14] |
| Age-group 30-39 | 1.53% | (1.23-1.83) | [14] |
| Age-Group 40-49 | 2.14% | (1.71-2.56) | [14] |
| Age-Group 50+ | 1.51% | (1.21-1.81) | [14] |
| To Cirrhosis |  |  |  |
| Age-group <30 | 0.03% | (0.024-0.036) | [14] |
| Age-group 30-39 | 0.25% | (0.20-0.30) | [14] |
| Age-Group 40-49 | 0.53% | (0.43-0.63) | [14] |
| Age-Group 50+ | 0.71% | (0.57-0.85) | [14] |
| To HCC | 0.17% | (0.02-0.62) | [15] |
| From HBsAg loss |  |  |  |
| To Cirrhosis | 0.28% | (0.14-0.42) | [14, 16] |
| To HCC | 0.09% | (0.045-0.136) | [17] |
| Gender |  |  |  |
| Relative Progression Rates for females* | 0.5 | (0.25-1.0) | [18-20] |
| Fraction of chronic HBV cases that are Male | 65% | (0.50-0.80) | [21] |

A 50% reduction in disease progression estimates was applied for females

Abbreviations: CHB, chronic hepatitis B; HBeAg, hepatitis B e antigen; HBsAg, hepatitis B surface antigen; HBV, hepatitis B virus; HCC, hepatocellular carcinoma

**Appendix Table 3. Treatment transition estimates**

| Transition (per year) | Treatment Estimate | Range | Reference |
| --- | --- | --- | --- |
| From Active CHB HBeAg-positive |  |  |  |
| HBsAg loss | 3% | (1.5-4.5) | [22] |
| To Cirrhosis | 0 | 0 | assumption |
|  |  |  |  |
| To HCC | 0.44% | (0.12-0.765) | (70% reduction)[23, 24] |
|  |  |  |  |
| To HBV-related Death | 0 | 0 | assumption |
| To Drug Resistance | 0.01% | (0.0-0.01) | [25-27] |
| To Viral Suppression | 76% | (65.0-85.0) | [22] |
| From Active CHB HBeAg-negative |  |  |  |
| HBsAg loss | 1% | (0.5-1.5) | [22] |
| To Cirrhosis | 0 | 0 | assumption |
| To HCC | 0.22% | (0.063-0.369) | (70% reduction)[23, 24] |
|  |  |  |  |
| To HBV-related Death | 0 | 0 | assumption |
| To Drug Resistance | 0.01% | (0.0-0.01) | [25-27] |
| To Viral Suppression | 93% | (65.0-99.0) | [22] |
| From Compensated Cirrhosis |  |  |  |
| To HBsAg loss | 1.70% | (0.85-2.55) | [28] |
| To Decompensated Cirrhosis | 1.80% | (0.90-2.70) | (50% reduction) |
| To HCC | 1.60% | (1.25-1.75) | (50% reduction) [29] |
| To HBV-related Death | 2.40% | (1.58-3.30) | (50% reduction) |
| To Viral Suppression | 78% | (65.0-78.0) | [29] |
| To Drug Resistance | 0.01% | (0.0-0.01) | [25-27] |
| From Decompensated Cirrhosis |  |  |  |
| To Liver Transplantation | 1.20% | (0.60-1.80) | [10] |
| To HCC | 3.50% | (1.75-5.25) | (50% reduction) [29] |
|  |  |  |  |
| To HBV-related Death | 7.50% | (3.75-11.25) | (50% reduction) |
| To Viral Suppression | 78% | (65.0-78.0) | [29] |
| To Drug Resistance | 0.01% | (0.0-0.01) | [25-27] |
| From HCC |  |  |  |
| To Liver Transplantation | 7% | (5.0-9.0) | [10] |
| To HBV-related Death | 31.1% | (26.4-31.1) | (27% reduction)[30] |
| From Viral Suppression CHB |  |  |  |
| HBsAg loss | 1.50% | (0.07-2.2) | [22] |
| To HCC | 0.06% | (0.03-0.09) | (70% reduction)[23, 24] |
|  |  |  |  |
| From Viral Suppression Cirrhosis |  |  |  |
| HBsAg loss | 1.50% | (0.07-2.2) | [22] |
| To HCC | 0.80% | (0.40-1.20) | (50% reduction) |
| To HBV-related Death | 1.20% | (0.60-1.80) | (50% reduction) |
| From Viral Suppression Decompensated Cirrhosis |  |  |  |
| To HCC | 3% | (1.5-4.5) | [31] |
| To HBV-related Death | 6.10% | (3.05-9.15) | [31] |
| From Liver Transplantation for Decompensated Cirrhosis |  |  |  |
| To HBV-related death year 1 | 17%-32% | (8.5-48.0) | [12] |
| To HBV-related death year 2+ | 2.50% | (1.25-24.0) | [12] |
| From Liver Transplantation for HCC |  |  |  |
| To HBV-related death year 1 | 16%-39% | (8.0-48.0) | [12] |
| To HBV-related death year 2+ | 2% | (2.0-25.0) | [12] |
| Gender |  |  |  |
| Relative Progression Rates for females* | 0.5 | (0.25-1.0) | [18-20] |
| Fraction of chronic HBV cases that are Male | 65% | (0.50-0.80) | [21] |

A 50% reduction in disease progression estimates was applied for females

**Treatment Assumptions**

The following graphs show estimates of the fraction of individuals treated under the current and WHO scenarios. Treatment rates are the number of individuals treated divided by the total population eligible for treatment (whether diagnosed or undiagnosed). The treatment assumptions are the same for all scenarios.

We assumed that the treatment status of the individual would be based on estimates of treatment in the US. Treatment rates are set at 10% and represent rates of treatment in those eligible for treatment (whether aware or unaware of their infection) and are based on estimates of treatment in the United States in the early part of this century (19) and they increase linearly to 2020 levels and then stay constant. In all scenarios, we assumed those untreated will face the natural history of disease.

**Appendix Figure 2: Assumptions of fraction treated over time**

The fraction treated is presumed to be the estimated US treatment levels, which are 10% in 2000 and 30% in 2020 and assumed to linearly increase from 10% to 30% from 2000 to 2020.

## Additional Results

Estimated annual immigrants arriving with chronic hepatitis B under the “Baseline” scenario with the “Current” scenario.

**Appendix Figure 3a: Estimated Annual Immigrants to the United States from these Top 10 Countries**

Note: The increase in immigrants from 2010-2019 is due to increasing rates of immigration. The reduction in immigrants from 2020-2022 is due to COVID-19. Immigration in 2023 is assumed to follow pre-pandemic trends.

**Appendix Table 4: Total Immigration and Immigration with Chronic Hepatitis B under Scenarios**

|  | **Total Immigrants 2000-2019** | **Total Immigrants 2020-2040** | **Total Immigrants 2041-2070** | **Total Immigrants w/CHB 2000-2019**  **Baseline Scenario** | **Total Immigrants w/CHB 2020-2040**  **Baseline Scenario** | **Total Immigrants w/CHB 2040-2070**  **Baseline Scenario** | **Total Immigrants w/CHB 2000-2070**  **Baseline Scenario** | **Total Immigrants w/CHB 2000-2019**  **Current Scenario** | **Total Immigrants w/CHB 2020-2040**  **Current Scenario** | **Total Immigrants w/CHB 2040-2070**  **Current Scenario** | **Total Immigrants w/CHB 2000-2070**  **Current Scenario** | **Total Immigrants w/CHB 2000-2019**  **WHO Target Scenario** | **Total Immigrants w/CHB 2020-2040**  **WHO Target Scenario** | **Total Immigrants w/CHB 2040-2070**  **WHO Target Scenario** | **Total Immigrants w/CHB 2000-2070**  **WHO Target Scenario** |
| --- | --- | --- | --- | --- | --- | --- | --- | --- | --- | --- | --- | --- | --- | --- | --- |
| **China** | 2,980,838 | 7,188,772 | 10,985,732 | 193,622 | 249,417 | 134,608 | 577,647 | 189,621 | 203,883 | 82,593 | 476,097 | 189,621 | 203,883 | 82,593 | 476,097 |
| **Dominican Republic** | 763,215 | 875,172 | 1,358,816 | 10,562 | 3,723 | 978 | 15,263 | 10,494 | 3,553 | 810 | 14,857 | 10,494 | 3,550 | 797 | 14,841 |
| **Haiti** | 507,713 | 379,624 | 591,046 | 8,554 | 5,571 | 5,911 | 20,036 | 8,543 | 5,218 | 3,682 | 17,443 | 8,543 | 5,088 | 2,784 | 16,415 |
| **India** | 3,022,579 | 4,820,536 | 6,895,129 | 44,381 | 45,041 | 38,434 | 127,856 | 43,832 | 37,935 | 10,824 | 92,591 | 43,832 | 37,780 | 9,990 | 91,602 |
| **Mexico** | 8,620,351 | 10,415,007 | 16,031,588 | 51,734 | 19,351 | 4,649 | 75,734 | 51,409 | 17,526 | 2,326 | 71,261 | 51,409 | 17,516 | 2,291 | 71,216 |
| **Nigeria** | 345,220 | 697,203 | 1,307,058 | 25,383 | 41,131 | 54,397 | 120,911 | 24,707 | 32,177 | 20,851 | 77,735 | 24,707 | 31,671 | 18,398 | 74,776 |
| **Philippines** | 1,392,004 | 1,923,957 | 3,045,752 | 128,346 | 166,652 | 276,804 | 571,802 | 122,163 | 107,945 | 73,872 | 303,980 | 122,163 | 106,830 | 63,654 | 292,647 |
| **South Korea** | 1,560,598 | 1,250,548 | 1,930,127 | 55,775 | 12,443 | 2,554 | 70,772 | 55,834 | 12,486 | 2,529 | 70,849 | 55,834 | 12,486 | 2,529 | 70,849 |
| **Taiwan** | 470,131 | 463,812 | 718,233 | 35,204 | 10,779 | 3,073 | 49,056 | 35,204 | 10,753 | 3,028 | 48,985 | 35,204 | 10,753 | 3,028 | 48,985 |
| **Vietnam** | 602,569 | 876,412 | 1,401,487 | 64,868 | 49,113 | 29,998 | 143,979 | 63,878 | 44,305 | 22,390 | 130,573 | 63,878 | 44,222 | 22,083 | 130,183 |
| **Total Top 10** | 20,265,218 | 28,891,042 | 44,264,968 | 618,429 | 603,221 | 551,408 | 1,773,058 | 605,684 | 475,782 | 222,905 | 1,304,371 | 605,684 | 473,778 | 208,147 | 1,287,609 |
| **Rest of World** | 17,115,913 | 24,401,246 | 37,385,995 | 522,322 | 509,478 | 465,717 | 1,497,517 | 511,558 | 401,843 | 188,265 | 1,101,666 | 511,558 | 400,151 | 175,800 | 1,087,509 |
| **Total** | 37,381,131 | 53,292,288 | 81,650,962 | 1,140,751 | 1,112,699 | 1,017,124 | 3,270,574 | 1,117,242 | 877,626 | 411,170 | 2,406,038 | 1,117,242 | 873,929 | 383,948 | 2,375,119 |

Abbreviations: CHB, chronic hepatitis B

**Appendix Table 5. Results for the three scenarios and cumulative benefits to the US of increasing global immunization over the time period 2000-2070 for the entire globe.**

|  | **immigrants w/CHB** | **Costs ($ Millions)** | **QALYs (millions)** | **CC** | **DC** | **HCC** | **HBV Deaths** |
| --- | --- | --- | --- | --- | --- | --- | --- |
| Baseline | 3,270,574 | 12,600,783 | 1,945.35 | 243,054 | 67,811 | 223,989 | 340,810 |
| Current | 2,406,038 | 12,592,990 | 1,946.01 | 202,363 | 54,130 | 178,970 | 275,175 |
| WHO | 2,375,119 | 12,592,726 | 1,946.03 | 201,429 | 53,741 | 177,663 | 273,350 |
| Comparative Benefits |  |  |  |  |  |  |  |
| Current vs. Baseline | -864,536 | -7,794 | 0.65 | -40,690 | -13,681 | -45,018 | -65,635 |
| WHO vs Current | -30,919 | -264 | 0.02 | -935 | -389 | -1,307 | -1,825 |

**Appendix Figure 4a: Breakdown of annual immigrants arriving from top 10 countries with chronic hepatitis B under baseline scenario**

* CHB = Chronic Hepatitis B

**Appendix Figure 4b: Breakdown of annual immigrants arriving from top 10 countries with chronic hepatitis B under current scenario**

* CHB = Chronic Hepatitis B

**Appendix Figure 4c: Breakdown of annual immigrants arriving from top 10 countries with chronic hepatitis B under WHO scenario**

* CHB = Chronic Hepatitis B

**Appendix Figure 5a: Estimated Total immigrants with Chronic Hepatitis B from a restricted historical time frame 2000-2020**

**Appendix Figure 5b: Estimated total immigrants with Chronic Hepatitis B from an intermediate timeframe of 2020-2040**

**Appendix Figure 5c: Estimated total immigrants with Chronic Hepatitis B from the entire timeframe of 2000-2070**

**Appendix Figure 6a: Impact of Current policy: averted Chronic Hepatitis B infections from year 2000 to 2070 by country and broken out by time period**

**Appendix Figure 6b: Impact of WHO policy: averted Chronic Hepatitis B infections from year 2000 to 2070 by country and broken out by time period**

Note: Since the WHO and Current vaccination levels are equivalent between 2000 and 2020, the difference during that time period is zero.

**Appendix Figure 7a: Annual averted deaths due to fewer immigrants arriving from 2000-2070 from top 10 countries with chronic hepatitis B because of current policy compared to baseline.**

**Appendix Figure 7b: Annual averted deaths due to fewer immigrants arriving from 2000-2070 from top 10 countries with chronic hepatitis B because of WHO policy compared to current policy.**

**Appendix Figure 8a: Annual QALYs gained due to fewer immigrants arriving from 2000-2070 from top 10 countries with chronic hepatitis B because of current policy compared to baseline policy.**

**Appendix Figure 8b: Annual QALYs gained due to fewer immigrants arriving from 2000-2070 from top 10 countries with chronic hepatitis B because of WHO policy compared to current policy.**

**Appendix Figure 9a: Annual costs saved due to fewer immigrants arriving from 2000-2070 from top 10 countries with chronic hepatitis B because of current policy compared to baseline policy.**

**Appendix Figure 9b: Annual costs saved due to fewer immigrants arriving from 2000-2070 from top 10 countries with chronic hepatitis B because of WHO policy compared to current policy.**

**Appendix Figure 10: Estimated number of prevalent persons with Chronic Hepatitis B in the United States under each scenario.**

**Appendix Table 6: *Sensitivity Analysis with lower treatment rates.* Cumulative benefits of “Current” over “Baseline” from 2000 to 2070**

|  | **Fewer Immigrants w/CHB** | **Costs Saved ($ Millions)** | **QALYs Gained** | **CC Averted** | **DC Averted** | **HCC Averted** | **HBV Deaths Averted** |
| --- | --- | --- | --- | --- | --- | --- | --- |
| China | 101,550 | 871 | 80,644 | 5,041 | 1,773 | 5,064 | 8,038 |
| Dominican Republic | 407 | 5 | 631 | 36 | 11 | 35 | 55 |
| Haiti | 2,593 | 26 | 2,077 | 131 | 49 | 139 | 214 |
| India | 35,264 | 319 | 26,738 | 1,719 | 641 | 1,803 | 2,823 |
| Mexico | 4,474 | 47 | 5,413 | 337 | 109 | 324 | 515 |
| Nigeria | 43,176 | 469 | 44,276 | 2,650 | 1,031 | 2,736 | 4,434 |
| Philippines | 267,821 | 2,659 | 231,839 | 14,110 | 5,118 | 14,686 | 22,670 |
| South Korea | -75 | 0 | -69 | -4 | -1 | -4 | -6 |
| Taiwan | 70 | 0 | 29 | 2 | 1 | 2 | 3 |
| Vietnam | 13,406 | 170 | 18,588 | 1,041 | 344 | 1,064 | 1,661 |
| Total Top 10 | 468,686 | 4,565 | 410,165 | 25,065 | 9,077 | 25,849 | 40,406 |
| Rest of World | 395,850 | 3,855 | 346,424 | 21,170 | 7,666 | 21,832 | 34,127 |
| Total | 864,536 | 8,420 | 756,589 | 46,234 | 16,743 | 47,681 | 74,533 |

Note: Taiwan, South Korea, and China have already exceeded the WHO vaccination goals.

**Appendix Table 7: *Sensitivity Analysis with lower treatment rates.* Cumulative benefits of Achieving the WHO 2030 Vaccination Targets over the Time Period 2000-2070 as Compared to the Current Strategy.**

|  | **Fewer Immigrants w/CHB** | **Costs Saved ($ Millions)** | **QALYs Gained** | **CC Averted** | **DC Averted** | **HCC Averted** | **HBV Deaths Averted** |
| --- | --- | --- | --- | --- | --- | --- | --- |
| China | - | - | - | - | - | - | - |
| Dominican Republic | 17 | 0.2 | 15 | 1 | 0 | 1 | 1 |
| Haiti | 1,028 | 9.3 | 636 | 36 | 16 | 45 | 69 |
| India | 989 | 8.2 | 523 | 32 | 15 | 40 | 61 |
| Mexico | 45 | 0.4 | 32 | 2 | 1 | 2 | 3 |
| Nigeria | 2,959 | 30 | 2,308 | 123 | 54 | 156 | 240 |
| Philippines | 11,333 | 105.1 | 6,922 | 389 | 176 | 489 | 738 |
| South Korea | - | - | - | - | - | - | - |
| Taiwan | - | - | - | - | - | - | - |
| Vietnam | 391 | 4.6 | 372 | 19 | 8 | 24 | 36 |
| Total Top 10 | 16,762 | 157.8 | 10,808 | 601 | 269 | 756 | 1,148 |
| Rest of World | 14,157 | 133.3 | 9,128 | 508 | 227 | 639 | 970 |
| Total | 30,919 | 291.1 | 19,936 | 1,108 | 496 | 1,395 | 2,118 |

Note: Taiwan, South Korea, and China have already exceeded the WHO vaccination goals.

**Appendix Figure 11: Cumulative cost** **Savings from 2000-2070 Due to Progress since Baseline**

Appendix 1 References

1. Jiao B, Basu A. Catalog of Age- and Medical Condition—Specific Healthcare Costs in the United States to Inform Future Costs Calculations in Cost-Effectiveness Analysis. Value in Health. 2021;24(7).

2. CDA Foundation. Countries Dashboard [Internet]. [cited 2022 Dec 11]. Available from: https://cdafound.org/polaris-countries-dashboard/
