# Supplementary material for: Modelling the potential impact of global hepatitis B vaccination on the burden of chronic hepatitis B in the United States
Source: J Viral Hepat. Author manuscript; Available in PMC 2024 Nov 5. (PMC11534504; doi:10.1111/jvh.13982)
Supplement: Appendix 1 [file NIHMS2011358-supplement-Appendix_1.docx]

**Appendix 1: Country Model Detailed Methodology and Additional Results**

**Country Models**

1. **Common Data for all Models**

The HBV prevalence models are developed to estimate the number of HBV-infected immigrants from the top 10 prevalent countries to the United States after 2000.

**Vaccination Coverage**

For all Models, we used WHO/UNICEF estimates of birth dose and 3-dose Hep B vaccination coverage. Typically, for most countries, these estimates start around 2000-2005 and continue through 2020.

In some rare cases (China), we used some additional older estimates of vaccination coverage prior to the availability of WHO/UNICEF vaccination coverage estimates.

**Model Parameters**

For all models, we assumed 90% probability of transmission from an HBeAg+ mother and 10% probability of transmission from an HBeAg- mother (assumption from Goldstein et. al. and Hadler et al.). We also assumed that the vaccines were 95% effective (assumption from Goldstein et. al. and Hadler et al.).

For each model, we obtained the prevalence of maternal HBsAg, HBeAg, as well as Anti-HBc at 5 and 30 years old from published articles. Given that those data were collected from various studies and the years when surveys were performed might not perfectly match the starting years of models’ simulations, and the ages of the majority of the immigrants ranged from 20 to 40 years old, we first assume the baseline parameters within each model could represent the same population, then assumed our model to start from 1960 to ensure the model could simulate the prevalence for the 40-year-olds in 2000. Given that the model's starting year is 1960, in order to ensure that a model’s simulation is closer to reality, the earlier the data is considered the better. For most of the included countries, baseline HBsAg prevalence was selected from studies published yielding from 1985 to 2000.

1. **Country-Specific Data**

**2.1 China**

**Model Set-up**

China introduced a national HBV vaccination program in 1985, and after 7 years in 1992, the timely birth dose of HBV vaccination was designed to be one of the routine vaccinations but it was based on self preference and the costs should be met by parents. In 2002, allied with GAVI, the Chinese government launched free three-dose HBV vaccinations for newborns. Cui et al. recorded the 3-dose HBV vaccination rates each year from 1985 to 2013. As estimated coverage rates from WHO/UNICEF are available from 2000 to 2021, data from Cui were supplemented to draw a full image of vaccination history in China from 1985 to 2021. (Figure 2.1.1S)

For this model in China, the true prevalence surveyed in 1992 was used to adjust the precision of the model. A previous study conducted by Zu et al. in 2017 summarized three national serosurveys, which were performed in 1992, 2006, and 2014, respectively. Every included study calculated the HBsAg prevalence among the general population after the implementation of the universal HBV vaccination program in 1992. In this study, the modeled results will be used to compare with the results from the three serosurveys from Zu’s study for validation. (Table 2.1.1S)

In China, the first two national serosurveys were conducted in 1979 and 1992, respectively, showing HBsAg prevalence of 9.05% and 9.75% (Liu et al., 2022). Multiple studies investigating the maternal HBsAg prevalence were performed after 2015 and the results declined from 7.30% in 2015 to 5.44% in 2021 (Liu et al., 2021). As no data showed maternal HBsAg prevalence as early as 1992 in China, given the downward trend demonstrated in the study by Liu et al. in 2021, we adapted the HBsAg prevalence among the general population measured in 1992 as our baseline prevalence. Hadler et al., in 2013, derived parameters for Anti-Hbc positive rates among people at age 5 and age 30 from serosurveys for a modeling study, and these numbers were adapted for this modeling study. (Table 2.1.2S)

**Vaccination Coverage**

**Figure 2.1.1S Comparison between vaccination coverage under the baseline and current scenarios**


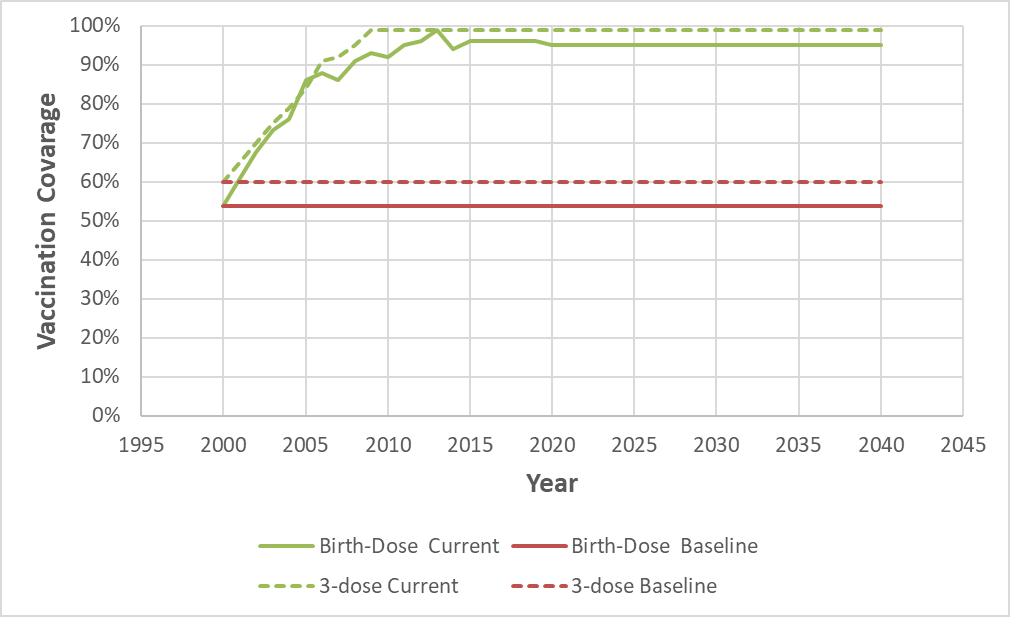


* WHO scenario not shown since China has exceeded WHO vaccination goals

**Table 2.1.1S Summary of actual serosurvey data in China for comparison**

| **Age Group** | **N** | **HBsAg** | **Lower 95% CI** | **Upper 95% CI** | **Survey year** | **Source** |
| --- | --- | --- | --- | --- | --- | --- |
| 1-4 | 3288 | 9.67% | 8.66% | 10.68% | 1992 | Zu et al., 2017 |
| 5-9 | 6398 | 10.22% | 9.48% | 10.96% |  |  |
| 10-14 | 6316 | 11.27% | 10.49% | 12.05% |  |  |
| 15-19 | 4639 | 10.35% | 9.47% | 11.22% |  |  |
| 20-24 | 5691 | 9.49% | 8.73% | 10.25% |  |  |
| 25-29 | 7328 | 9.61% | 8.93% | 10.28% |  |  |
| 30-34 | 6579 | 10.64% | 9.89% | 11.39% |  |  |
| 35-39 | 6898 | 9.22% | 8.54% | 9.90% |  |  |
| 40-49 | 8680 | 9.31% | 8.70% | 9.92% |  |  |
| 50-59 | 5885 | 7.58% | 6.90% | 8.25% |  |  |
| 1-4 | 16376 | 1.08% | 0.92% | 1.24% | 2006 |  |
| 5-9 | 11909 | 1.60% | 1.38% | 1.83% |  |  |
| 10-14 | 11844 | 3.37% | 3.04% | 3.69% |  |  |
| 15-19 | 2942 | 7.21% | 6.27% | 8.14% |  |  |
| 20-24 | 2584 | 8.17% | 7.11% | 9.22% |  |  |
| 25-29 | 4194 | 8.25% | 7.42% | 9.08% |  |  |
| 30-34 | 6215 | 7.95% | 7.28% | 8.62% |  |  |
| 35-39 | 6949 | 8.25% | 7.60% | 8.89% |  |  |
| 40-49 | 10477 | 8.40% | 7.87% | 8.93% |  |  |
| 50-59 | 8285 | 8.05% | 7.46% | 8.64% |  |  |
| 1-4 | 12681 | 0.38% | 0.27% | 0.49% | 2014 |  |
| 5-9 | 5443 | 0.75% | 0.52% | 0.98% |  |  |
| 10-14 | 4295 | 1.23% | 0.90% | 1.56% |  |  |
| 15-19 | 2618 | 1.95% | 1.42% | 2.48% |  |  |
| 20-24 | 2820 | 4.57% | 3.80% | 5.35% |  |  |
| 25-29 | 3856 | 5.06% | 4.37% | 5.75% |  |  |

**Table 2.1.2S Parameters for Model China**

| **Parameter** | **Value** | **Source** |
| --- | --- | --- |
| Maternal HBsAg prevalence | 9.75% | Xia et al., 1996 |
| Maternal HBeAg prevalence | 30.0% | Liang et al., 2009 |
| Anti-HBc prevalence at age 5 | 32.0% | Hadler et al., 2013 |
| Anti-HBc prevalence at age 30 | 55.0% | Hadler et al., 2013 |

**Model Validation**

1. Comparisons with the actual serosurvey

**Figure 2.1.2S Comparison between modeled prevalence with the reported data from Zu et al. by age in 1992, 2006 and 2014**


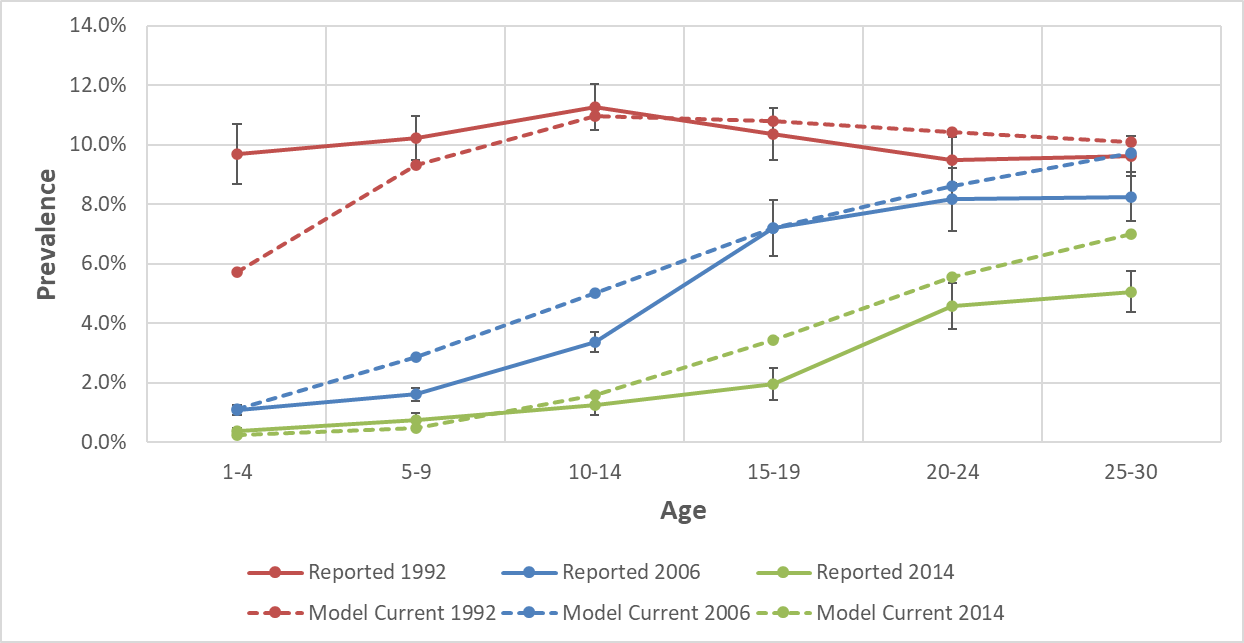


1. Comparisons with Wong et al, 2019

**Figure 2.1.3S Comparison between age-specific modeled prevalence with the overall prevalence in immigrants as estimated by Wong et al., 2019 (not stratified by age)**


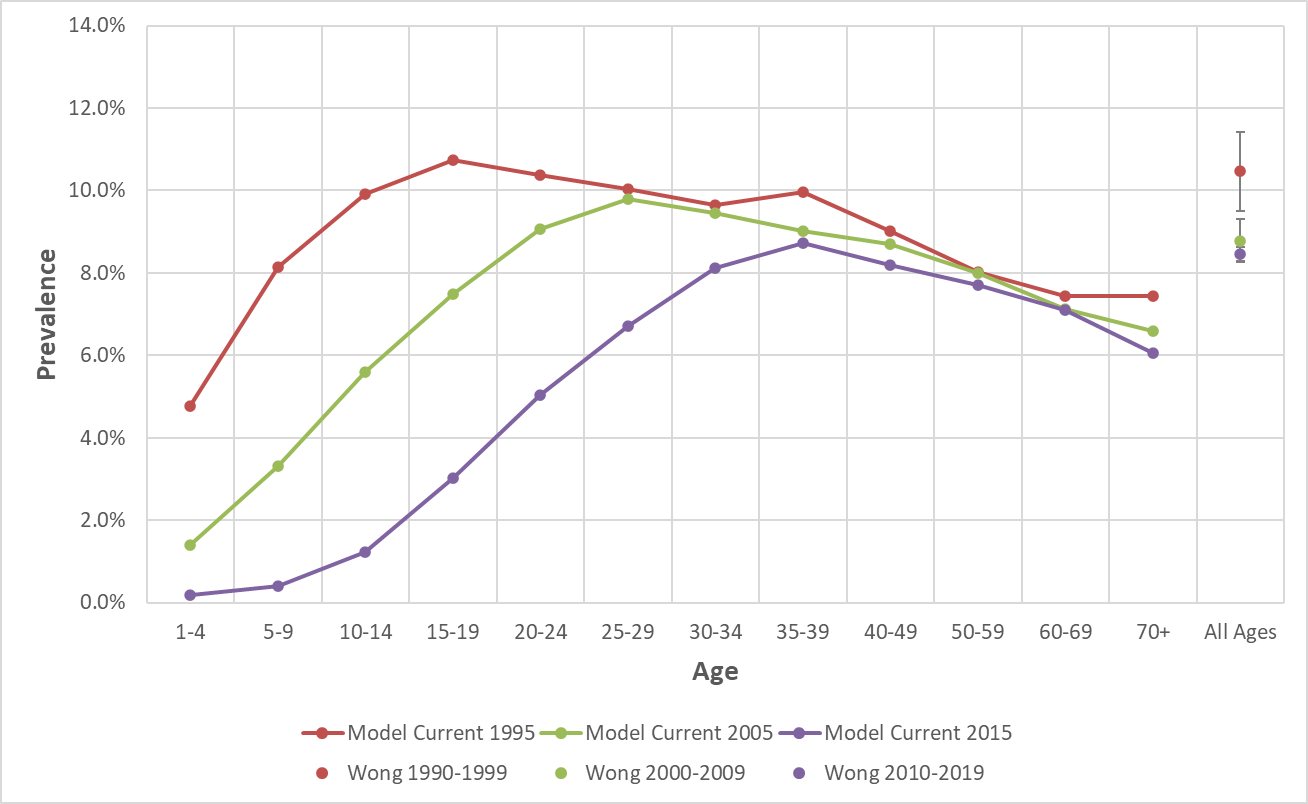


**Results**

**Figure 2.1.4S Estimated prevalence under the baseline and current scenarios for a 5-year-old in China**

**
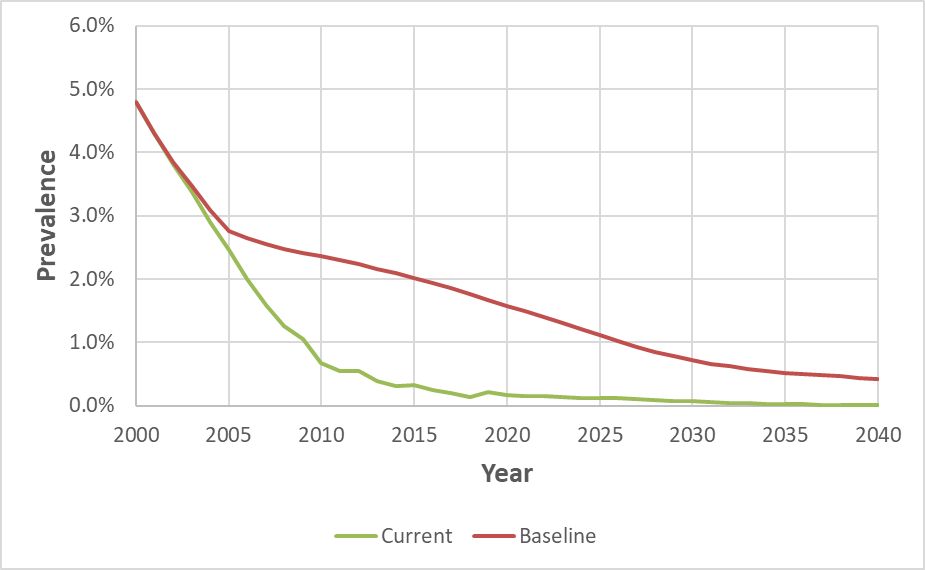
**

* WHO scenario not shown since China has exceeded WHO vaccination goals

**Figure 2.1.5S Estimated prevalence under the baseline and current scenarios for a 20-year-old in China**


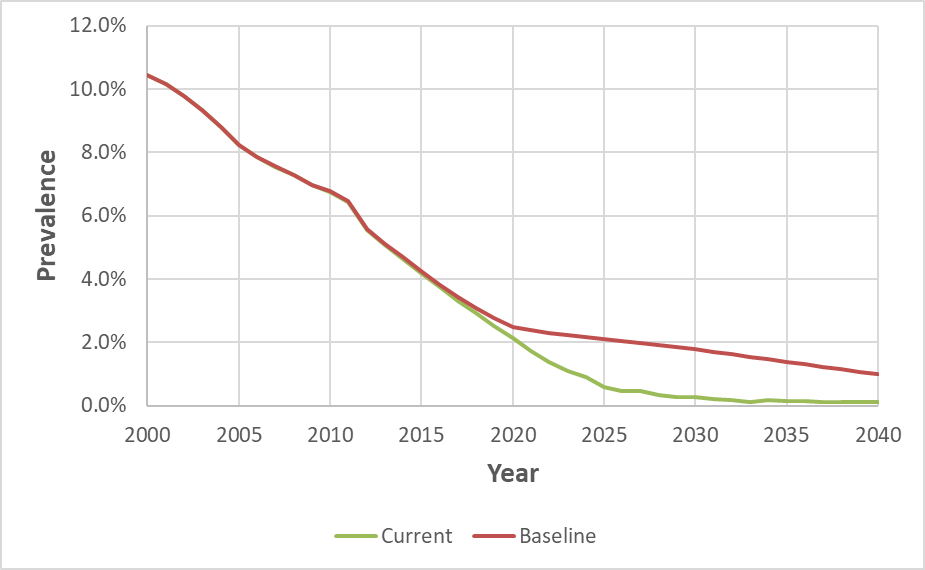


* WHO scenario not shown since China has exceeded WHO vaccination goals

**Figure 2.1.6S Estimates of immigrants with chronic hepatitis B under the baseline and current scenarios in China**


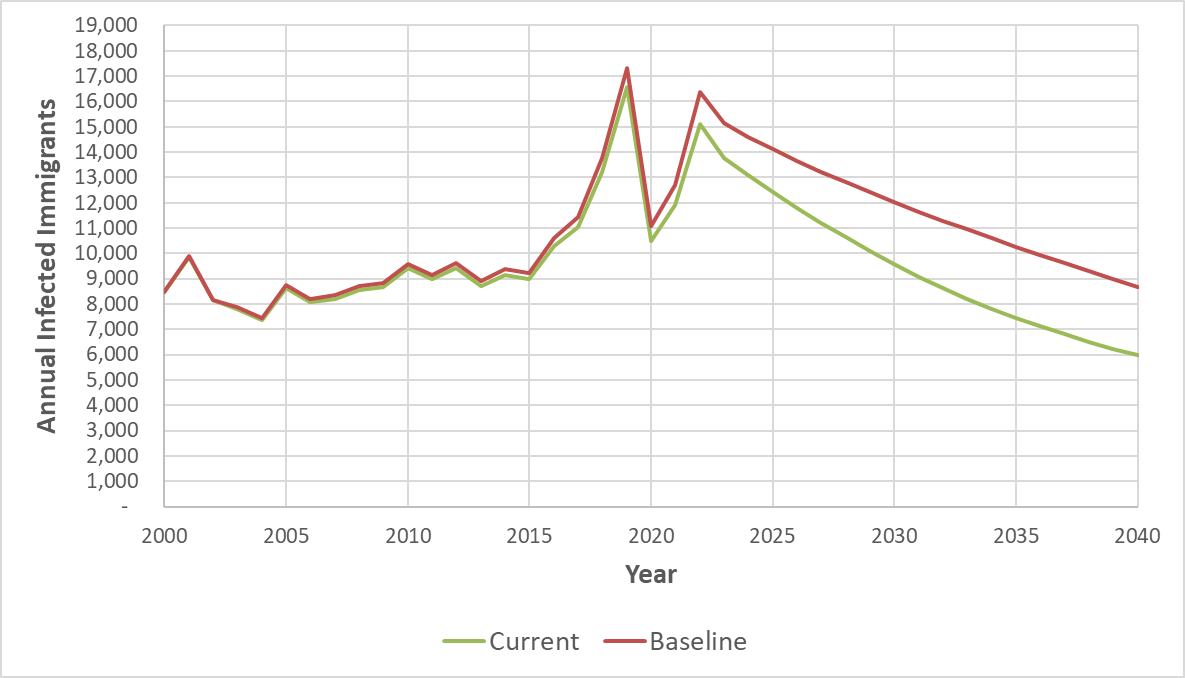
* WHO scenario not shown since China has exceeded WHO vaccination goals

**References:**

Zu J, Zhuang G, Liang P, Cui F, Wang F, Zheng H, Liang X. Estimating age-related incidence of HBsAg seroclearance in chronic hepatitis B virus infections of China by using a dynamic compartmental model. Scientific Reports. 2017 Jun 6;7(1):2912.

Liang X, Bi S, Yang W, Wang L, Cui G, Cui F, Zhang Y, Liu J, Gong X, Chen Y, Wang F. Epidemiological serosurvey of hepatitis B in China—declining HBV prevalence due to hepatitis B vaccination. vaccine. 2009 Nov 5;27(47):6550-7.

Liu J, Wang X, Wang Q, Qiao Y, Jin X, Li Z, Du M, Yan W, Jing W, Liu M, Wang A. Hepatitis B virus infection among 90 million pregnant women in 2853 Chinese counties, 2015-2020: a national observational study. The Lancet Regional Health–Western Pacific. 2021 Nov 1;16.

Liu W, Zhuang T, Xia R, Zou Z, Zhang L, Shen M, Zhuang G. Modelling the prevalence of hepatitis B towards eliminating it as a major public health threat in China. BMC Public Health. 2022 Dec;22(1):1-0.

Cui F, Shen L, Li L, Wang H, Wang F, Bi S, Liu J, Zhang G, Wang F, Zheng H, Sun X. Prevention of chronic hepatitis B after 3 decades of escalating vaccination policy, China. Emerging infectious diseases. 2017 May;23(5):765.

Hadler SC, Fuqiang C, Averhoff F, Taylor T, Fuzhen W, Li L, Xiaofeng L, Weizhong Y. The impact of hepatitis B vaccine in China and in the China GAVI Project. Vaccine. 2013 Dec 27;31:J66-72.

Xia GL, Liu CB, Cao HL, Bi SL, Zhan MY, Su CA, Nan JH, Qi XQ. Prevalence of hepatitis B and C virus infections in the general Chinese population. Results from a nationwide cross-sectional seroepidemiologic study of hepatitis A, B, C, D, and E virus infections in China, 1992. International Hepatology Communications. 1996 May 1;5(1):62-73.

**2.2 Vietnam**

**Model Set-up**

In Vietnam, the 3-dose HBV vaccination was introduced in 1998, while it was expanded to the timely birth dose in 2002. Since WHO/UNICEF estimates have included data from 2003 for HepB coverage and from 2007 for timely birth dose, administratively reported data were used to fill in the blanks from 1998 to 2002 for HepB coverage and from 2002 to 2006 for timely birth dose. (Figure 2.2.1S) Interestingly, the 3-dose coverage was quite high in 2000, leading to a high vaccination rate under the baseline scenario.

To adjust our model, we used a cross-sectional seroprevalence study undertaken by Nguyen et al. in two rural districts in Thai Binh province studying 837 Vietnamese subjects in 2006. A self-conducted serosurvey performed by Komada et al. in 2019 demonstrated the age-specific HBsAg prevalence among people ranging from 1 to 39 years old. In this study, the modeled results will be used to compare with the results from the serosurvey from Komada’s study for validation. (Table 2.2.1S)

For the Vietnamese model, a serosurvey performed in 1985 was collected. This study covered HBsAg prevalence among Vietnamese women aged 18-19, 20-20, and 30-39 and HBeAg prevalence in all HBsAg-positive subjects. Assuming that the usual gestational age for women tends to be between 18 and 39 years old, recalculation using the given sample size and number of positive cases was applied to obtain the maternal HBsAg prevalence that is needed. A study conducted in rural Vietnam demonstrated the Anti-HBc positive rate among infants (9-18 months [mean =13.8]), children (4-6 years old [mean =5.1]), adolescents (14-16 years old [mean =15.0]), and adults (25-40 years old [mean = 32.9]). In this study, Anti-HBc positive rates among people at age 5 and age 30 were adapted from the above study using Anti-HBc prevalence among children and adults, respectively. (Table 2.2.2S)

**Vaccination Coverage**

**Figure 2.2.1S Comparison between vaccination coverage under the current, baseline, and WHO scenarios**


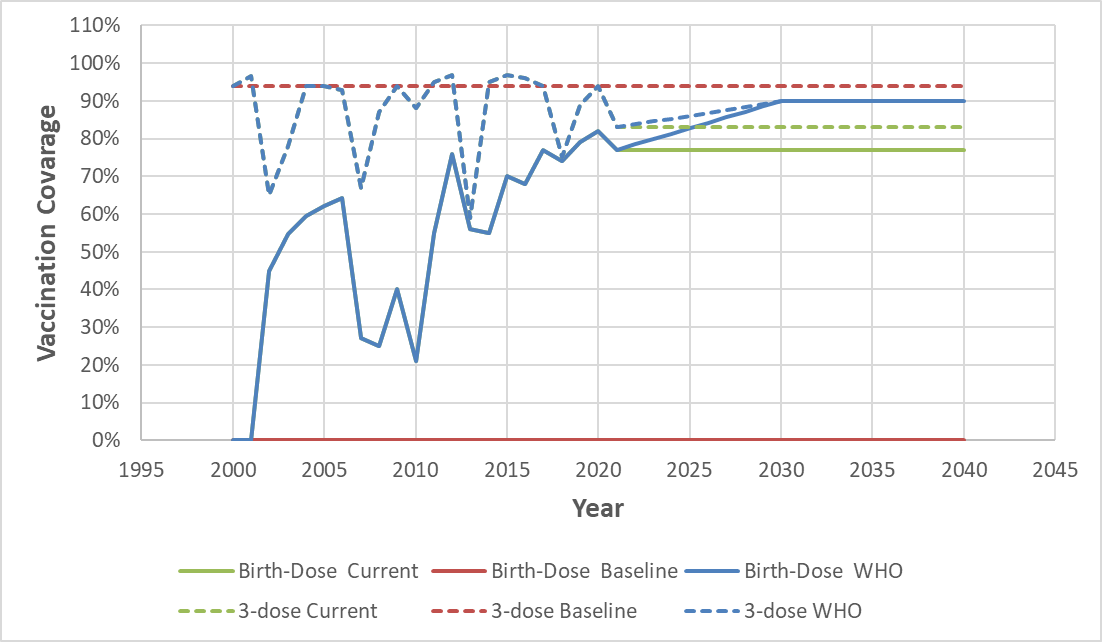


**Table 2.2.1S Summary of actual serosurvey data in Vietnam for comparison**

| **Age Group** | **N** | **HBsAg** | **Lower 95% CI** | **Upper 95% CI** | **Survey year** | **Source** |
| --- | --- | --- | --- | --- | --- | --- |
| 1-4 | 266 | 1.50% | 0.32% | 2.98% | 2019 | Komada et al, 2020 |
| 5-9 | 366 | 3.01% | 1.25% | 4.76% |  |  |
| 10-14 | 298 | 1.01% | 0.00% | 2.15% |  |  |
| 15-19 | 249 | 3.61% | 1.28% | 5.95% |  |  |
| 20-24 | 167 | 10.18% | 5.55% | 14.81% |  |  |
| 25-29 | 222 | 7.21% | 3.78% | 10.64% |  |  |
| 30-34 | 252 | 11.11% | 7.10% | 15.02% |  |  |
| 35-39 | 258 | 9.30% | 5.73% | 12.87% |  |  |

**Table 2.2.2S Parameters for Model Vietnam**

| **Parameter** | **Value** | **Source** |
| --- | --- | --- |
| Maternal HBsAg prevalence | 12.6% | Hoang et al., 1985 |
| Maternal HBeAg prevalence | 25.8% | Hoang et al., 1985 |
| Anti-HBc prevalence at age 5 | 36.4% | Hipgrave et al., 2003 |
| Anti-HBc prevalence at age 30 | 79.2% | Hipgrave et al., 2003 |

**Model Validation**

1. Comparisons with the actual serosurvey

**Figure 2.2.2S Comparison between 2019 modeled prevalence with the reported 2019 data from Komada et al. by age**


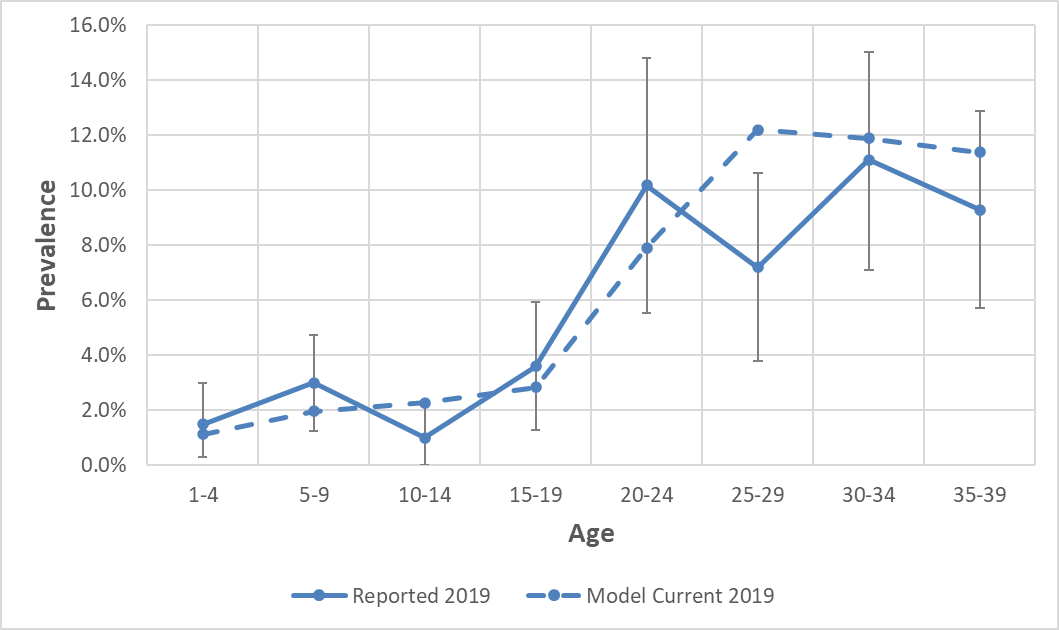


1. Comparisons with Wong et al, 2019

**Figure 2.2.3S Comparison between age-specific modeled prevalence with the overall prevalence in immigrants as estimated by Wong et al., 2019 (not stratified by age)**


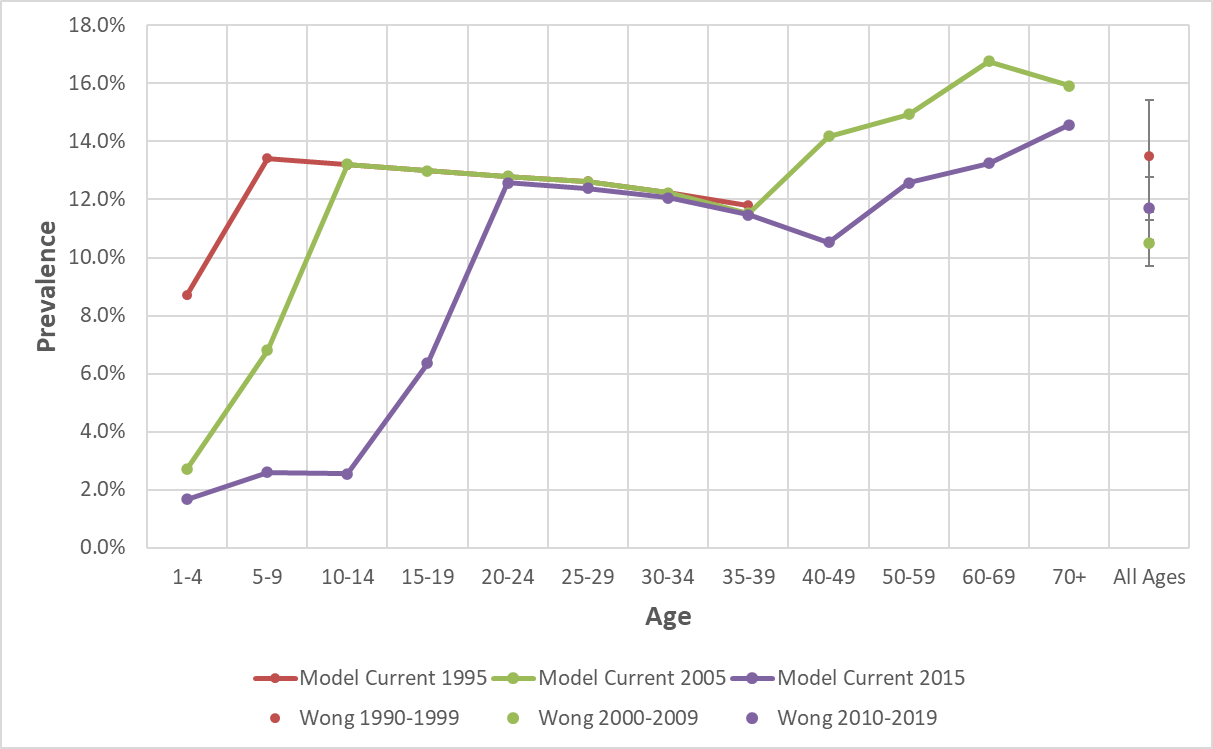


* Model Current 1995 was plotted ranging from age 1 to 39.

**Results**

**Figure 2.2.4S Estimated prevalence under the current, baseline, and WHO scenarios for a 5-year-old in Vietnam**

**
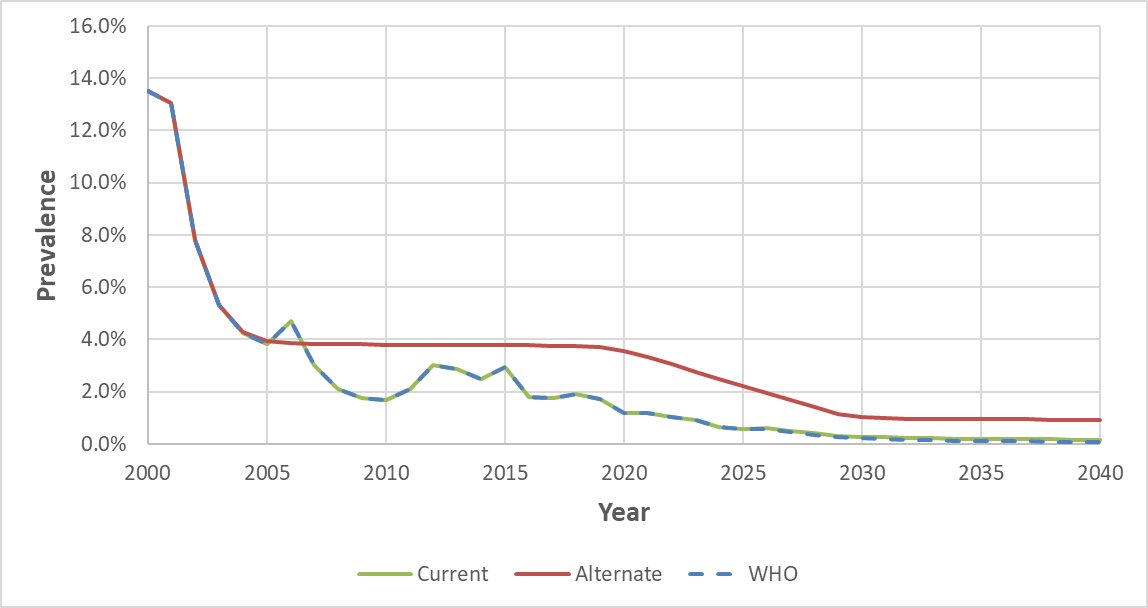
**

**Figure 2.2.5S Estimated prevalence under the current, baseline, and WHO scenarios for a 20-year-old in Vietnam**


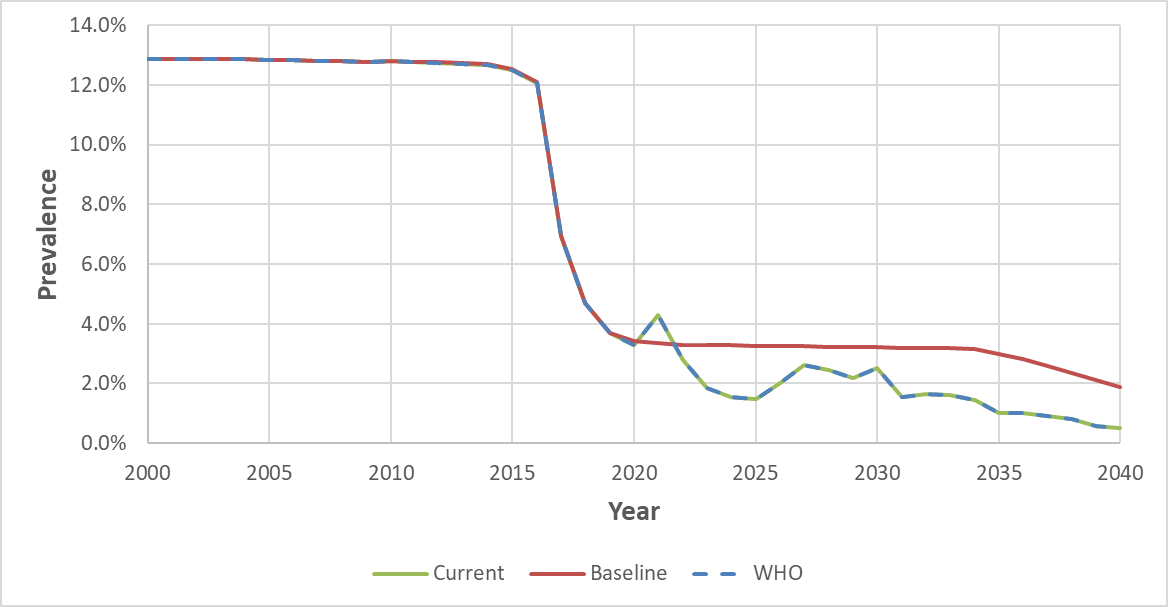


**Figure 2.2.6S Estimates of immigrants with chronic hepatitis B under the current, baseline, and WHO scenarios in Vietnam**


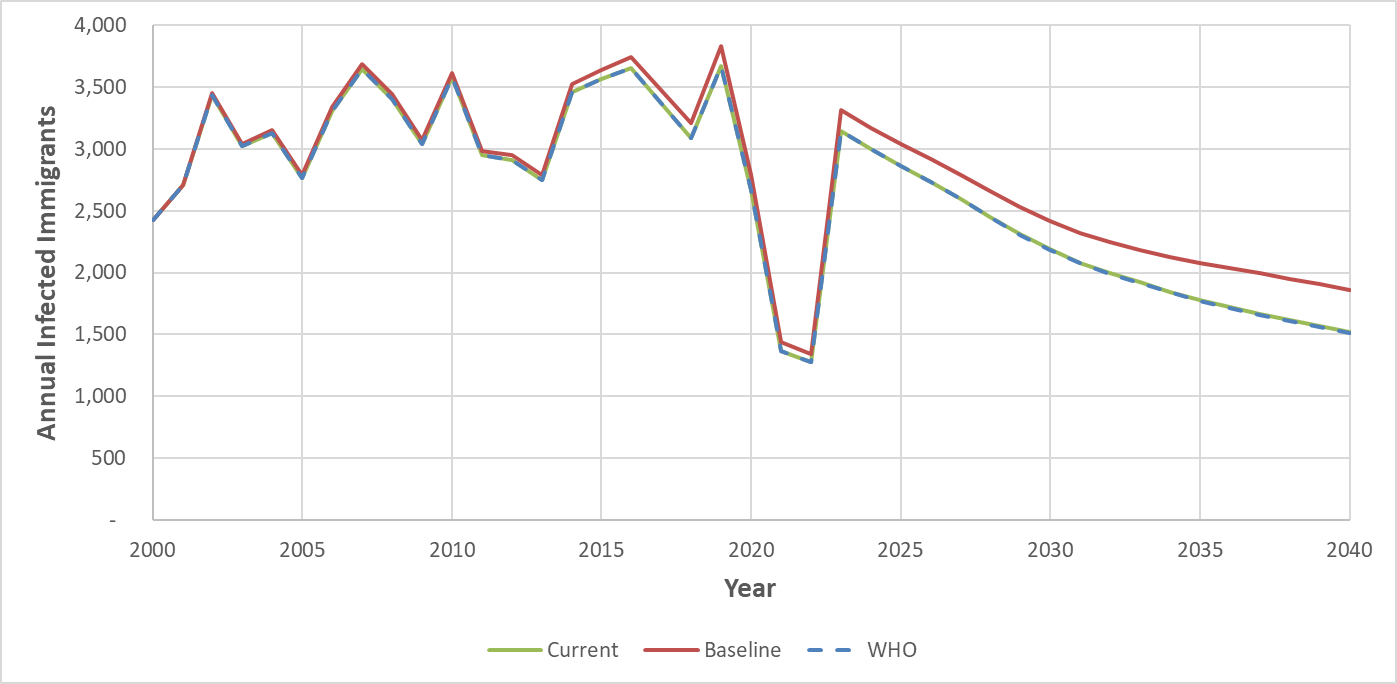


**References:**

Komada K, Hachiya M, Ichimura Y, Shimada M. The impact of hepatitis B vaccination program in central Vietnam; evaluation by population-based, cross-sectional seroprevalence survey of HBsAg. International Journal of Infectious Diseases. 2020 Dec 1;101:477.

Hoang GN, Erickson RV. Cultural barriers to effective medical care among Indochinese patients. Annual Review of Medicine. 1985 Feb;36(1):229-39.

Hipgrave DB, Van NT, Huong VM, Long HT, DO TUAN DA, Trung TN, Jolley D, Maynard JE, Biggs BA. Hepatitis B infection in rural Vietnam and the implications for a national program of infant immunization. The American journal of tropical medicine and hygiene. 2003 Sep 1;69(3):288-94.

**2.3 Philippines**

**Model Set-up**

In the Philippines, the 3-dose HBV vaccination was introduced in 1995, while it was expanded to the timely birth dose in 2007. WHO/UNICEF estimates could cover every year from the introduction of each policy. However, it shows that these policies are not effective enough, as in the Philippines, HepB coverage was as low as 7% in 2000 and peaked at 89% in 2012, as well as timely birth dose coverage was as low as 9% in 2007 and peaked at 60% in 2019. (Figure 2.3.1S)

The HBV prevalence model is developed to estimate the number of HBV-infected Filipino who immigrated to the United States after 2000. To adjust our model, we used a study performed by Lingao et al. studying in 2,842 Philippine rural subjects from four villages in 1979-1982. In 2013, Wong et al. obtained age-specific HBsAg prevalence among people from age groups yielding from 20-29 to over 70 years old by analyzing the 2003 National Nutrition Survey (NNS). In this study, the modeled results will be used to compare with the results from the serosurvey from Wong’s study for validation. (Table 2.3.1S)

For the model of the Philippines, a serosurvey including 5,684 pregnant women performed by Sy et al. between June 1982 and October 1983 was collected. This study covered HBsAg prevalence among pregnant women (7.6%) in Manila, Philippines. HBeAg prevalence among all HBsAg-positive subjects (27.8%) was also derived from the study. The study was chosen because it was the earlier study that covered pregnant women to calculate maternal HBsAg and HBeAg prevalence. A study conducted between 1981 and 1983 by Lingao et al. using data collected from a Hospital in Manila, Philippines. Compared to the study that we adapted, it did not include HBeAg prevalence. Therefore, to make our parameters consistent, we chose study of Sy et al. prior to which of Lingao et al. No literature was found researching the age-specific Anti-HBc prevalence in the Philippines, so indexes estimated by Goldstein et al. (Anti-HBc at 5: 25%; Anti-HBc at 30: 77.5%) were applied in our model for the Philippines. (Table 2.3.2S)

**Vaccination Coverage**

**Figure 2.3.1S Comparison between vaccination coverage under the current, baseline, and WHO scenarios**


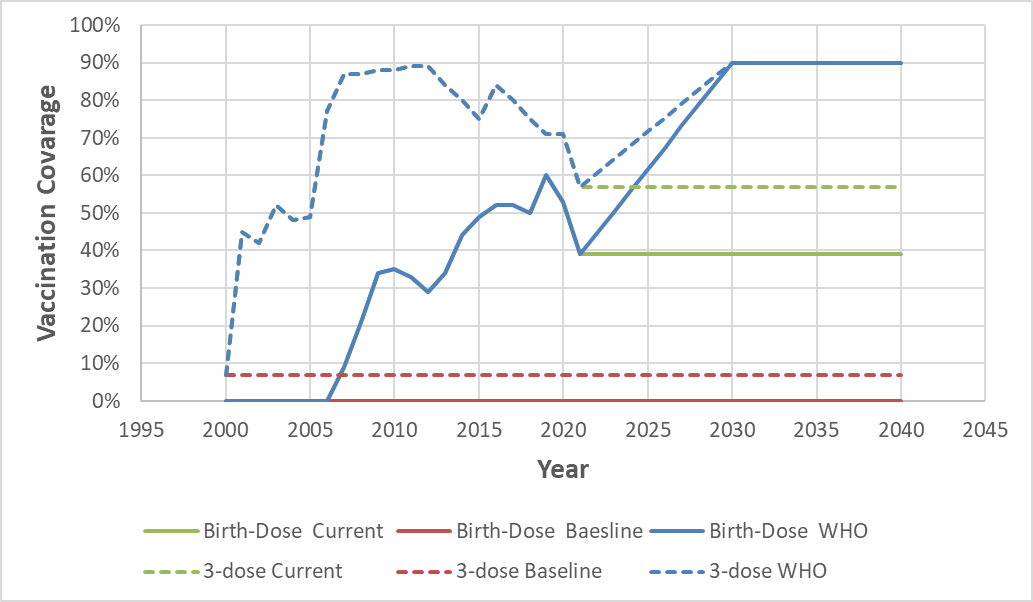


**Table 2.3.1S Summary of actual serosurvey data in Philippines for comparison**

| **Age Group** | **N** | **HBsAg** | **Lower 95% CI** | **Upper 95% CI** | **Survey year** | **Source** |
| --- | --- | --- | --- | --- | --- | --- |
| 20-29 | 329 | 18.10% | 13.60% | 22.60% | 2003 | Wong et al, 2013 |
| 30-39 | 314 | 17.60% | 13.20% | 22.00% |  |  |
| 40-49 | 252 | 16.00% | 11.20% | 20.90% |  |  |
| 50-59 | 162 | 14.30% | 9.00% | 19.50% |  |  |
| 60-69 | 638 | 14.30% | 11.40% | 17.20% |  |  |
| 70+ | 455 | 13.60% | 10.00% | 17.30% |  |  |

**Table 2.3.2S Parameters for Model Philippines**

| **Parameter** | **Value** | **Source** |
| --- | --- | --- |
| Maternal HBsAg prevalence | 7.6% | Sy et al., 1986 |
| Maternal HBeAg prevalence | 27.8% | Sy et al., 1986 |
| Anti-HBc prevalence at age 5 | 25.0% | Goldstein et al., 2005 |
| Anti-HBc prevalence at age 30 | 77.5% | Goldstein et al., 2005 |

**Model Validation**

1. Comparisons with the actual serosurvey

**Figure 2.2.2S Comparison between 2003 modeled prevalence with the reported 2003 data from Wong et al. by age**


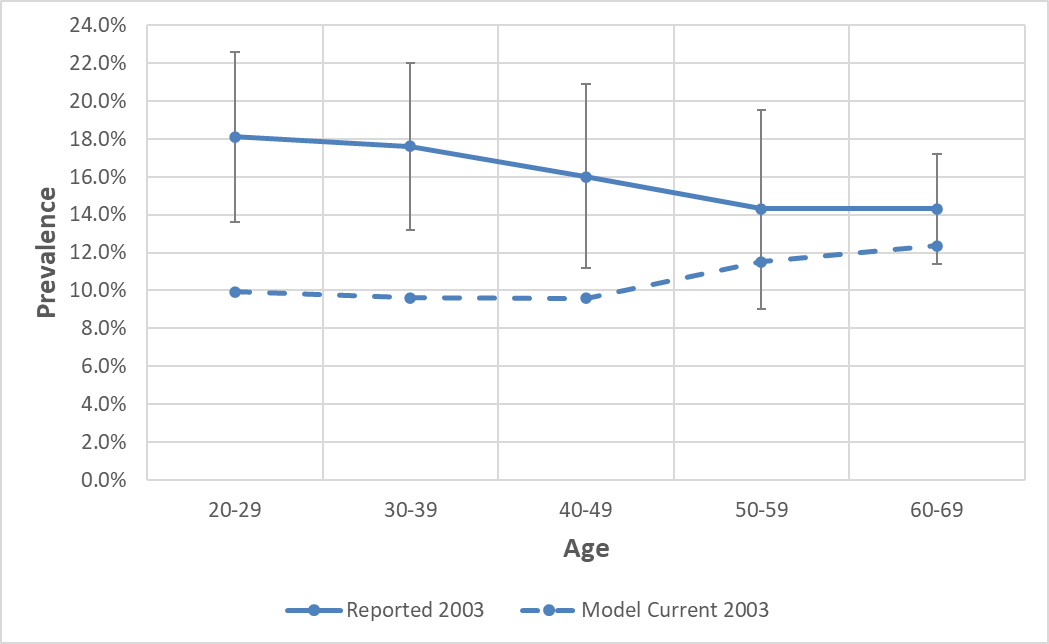


1. Comparisons with Wong et al, 2019

**Figure 2.3.3S Comparison between age-specific modeled prevalence with the overall prevalence in immigrants as estimated by Wong et al., 2019 (not stratified by age)**


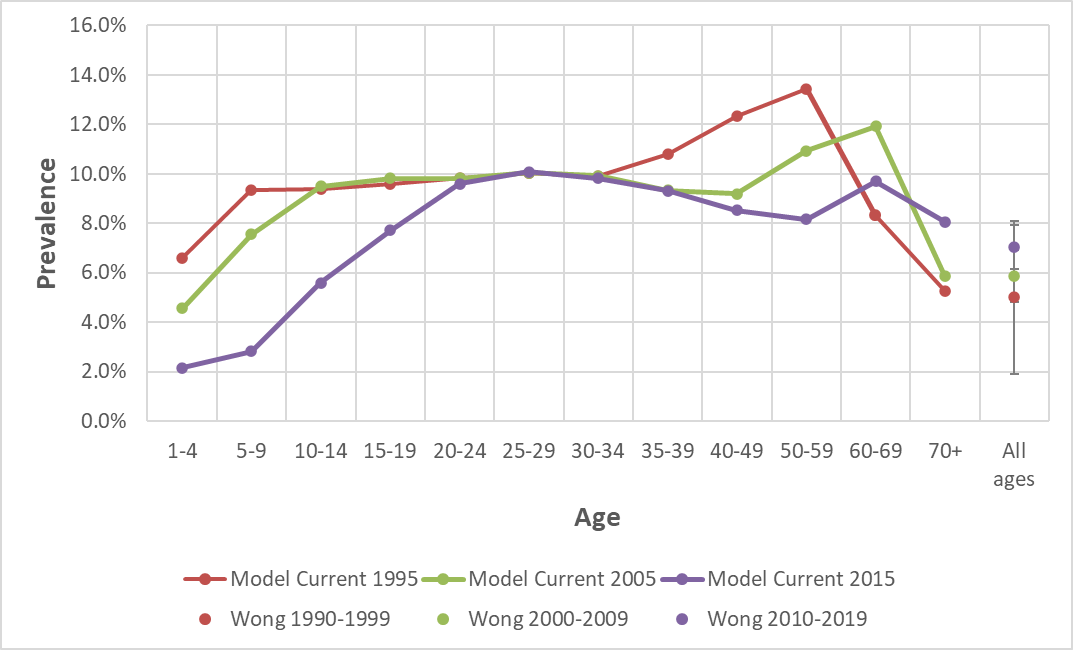


**Results**

**Figure 2.3.4S Estimated prevalence under the current, baseline, and WHO scenarios for a 5-year-old in Philippines**

**
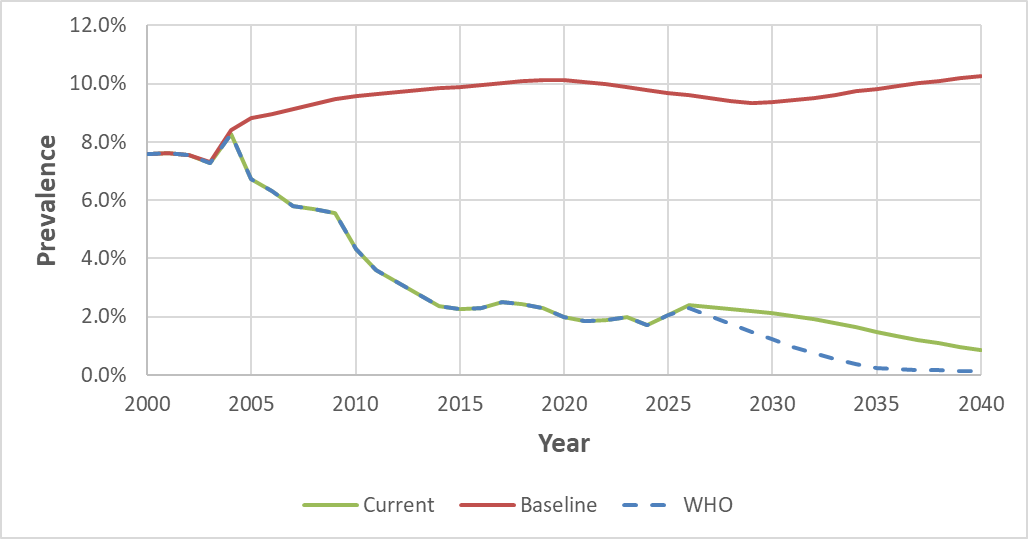
**

**Figure 2.3.5S Estimated prevalence under the current, baseline, and WHO scenarios for a 20-year-old in Philippines**


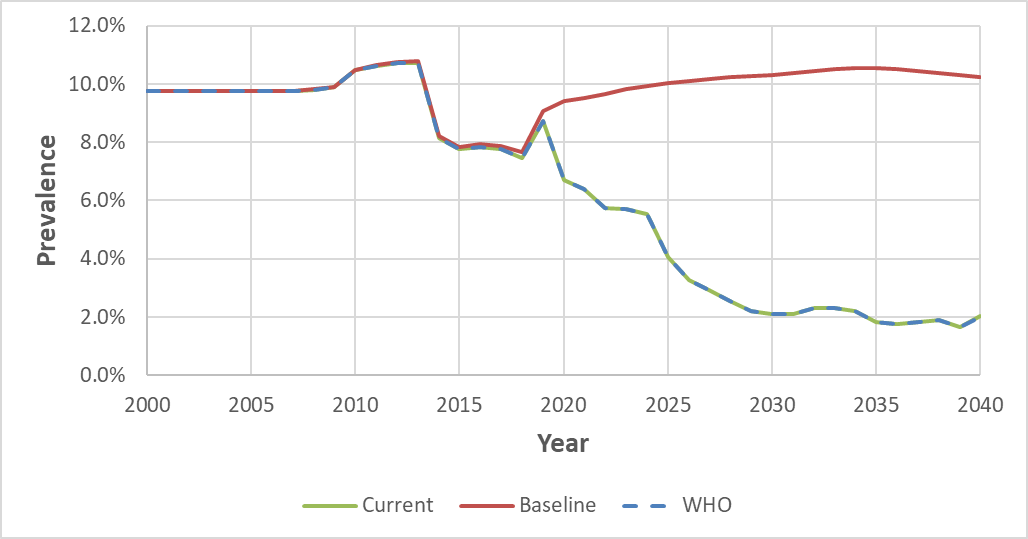


**Figure 2.3.6S Estimates of immigrants with chronic hepatitis B under the current, baseline, and WHO scenarios in Philippines**


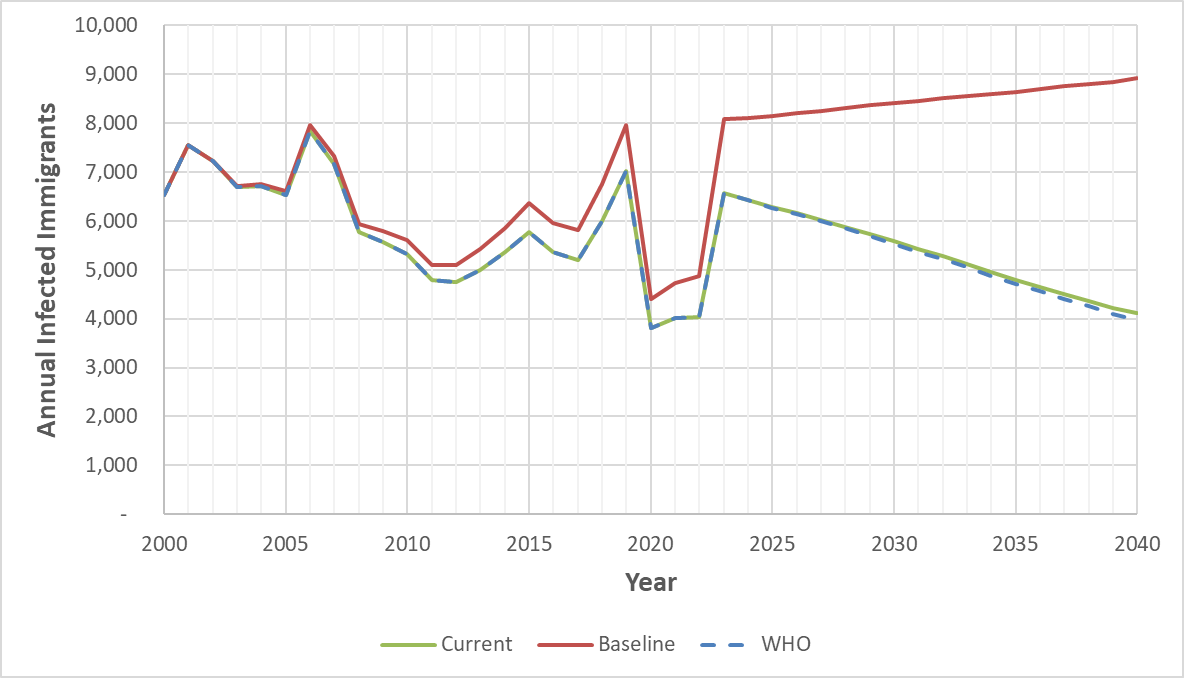


**References:**

Lingao AL, Domingo EO, West S, Reyes CM, Gasmen S, Viterbo G, Tiu E, Lansang MA. Seroepidemiology of hepatitis B virus in the Philippines. American journal of epidemiology. 1986 Mar 1;123(3):473-80.

Lingao AL, Torres NT, Munoz N, Lansang MA, West SK, Bosch FX, Domingo EO. Mother to child transmission of hepatitis B virus in the Philippines. Infection. 1989 Sep 1;17(5):275-9.

Wong SN, Ong JP, Labio ME, Cabahug OT, Daez ML, Valdellon EV, Sollano Jr JD, Arguillas MO. Hepatitis B infection among adults in the philippines: A national seroprevalence study. World journal of hepatology. 2013 Apr 4;5(4):214.

Sy NE, Basaca-Sevilla V, Esguerra T, Beasley RP, Hwang LY, Cross JH. HbsAG and HbeAG markers among pregnant women in Manila, Philippines. Transactions of the Royal Society of Tropical Medicine and Hygiene. 1986 Jan 1;80(5):767-70.

Goldstein ST, Zhou F, Hadler SC, Bell BP, Mast EE, Margolis HS. A mathematical model to estimate global hepatitis B disease burden and vaccination impact. International journal of epidemiology. 2005 Dec 1;34(6):1329-39.

**2.4 India**

**Model Set-up**

In India, the 3-dose HBV vaccination was introduced in 2004, while it was expanded to the timely birth dose in 2008. WHO/UNICEF estimates could cover every year from the introduction of each policy. However, it shows that the policy for timely birth dose is not effective enough, as in India, HepB timely birth dose coverage was as low as 8% in 2008 and peaked at 56% in 2019. (Figure 2.4.1S)

A WHO collaborative study conducted in 1980 evaluated the prevalence of markers of HBV infection in various sites, including Poona, India. Data from this study was used to adjust our model starting from 1980. A cross-sectional, population based survey was conducted by Shadaker et al. from October 2013 to April 2014 in Punjab, India. 5,543 subjects were included in the study, with their age ranging from 5-18 to over 60 years old, and we compared the results from this study to our simulated outcomes for model validation. (Table 2.4.1S)

A study conducted among 8,431 pregnant women in three hospitals in Delhi between February 1986 and October 1989 showed the maternal prevalence of HBsAg was 2.6%. Within the HBsAg positive population, 12.5% of the subjects were HBeAg positive. As the earliest study with the largest study cohort, this study was chosen for the baseline parameters in our model. Anti-HBc positive rates among people at age 5 and age 30 were adapted from Shadaker et al. The study published in 2022 revealed that the prevalence of Anti-HBc among age groups 5-18 and 20-39 were, respectively, 3.8% (95%CI: 2.6%, 5.6%) and 12.8% (95%CI: 10.6%, 15.4%). (Table 2.4.2S)

**Vaccination Coverage**

**Figure 2.4.1S Comparison between vaccination coverage under the current, baseline, and WHO scenarios**


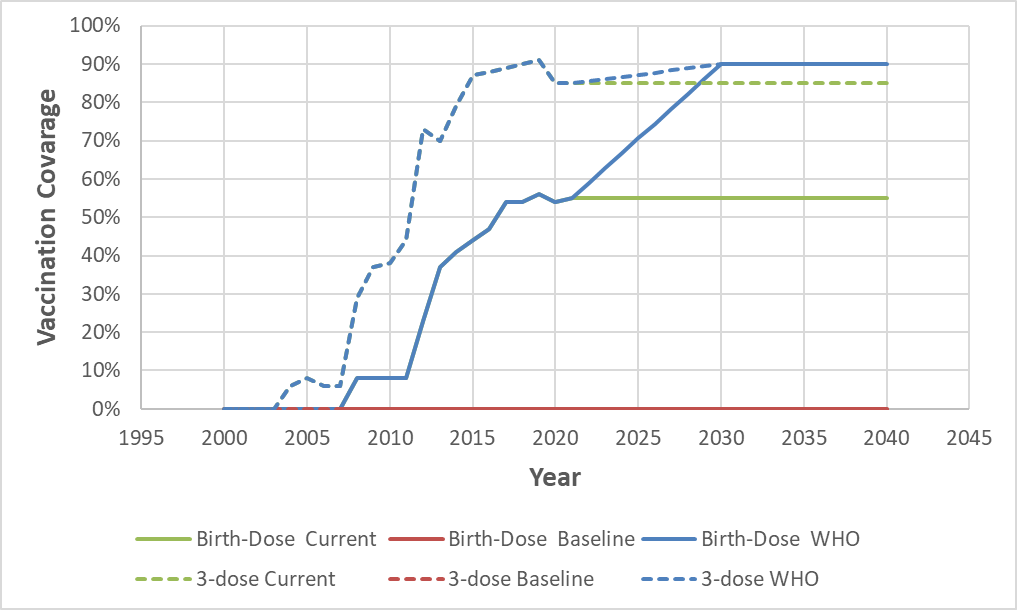


**Table 2.4.1S Summary of actual serosurvey data in India for comparison**

| **Age Group** | **N** | **HBsAg** | **Lower 95% CI** | **Upper 95% CI** | **Survey year** | **Source** |
| --- | --- | --- | --- | --- | --- | --- |
| 5-18 | 1107 | 0.60% | 0.20% | 1.30% | 2014 | Shadaker et al., 2022 |
| 19-29 | 1024 | 1.30% | 0.80% | 2.30% |  |  |
| 30-39 | 998 | 1.10% | 0.60% | 2.20% |  |  |
| 40-49 | 870 | 0.60% | 0.20% | 1.40% |  |  |
| 50-59 | 721 | 2.80% | 1.80% | 4.40% |  |  |
| 60+ | 823 | 2.60% | 1.60% | 4.10% |  |  |

**Table 2.4.2S Parameters for Model India**

| **Parameter** | **Value** | **Source** |
| --- | --- | --- |
| Maternal HBsAg prevalence | 2.60% | Panda et al., 1991 |
| Maternal HBeAg prevalence | 12.5% | Panda et al., 1991 |
| Anti-HBc prevalence at age 5 | 3.8% | Shadaker et al., 2022 |
| Anti-HBc prevalence at age 30 | 12.80% | Shadaker et al., 2022 |

**Model Validation**

1. Comparisons with the actual serosurvey

**Figure 2.4.2S Comparison between 2014 modeled prevalence with the reported 2014 data from Shadaker et al. by age**

**
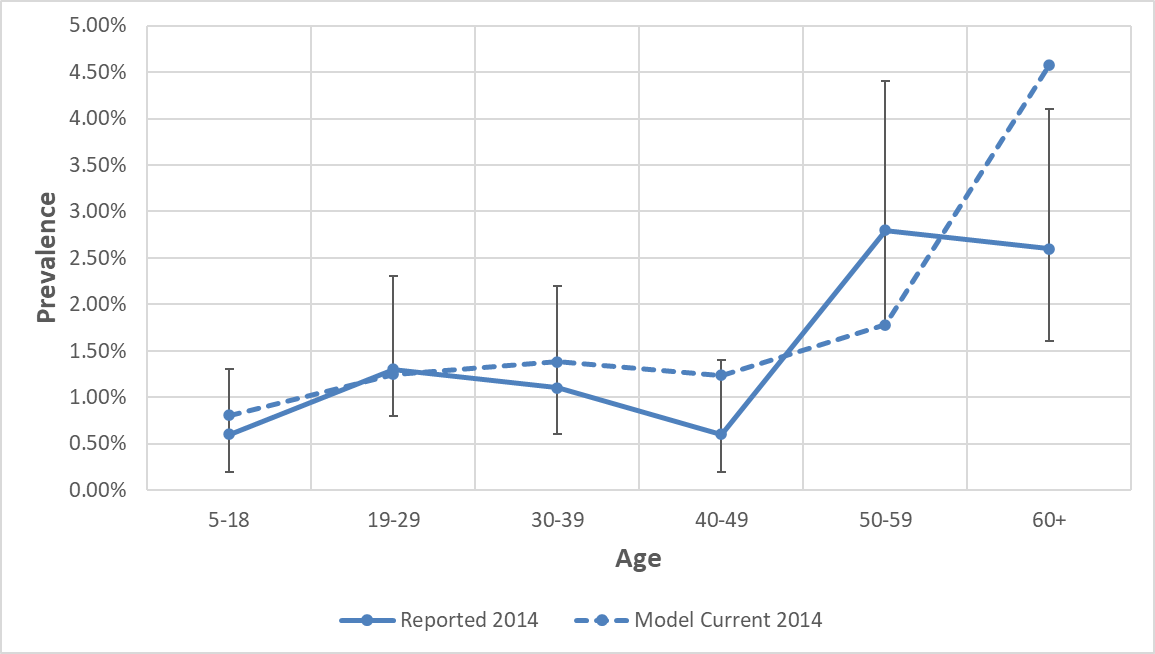
**

1. Comparisons with Wong et al, 2019

**Figure 2.4.3S Comparison between age-specific modeled prevalence with the overall prevalence in immigrants as estimated by Wong et al., 2019 (not stratified by age)**


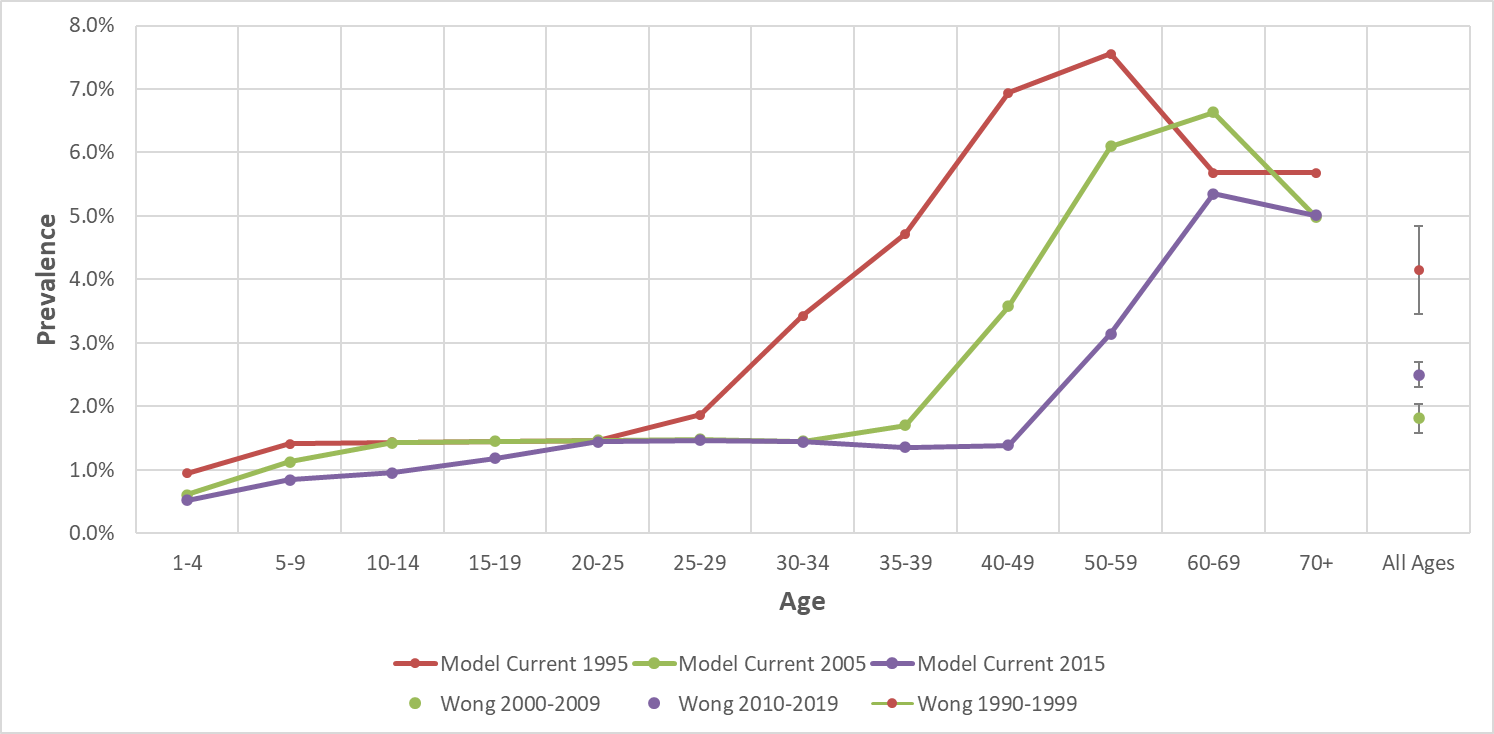


**Results**

**Figure 2.4.4S Estimated prevalence under the current, baseline, and WHO scenarios for a 5-year-old in India**


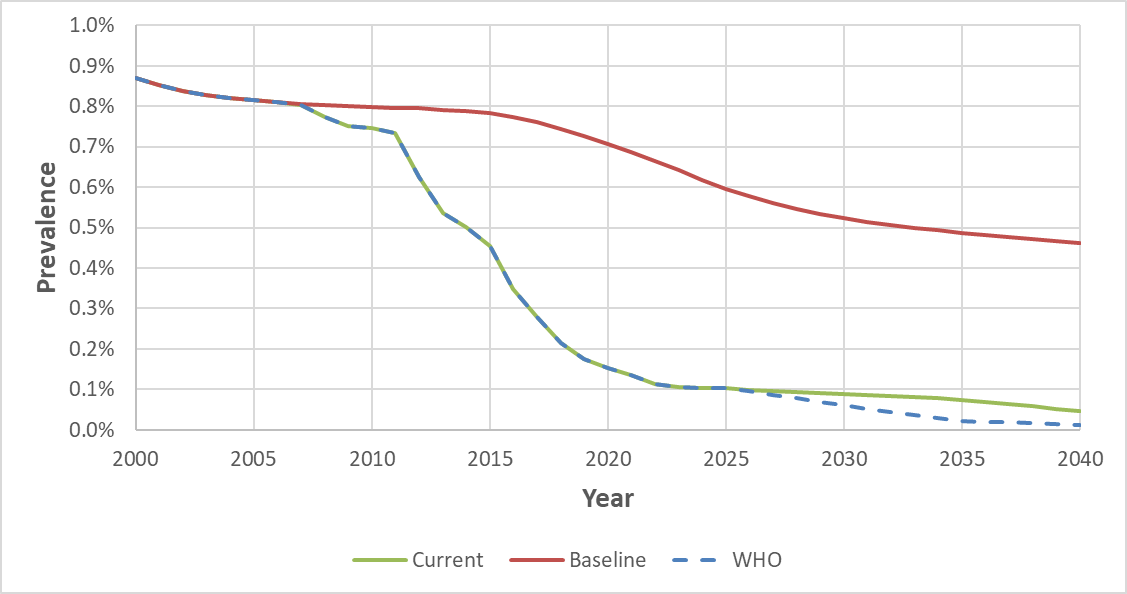


**Figure 2.4.5S Estimated prevalence under the current, baseline, and WHO scenarios for a 20-year-old in India**

**
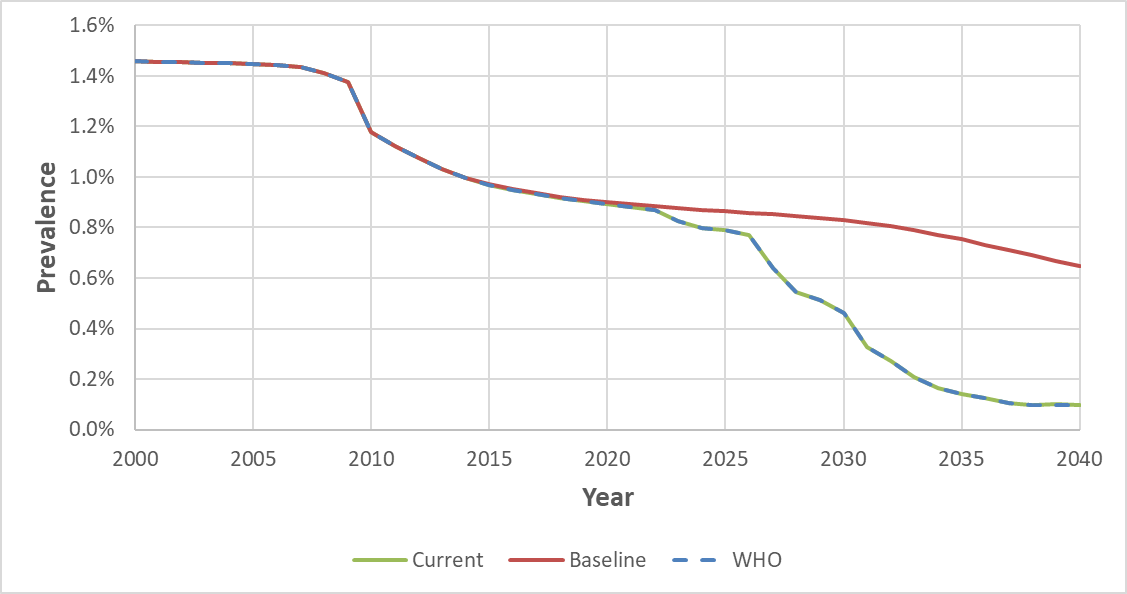
**

**Figure 2.4.6S Estimates of immigrants with chronic hepatitis B under the current, baseline, and WHO scenarios in India**


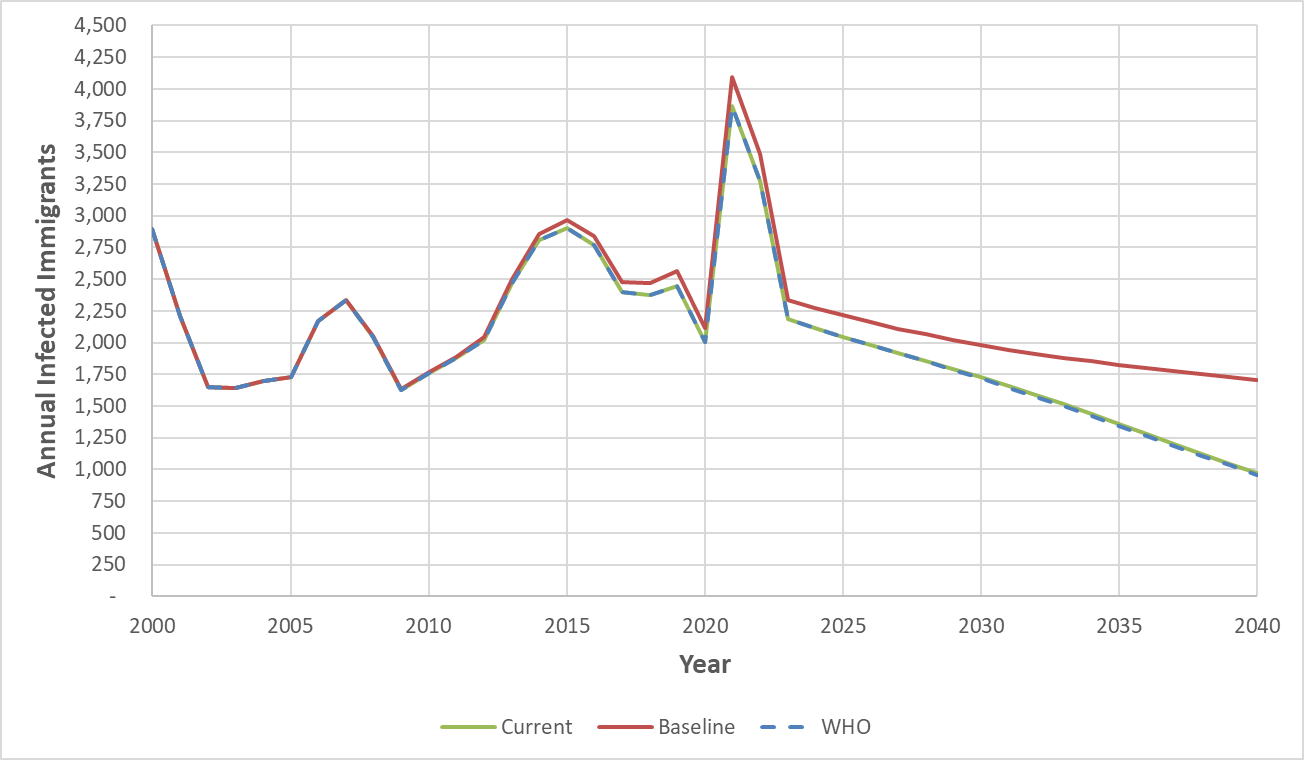


**References:**

Shadaker S, Sood A, Averhoff F, Suryaprasad A, Kanchi S, Midha V, Kamili S, Nasrullah M, Trickey A, Garg R, Mittal P. Hepatitis B Prevalence and Risk Factors in Punjab, India: A Population-Based Serosurvey. Journal of Clinical and Experimental Hepatology. 2022 Sep 1;12(5):1310-9.

Panda SK, Ramesh R, Rao KV, Gupta A, Zuckerman AJ, Nayak NC. Comparative evaluation of the immunogenicity of yeast‐derived (recombinant) and plasma‐derived hepatitis B vaccine in infants. Journal of medical virology. 1991 Dec;35(4):297-302.

**2.5 Dominican Republic**

**Model Set-up**

The Dominican Republic launched its 3-dose HBV (HepB) vaccination program in 1994, and the timely birth dose (TBD) vaccination program in 1997. However, estimates from WHO/UNICEF could only cover the HepB coverage from 1998 and the TBD coverage from 2001. The official reported HepB courage rates were used from 1994 to 1997, but the TBD coverage remained blank from 1997 to 2000. Simulation, in this case, was performed based on available data provided by WHO, and we assumed the TBD coverage rate to be a linear trend from 0 in 1996 to 81% in 2001 in the Dominican Republic. (Figure 2.5.1S)

For model validation, one study informed the actual serosurvey data in the Dominican Republic in 1995 and it was used for model validation. However, due to the high variance in that 1996 study, and given the limited availability of serosurveys for the Dominican Republic, we compared our modeling results with the results of previous models by other research groups. (Figure 2.5.2S, Figure 2.5.3S)

Baseline maternal prevalence of HBsAg, anti-HBc at 5 years old, and anti-HBc at 30 years old used for the Dominican Republic model was obtained from a study published by Silveira et al. This study published in 1991 observed a total of 12,000 subjects from Latin America with 473 subjects from the Dominican Republic between June 1996 and November 1997. In this study, the anti-HBc prevalence in age groups 1-5 and 31-40 were recorded as 9.9% (95%CI: 2.9%, 16.7%) and 37.9% (95%CI: 27.7%, 48.1%), respectively, and the HBsAg prevalence was detected as 1.9%. This prevalence of HBsAg might not be maternal, but this was the only real-world survey-based prevalence data that could be found aside from the study by Silveira et al. that contained age-specified information. As no literature support was found for the maternal prevalence of HBeAg, an estimated parameter of 15% was picked from the Goldstein model. (Table 2.5.2S)

**Vaccination Coverage**

**Figure 2.5.1S Comparison between vaccination coverage under the current, baseline, and WHO scenarios**


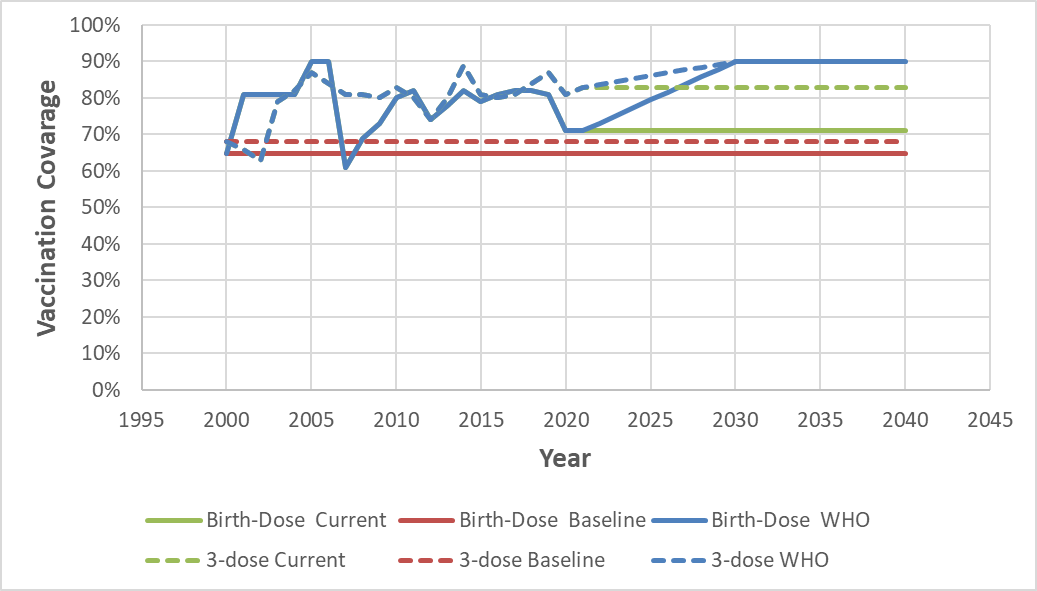


**Table 2.5.1S Summary of actual serosurvey data in Dominican Republic for comparison**

| **Age Group** | **N** | **HBsAg** | **Lower 95% CI** | **Upper 95% CI** | **Survey year** | **Source** |
| --- | --- | --- | --- | --- | --- | --- |
| 20-29 | 683 | 2.34% | -5.86% | 10.54% | 1995 | Shichijo et al, 1996 |
| 30-39 | 556 | 3.42% | -4.00% | 10.84% |  |  |
| 40-49 | 365 | 4.38% | -4.78% | 13.55% |  |  |
| 50-59 | 216 | 2.78% | -6.69% | 12.25% |  |  |
| 60-69 | 123 | 1.63% | -1.69% | 4.94% |  |  |
| 70+ | 57 | 7.02% | -8.24% | 22.27% |  |  |

**Table 2.5.2S Parameters for Model Dominican Republic**

| **Parameter** | **Value** | **Source** |
| --- | --- | --- |
| Maternal HBsAg prevalence | 1.90% | Silveira et al., 1999 |
| Maternal HBeAg prevalence | 15.0% | Goldstein et al., 2005 |
| Anti-HBc prevalence at age 5 | 9.90% | Silveira et al., 1999 |
| Anti-HBc prevalence at age 30 | 37.90% | Silveira et al., 1999 |

**Model Validation**

1. Comparisons with the actual serosurvey

**Figure 2.5.2S Comparison between modeled prevalence with the reported data by age**

**
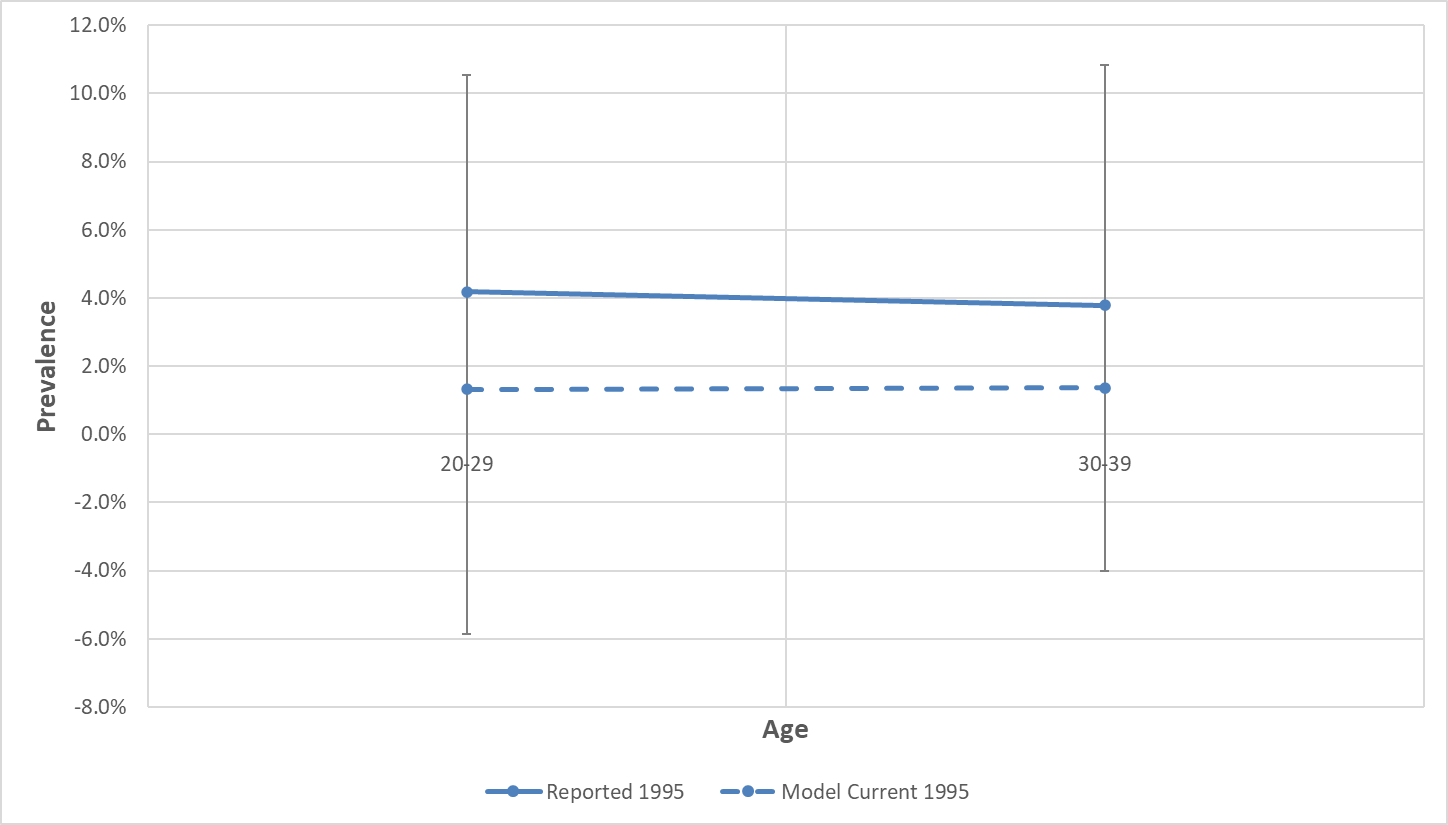
**

**Figure 2.5.3S Comparison between modeled prevalence with the reported data by year among children under 5 years olds**


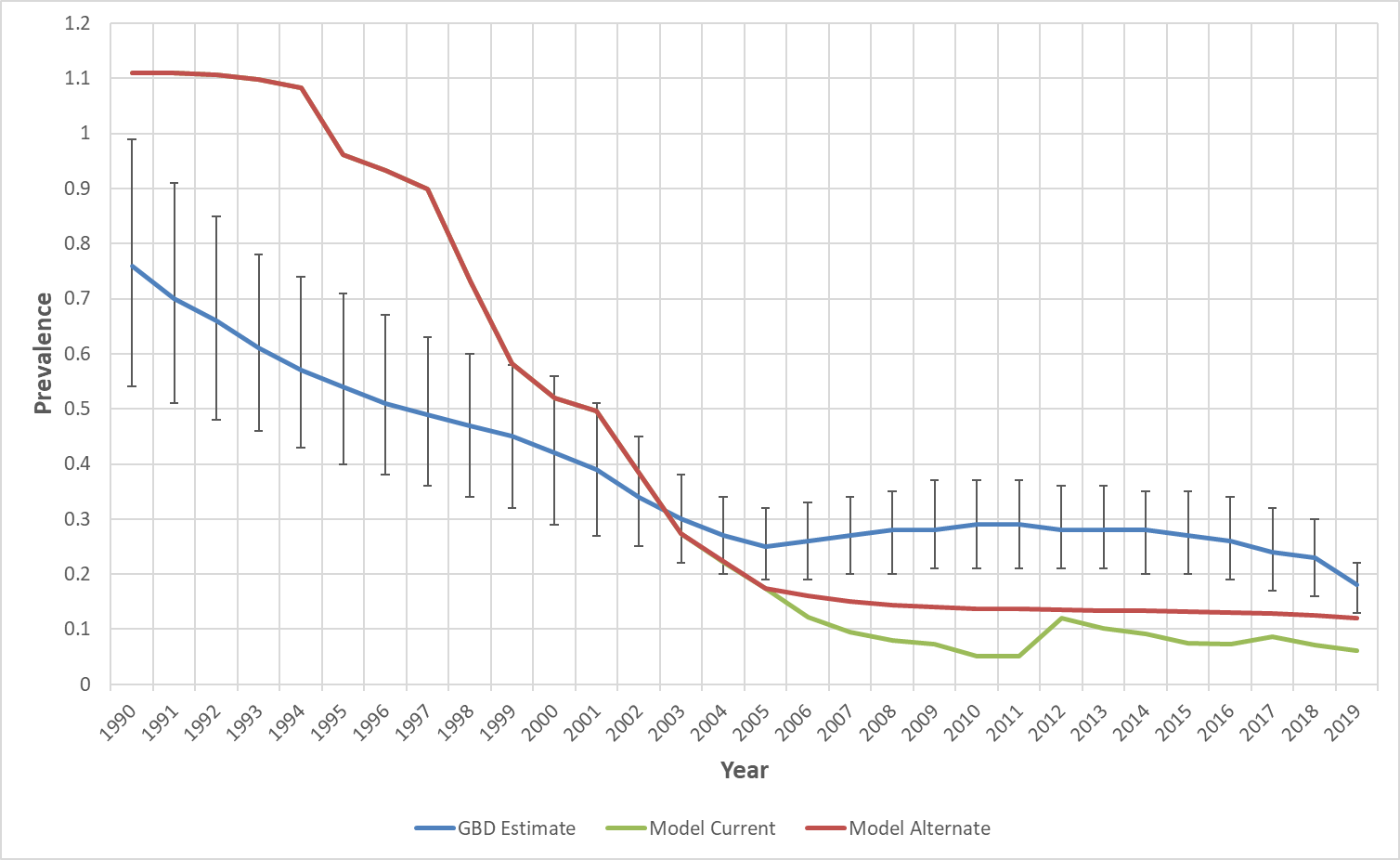


1. Comparisons with Wong et al, 2019

**Figure 2.5.4S Comparison between age-specific modeled prevalence with the overall prevalence in immigrants as estimated by Wong et al., 2019 (not stratified by age)**


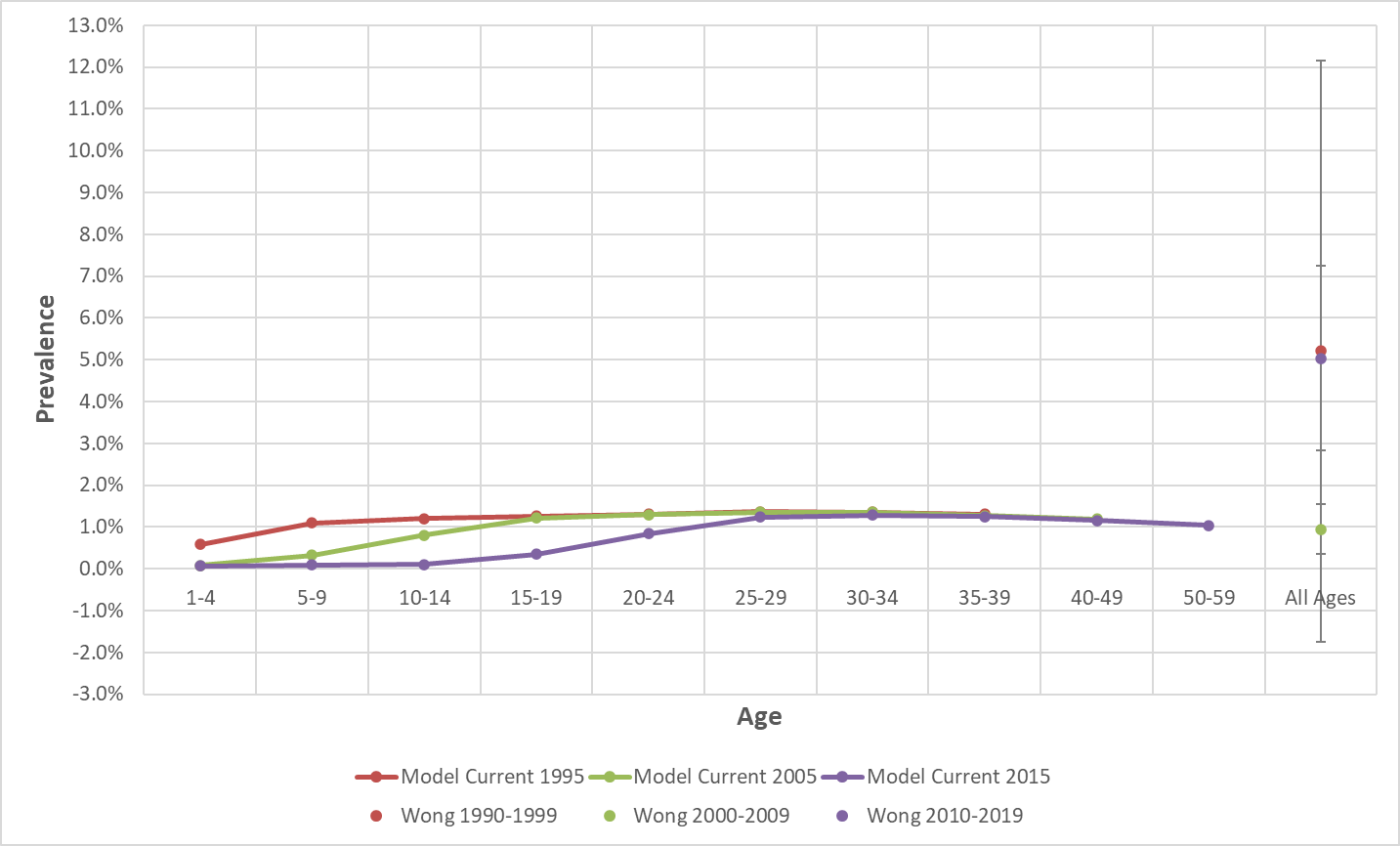


* Model Current 1995 was plotted ranging from age 1 to 39; Model Current 2005 was plotted ranging from age 1 to 49.

**Results**

**Figure 2.5.5S Estimated prevalence under the current, baseline, and WHO scenarios for a 5-year-old in Dominican Republic**

**
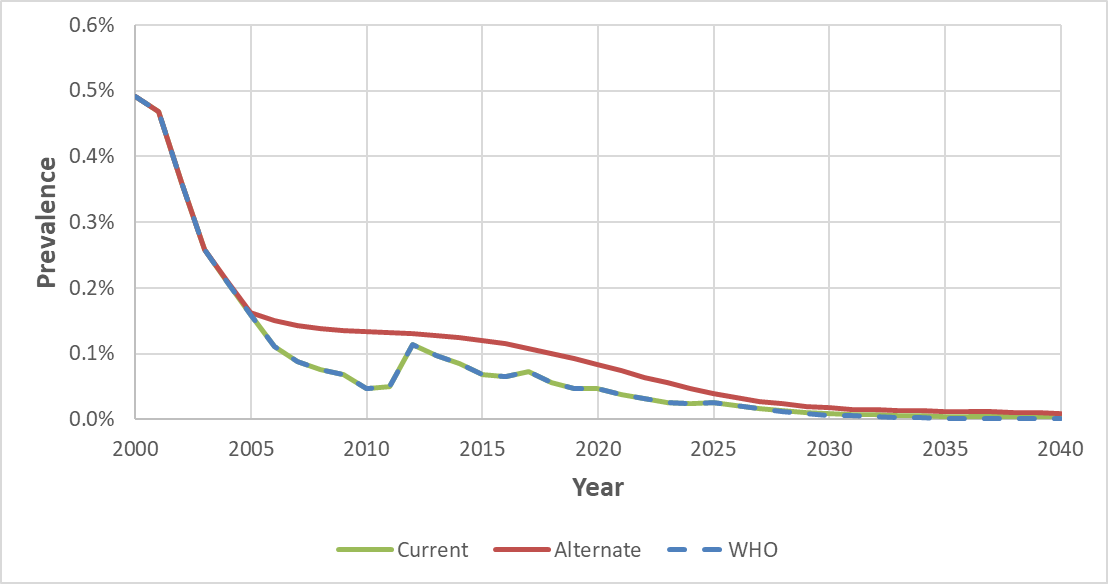
**

**Figure 2.5.6S Estimated prevalence under the current, baseline, and WHO scenarios for a 20-year-old in Dominican Republic**


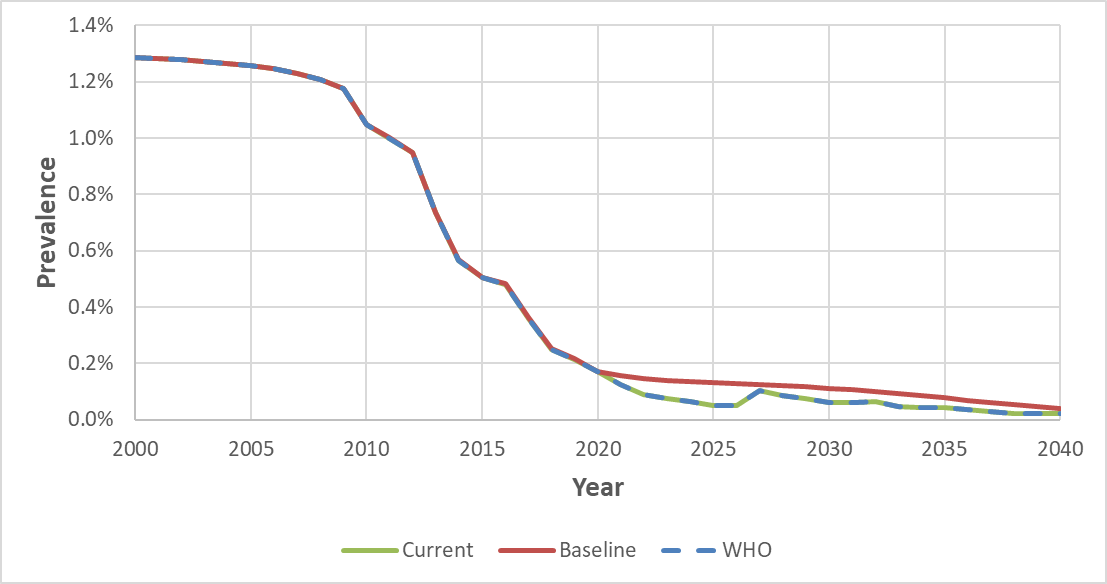


**Figure 2.5.7S Estimates of immigrants with chronic hepatitis B under the current, baseline, and WHO scenarios in Dominican Republic**


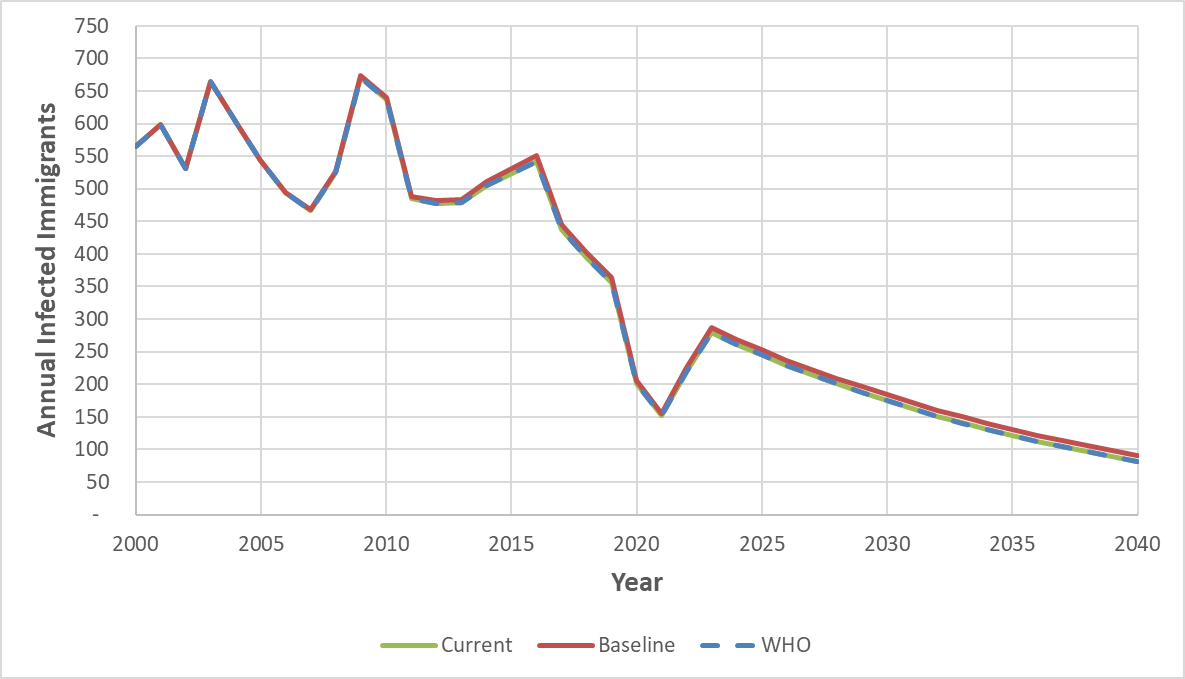


**References:**

**Model Set-up**

Shichijo A, Mifune K, Terao H, Rikihisa T, Itoga T, Norman Me, Rodriguez Ag, Bello Mc, Fernandez Fs. Seroepidemiological Studies of Hepatitis Viruses in the Dominican Republic II. The Prevalence of Hepatitis D and E Virus Infections. Japanese Journal of Tropical Medicine and Hygiene. 1996 Dec 15;24(4):233-6.

Goldstein ST, Zhou F, Hadler SC, Bell BP, Mast EE, Margolis HS. A mathematical model to estimate global hepatitis B disease burden and vaccination impact. International journal of epidemiology. 2005 Dec 1;34(6):1329-39.

Silveira TR, Fonseca JC, Rivera L, Fay OH, Tapia R, Santos JI, Urdeneta E, Clemens SA. Hepatitis B seroprevalence in Latin America. Revista Panamericana de Salud Pública. 1999;6:378-83.

**2.6 Taiwan**

**Model Set-up**

Taiwan introduced hepatitis B immunoglobulin (HBIG) and hepatitis B vaccine to newborns of high-risk (HBsAg-positive) mothers in 1984 and expanded it to all newborns in 1986 (Chien et al., 2006). We adopted vaccination coverage sources different from other country models because WHO/UNICEF does not provide vaccination estimation rates from Taiwan. Su et al. (2019) documented HBIG coverage data among infants of HBeAg-seropositive mothers from 1984 to 2016. Chien et al. (2006) noted the 3-dose hepatitis B vaccination coverage rate among birth cohorts from 1984 to 2002. The Centers for Disease Control (Taiwan) reported the annual 3-dose hepatitis B vaccination data in the "Statistics Communicable Diseases and Surveillance Report" from 2004 to 2020. A linear estimation was applied to fulfill the data gap of 3-dose coverage in 2003. As Taiwan has achieved a high and stable vaccination rate, it is assumed that Taiwan maintained the same HBIG vaccination rate from 2016 to 2021 and the same 3-dose vaccination rate from 2020 to 2021.

The HBV prevalence model is developed to estimate the number of CHB-infected Taiwanese in the US after 2000. We initiated the Taiwan model in 1954, 30 years before the vaccination program. Infants only at age 0 were estimated for the HBV prevalence in the model's first year, and the estimation expanded to broader age groups each year. The model calculated HBsAg prevalence among maternal age groups (21 to 30 years old) in 1984 and provided the HBV prevalence estimation among most high-risk groups (20 to 40 years old) in 2000.

The modeled HBV prevalence rate is compared with actual serosurveys to make adjustments. Researchers performed epidemiologic HBV seromarker surveys in Taipei city in 1984, 1989, 1994, 1999, 2004, 2009, 2014, and 2019, calculating the HBsAg of the birth cohort before and after the universal infant HBV vaccination program (Hsu et al., 1986; Tsen et al., 1991; Chen et al.,1996; Ni et al., 2001; Ni et al., 2007; Ni et al., 2012; Ni et al., 2016; Chang et al., 2022). We compared model results with serosurveys every ten years after 1984.

After comparing and optimizing model inputs, the final parameters we used for maternal Maternal HBsAg prevalence, Maternal HBeAg prevalence, and Anti-HBc prevalence at ages 5 and 30 are listed in Table 2.6.1S. We adopted maternal HBsAg data from 1984 to 2000 from Su et al. (2019), which examined 32 years of cross-sectional data on a maternal HBsAg and HBeAg screening program launched in July 1984. Chen et al. (2011) reported that the HBeAg seropositivity was in 41.26% of HBV carriers in the birth cohort of 1977. Anti-HBc prevalence rates are from Hsu et al. (1986) age group 5-6 years old and Goldstein et al. (2005) age group 30.

**Vaccination Coverage**

**Figure 2.6.1S. Comparison between vaccination coverage under the baseline and current vaccination coverage scenarios**


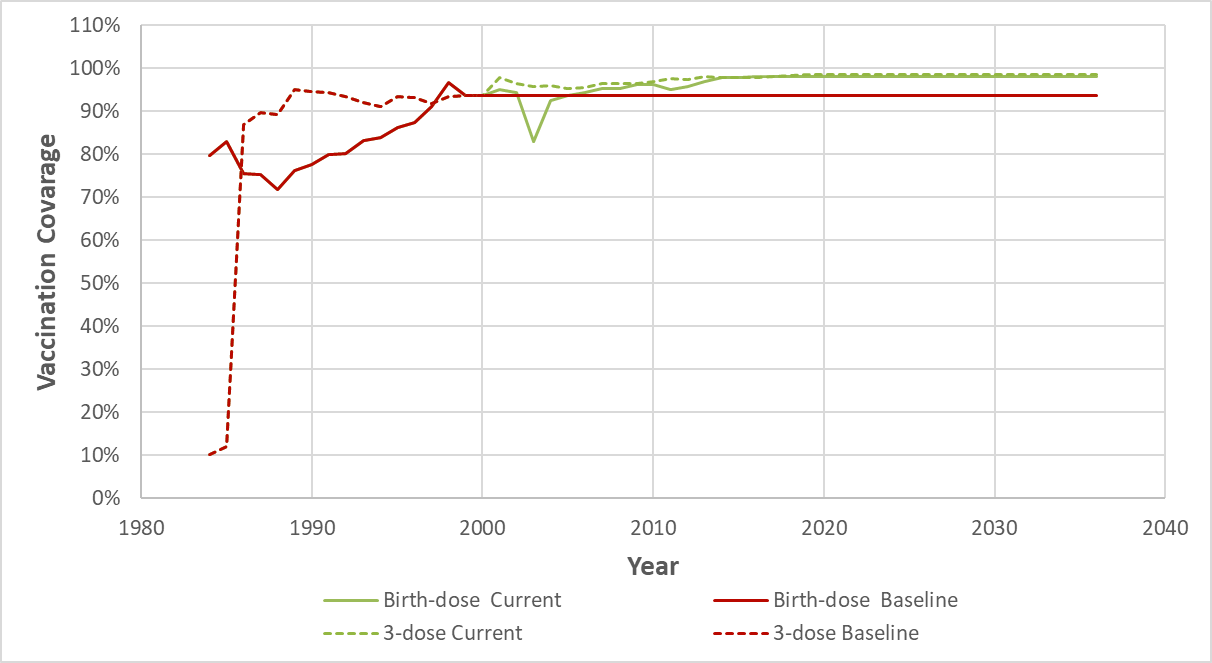


* WHO scenario not shown since Taiwan has exceeded WHO vaccination goals

| **Table 2.6.1S. Parameters for Model Taiwan** | | |
| --- | --- | --- |
| **Parameter** | **Value** | **Source** |
| Maternal HBsAg prevalence | 16.30% | Su et al., 2019 |
| Maternal HBeAg prevalence | 41.26% | Chen et al., 2011 |
| Anti-HBc prevalence at age 5 | 22.10% | Hsu et al., 1986 |
| Anti-HBc prevalence at age 30 | 77.50% | Goldstein et al., 2005 |

| **Table 2.6.2S. Summary of actual serosurvey data in Taiwan for comparison** | | | | | | |
| --- | --- | --- | --- | --- | --- | --- |
| **Age Group** | **N** | **HBsAg** | **Lower 95% CI** | **Upper 95% CI** | **Survey year** | **Source** |
| -1 | 59 | 5.1% | 1.1% | 14.1% | 1984 | Hsu et al., 1986 |
| 1-2 | 84 | 10.7% | 5.0% | 19.4% |  |  |
| 3-4 | 189 | 10.1% | 6.2% | 15.2% |  |  |
| 5-6 | 226 | 10.6% | 6.9% | 15.4% |  |  |
| 7-8 | 206 | 9.7% | 6.0% | 14.3% |  |  |
| 9-10 | 146 | 11.0% | 6.4% | 17.2% |  |  |
| 11-12 | 187 | 9.1% | 5.4% | 14.2% |  |  |
| 13-14 | 103 | 11.7% | 6.2% | 19.5% |  |  |
| -1 | 20 | 0.0% | 0.0% | 16.8% | 1994 | Chen et al., 1996 |
| 1-2 | 211 | 0.5% | 0.0% | 2.6% |  |  |
| 3-4 | 313 | 0.3% | 0.0% | 1.8% |  |  |
| 5-6 | 371 | 0.8% | 0.2% | 2.3% |  |  |
| 7-8 | 218 | 0.9% | 1.1% | 3.3% |  |  |
| 9-10 | 206 | 1.5% | 0.3% | 4.2% |  |  |
| 11-12 | 146 | 6.8% | 3.3% | 12.2% |  |  |
| -1 | 110 | 0.9% | 0.7% | 1.1% | 2004 | Ni et al., 2007 |
| 1-2 | 235 | 0.4% | 0.4% | 0.5% |  |  |
| 3-4 | 709 | 0.4% | 0.4% | 0.4% |  |  |
| 5-6 | 993 | 0.5% | 0.5% | 0.5% |  |  |
| 7-8 | 651 | 0.6% | 0.6% | 0.6% |  |  |
| 9-10 | 681 | 0.3% | 0.3% | 0.3% |  |  |
| 11-12 | 1088 | 0.5% | 0.5% | 0.5% |  |  |
| 13-14 | 2767 | 0.6% | 0.6% | 0.6% |  |  |
| 15-17 | 6531 | 1.5% | 1.5% | 1.5% |  |  |
| 18-19 | 3872 | 2.1% | 2.1% | 2.1% |  |  |
| 20-21 | 346 | 6.7% | 6.5% | 6.8% |  |  |
| 22-23 | 256 | 10.2% | 9.9% | 10.4% |  |  |
| 24-25 | 242 | 10.3% | 10.0% | 10.6% |  |  |
| 26-27 | 184 | 17.9% | 17.5% | 18.3% |  |  |
| 28-29 | 114 | 14.9% | 14.3% | 15.5% |  |  |
| -1 | 106 | 0.9% | 0.8% | 1.1% | 2014 | Ni et al., 2016 |
| 1-2 | 181 | 0.0% | 0.0% | 0.0% |  |  |
| 3-4 | 198 | 0.0% | 0.0% | 0.0% |  |  |
| 5-6 | 207 | 0.5% | 0.4% | 0.6% |  |  |
| 7-8 | 227 | 0.0% | 0.0% | 0.0% |  |  |
| 9-10 | 212 | 0.9% | 0.9% | 1.0% |  |  |
| 11-12 | 236 | 0.4% | 0.4% | 0.5% |  |  |
| 13-14 | 202 | 0.0% | 0.0% | 0.0% |  |  |
| 15-16 | 247 | 0.0% | 0.0% | 0.0% |  |  |
| 17-18 | 237 | 1.3% | 1.2% | 1.4% |  |  |
| 19-20 | 247 | 0.8% | 0.7% | 0.9% |  |  |
| 21-22 | 221 | 0.4% | 0.4% | 0.5% |  |  |
| 23-24 | 268 | 0.4% | 0.3% | 0.4% |  |  |
| 25-26 | 223 | 0.4% | 0.4% | 0.5% |  |  |
| 27-28 | 199 | 1.0% | 0.9% | 1.1% |  |  |
| 29 | 88 | 2.3% | 1.9% | 2.6% |  |  |
| 30 | 103 | 3.9% | 3.5% | 4.3% |  |  |
| 31-32 | 177 | 7.3% | 7.1% | 7.6% |  |  |
| 33-34 | 171 | 3.5% | 3.3% | 3.7% |  |  |
| 35-36 | 163 | 5.5% | 5.3% | 5.8% |  |  |
| 37-38 | 135 | 6.7% | 6.3% | 7.0% |  |  |
| 39-40 | 128 | 9.4% | 8.9% | 9.8% |  |  |
| 41-42 | 89 | 3.4% | 3.0% | 3.8% |  |  |
| 43-44 | 87 | 4.6% | 4.1% | 5.1% |  |  |
| 45-46 | 83 | 10.8% | 10.1% | 11.6% |  |  |
| 47-48 | 86 | 16.3% | 15.4% | 17.1% |  |  |
| 49-50 | 84 | 6.0% | 5.4% | 6.5% |  |  |

**Model Validation**

1. Comparisons with the serosurvey

**Figure 2.6.2S.** **Comparison between 1984, 1994, 2004 and 2014 modeled prevalence by age with the reported serosurvey data from Hsu et al., 1986; Chen et al.,1996; Ni et al., 2007; Ni et al., 2016**

1. **1984**

**
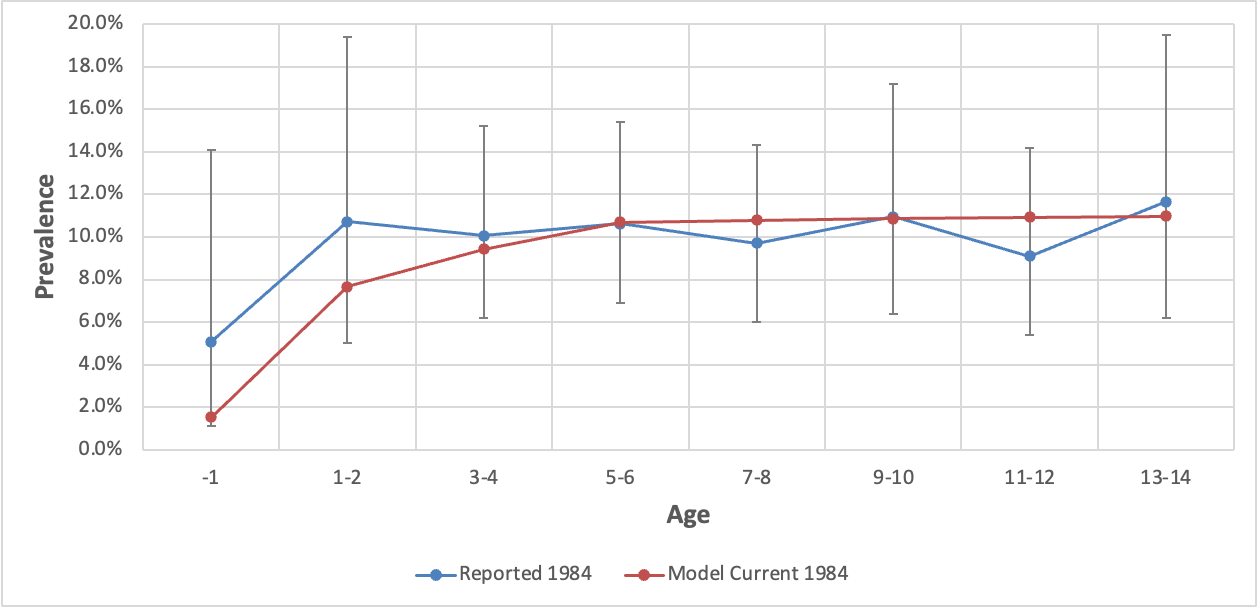
**

1. **1994**

**
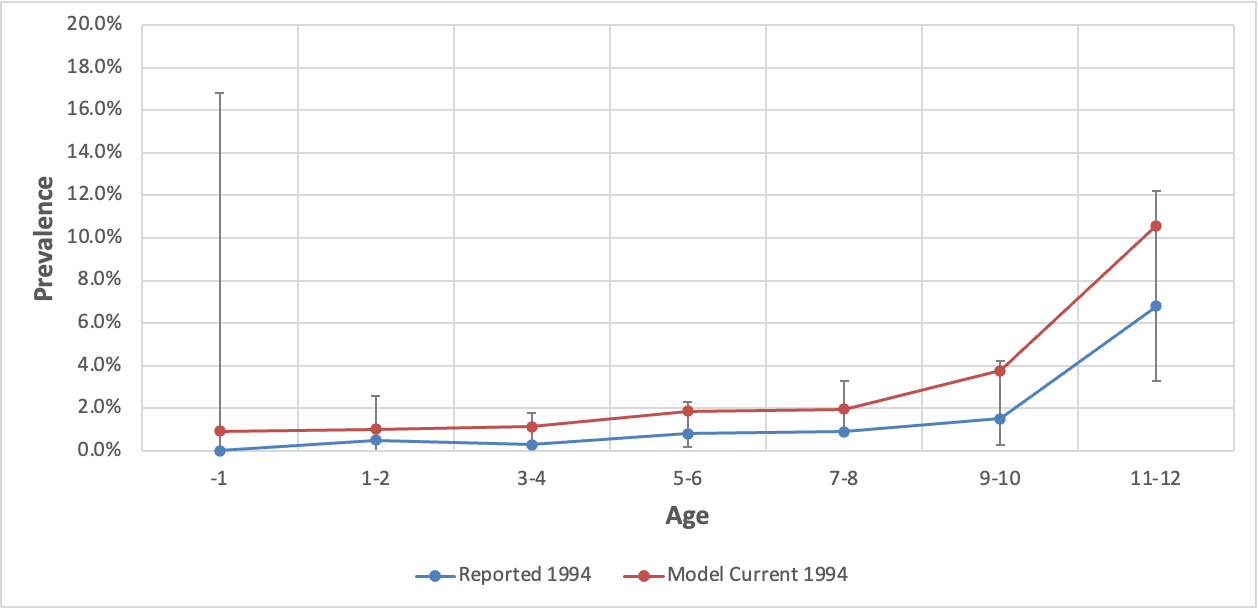
**

1. **2004**

**
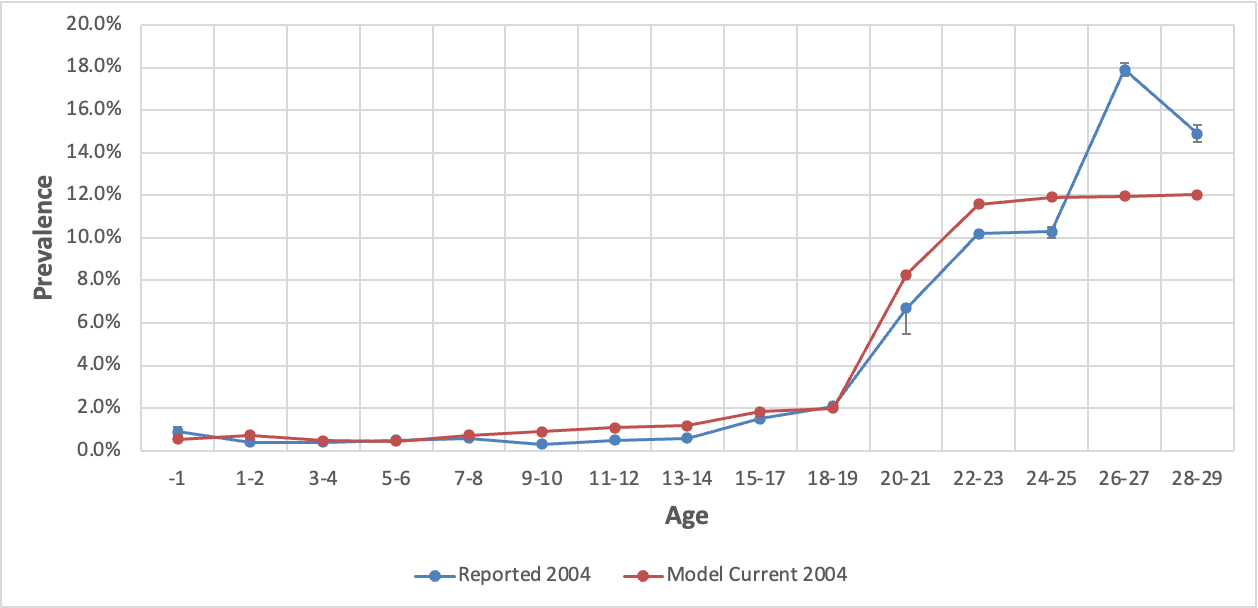
**

1. **2014**

**
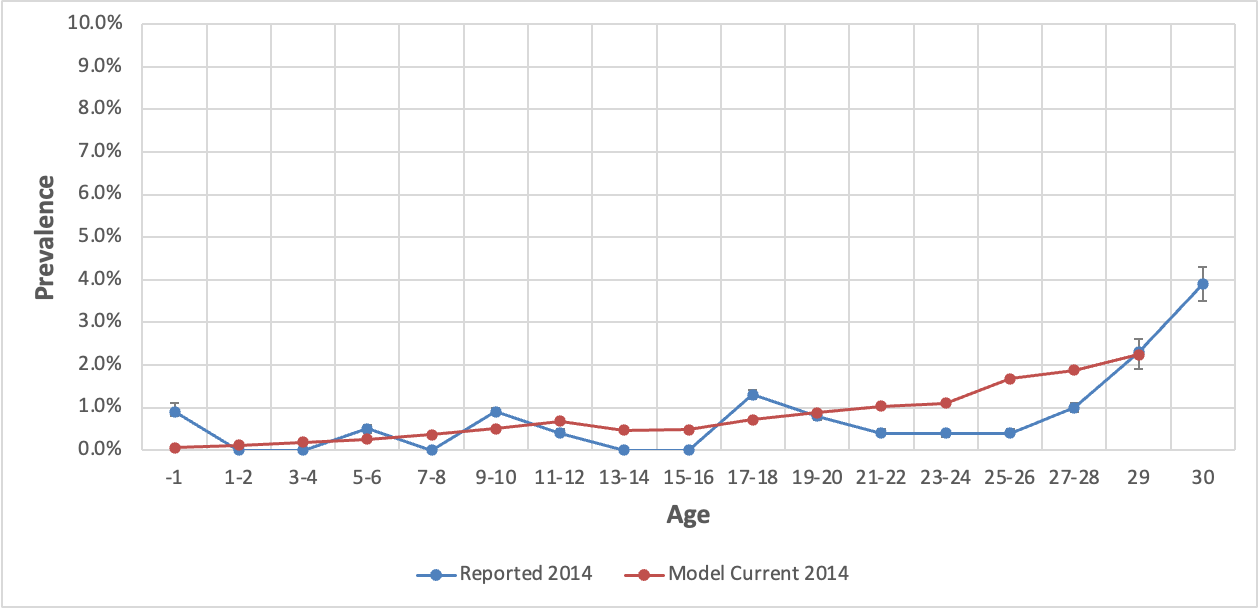
**

1. Comparisons with Wong et al. (2019)

**Figure 2.6.3S Comparison between age-specific modeled prevalence with the overall prevalence in immigrants as estimated by Wong et al., 2019 (not stratified by age)**


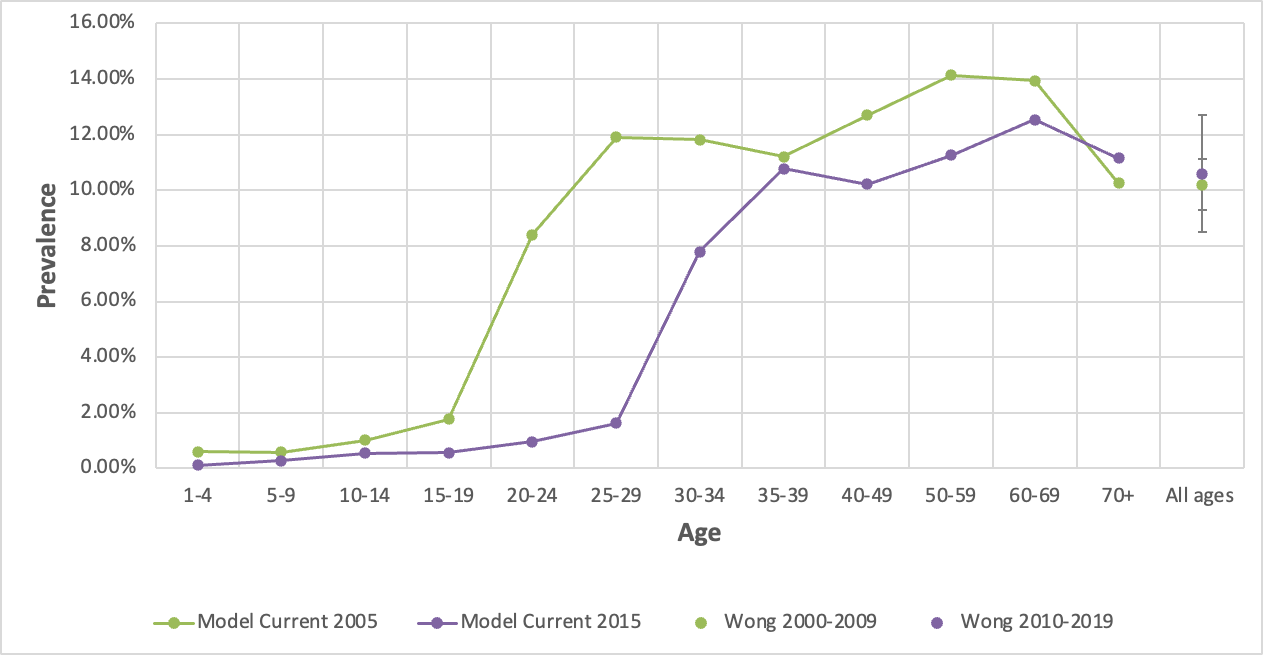


**Results**

**Figure 2.6.4.1S Estimation of prevalence under the baseline and current vaccination coverage scenarios for a 5-year-old Taiwanese**


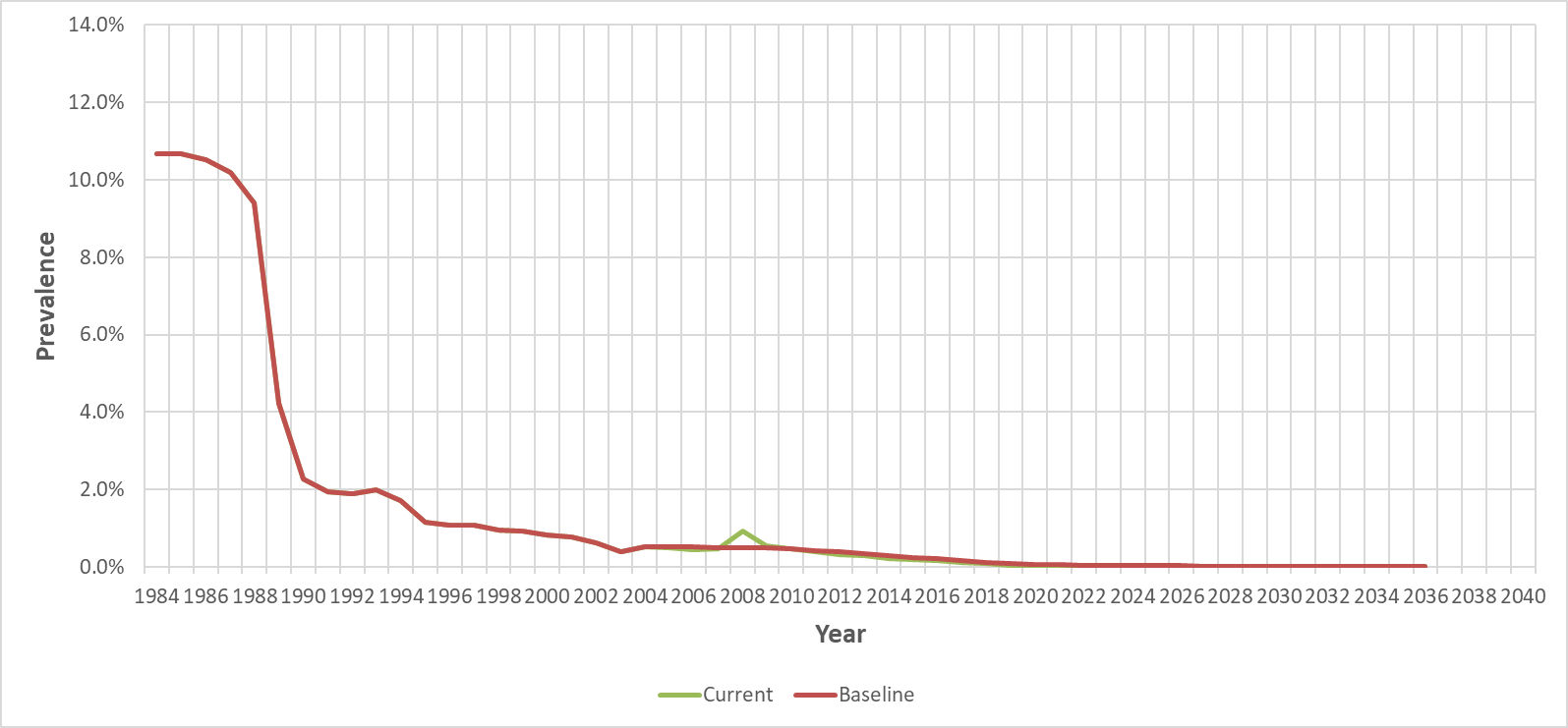


* WHO scenario not shown since Taiwan has exceeded WHO vaccination goals

**Figure 2.6.4.2S Estimation of prevalence under the baseline and current vaccination coverage scenarios for a 20-year-old Taiwanese**


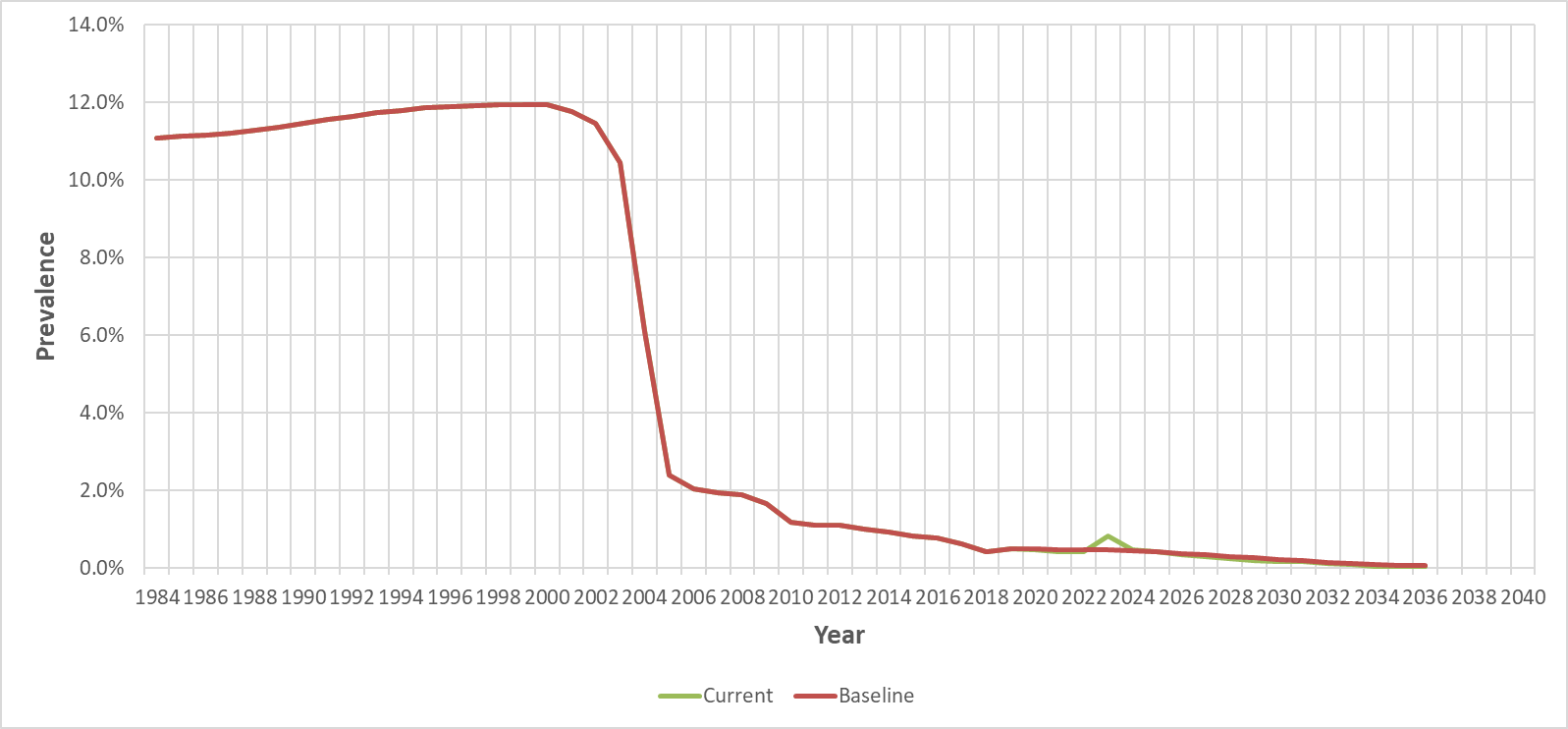


* WHO scenario not shown since Taiwan has exceeded WHO vaccination goals

**Figure 2.6.5S Estimation of immigrants with chronic hepatitis B under the baseline and current vaccination coverage scenarios in Taiwan.**


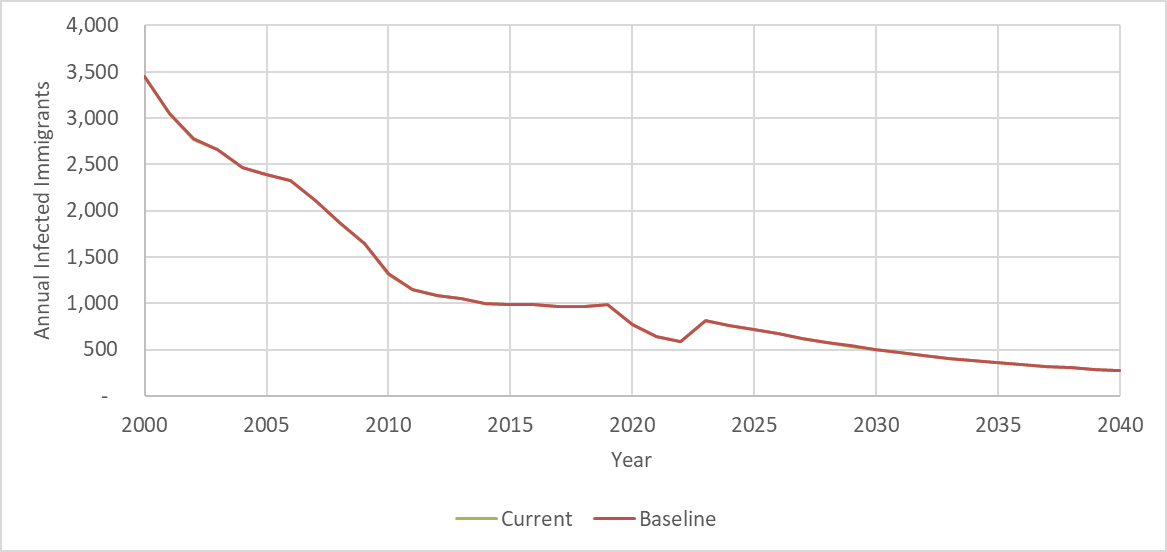


* Baseline overlaps Current and WHO scenario not shown since Taiwan has exceeded WHO vaccination goals

**References:**

Chien YC, Jan CF, Kuo HS, Chen CJ. Nationwide hepatitis B vaccination program in Taiwan: effectiveness in the 20 years after it was launched. Epidemiol Rev. 2006;28:126-35. doi: 10.1093/epirev/mxj010. Epub 2006 Jun 16. PMID: 16782778.

Centers for Disease Control (Taiwan). Statistics Communicable Diseases and Surveillance Report. [Internet]. 2004-2020. [cited 2022 Sep 11]. Available from: <https://www.cdc.gov.tw/InfectionReport/List/DRiONFTwYxu8T162Hm6yFw>

Su, W. J., Chen, S. F., Yang, C. H., Chuang, P. H., Chang, H. F., & Chang, M. H. (2019). The impact of universal infant hepatitis B immunization on reducing the hepatitis B carrier rate in pregnant women. *The Journal of Infectious Diseases*, *220*(7), 1118-1126.

Hsu HY, Chang MH, Chen DS, Lee CY, Sung JL. Baseline seroepidemiology of hepatitis B virus infection in children in Taipei, 1984: a study just before mass hepatitis B vaccination program in Taiwan. J Med Virol. 1986 Apr;18(4):301-7. doi: 10.1002/jmv.1890180402. PMID: 2940332.

Tsen YJ, Chang MH, Hsu HY, Lee CY, Sung JL, Chen DS. Seroprevalence of hepatitis B virus infection in children in Taipei, 1989: five years after a mass hepatitis B vaccination program. J Med Virol. 1991 Jun;34(2):96-9. doi: 10.1002/jmv.1890340205. PMID: 1832440.

Chen HL, Chang MH, Ni YH, Hsu HY, Lee PI, Lee CY, Chen DS. Seroepidemiology of hepatitis B virus infection in children: Ten years of mass vaccination in Taiwan. JAMA. 1996 Sep 18;276(11):906-8. PMID: 8782640.

Ni YH, Chang MH, Huang LM, Chen HL, Hsu HY, Chiu TY, Tsai KS, Chen DS. Hepatitis B virus infection in children and adolescents in a hyperendemic area: 15 years after mass hepatitis B vaccination. Ann Intern Med. 2001 Nov 6;135(9):796-800. doi: 10.7326/0003-4819-135-9-200111060-00009. PMID: 11694104.

Ni YH, Huang LM, Chang MH, Yen CJ, Lu CY, You SL, Kao JH, Lin YC, Chen HL, Hsu HY, Chen DS. Two decades of universal hepatitis B vaccination in taiwan: impact and implication for future strategies. Gastroenterology. 2007 Apr;132(4):1287-93. doi: 10.1053/j.gastro.2007.02.055. Epub 2007 Feb 25. PMID: 17433322.

Ni YH, Chang MH, Wu JF, Hsu HY, Chen HL, Chen DS. Minimization of hepatitis B infection by a 25-year universal vaccination program. J Hepatol. 2012 Oct;57(4):730-5. doi: 10.1016/j.jhep.2012.05.021. Epub 2012 Jun 2. PMID: 22668640.

Ni YH, Chang MH, Jan CF, Hsu HY, Chen HL, Wu JF, Chen DS. Continuing Decrease in Hepatitis B Virus Infection 30 Years After Initiation of Infant Vaccination Program in Taiwan. Clin Gastroenterol Hepatol. 2016 Sep;14(9):1324-30. doi: 10.1016/j.cgh.2016.04.030. Epub 2016 May 4. PMID: 27155556.

Chang KC, Chang MH, Chen HL, Wu JF, Chang CH, Hsu HY, Ni YH. Universal Infant Hepatitis B Virus (HBV) Vaccination for 35 Years: Moving Toward the Eradication of HBV. J Infect Dis. 2022 Feb 1;225(3):431-435. doi: 10.1093/infdis/jiab401. PMID: 34363469.

Su WJ, Chen SF, Yang CH, Chuang PH, Chang HF, Chang MH. The Impact of Universal Infant Hepatitis B Immunization on Reducing the Hepatitis B Carrier Rate in Pregnant Women. J Infect Dis. 2019 Aug 30;220(7):1118-1126. doi: 10.1093/infdis/jiy706. PMID: 30576506.

Chen SM, Kung CM, Yang WJ, Wang HL. Efficacy of the nationwide hepatitis B infant vaccination program in Taiwan. J Clin Virol. 2011 Sep;52(1):11-6. doi: 10.1016/j.jcv.2011.06.012. Epub 2011 Jul 20. PMID: 21767983.

Goldstein ST, Zhou F, Hadler SC, Bell BP, Mast EE, Margolis HS. A mathematical model to estimate global hepatitis B disease burden and vaccination impact. Int J Epidemiol. 2005 Dec;34(6):1329-39. doi: 10.1093/ije/dyi206. Epub 2005 Oct 25. PMID: 16249217.

**2.7 South Korea**

**Model Set-up**

South Korea introduced the hepatitis B vaccine to newborns in 1983 and included the hepatitis B vaccine in the national mandatory vaccine in 1995 (Chen, 2013). We adopted vaccination coverage from WHO/UNICEF estimation since 1995 and literature reviews prior to 1995. WHO/UNICEF estimated birth dose and 3-dose hepatitis B vaccination in South Korea from 2000 to 2020 and 1995 to 2021. Prior to the data recorded by WHO/UNICEF, Lee et al. (1997) counted the 3-dose hepatitis B vaccination rate among 2072 elementary school students born between 1980 and 1987. We applied the linear estimation to fulfill this data gap for the birth dose and 3-dose vaccination rate unreported from 1984 to 2000 and from 1988 to 1994. As South Korea has achieved a high and stable vaccination rate, it is assumed that South Korea will maintain the same birth-dose vaccination rate from 2020 to 2021.

The HBV prevalence model is developed to estimate the number of CHB-infected South Koreans in the US after 2000. We initiated the South Korea model in 1954, 30 years before the vaccine was valid in South Korea. Infants only at age 0 were estimated for the HBV prevalence in the model's first year, and the estimation expanded to broader age groups each year. The model calculated HBsAg prevalence among maternal age groups (21 to 30 years old) in 1984 and provided the HBV prevalence estimation among most high-risk groups (20 to 40 years old) in 2000.

The modeled HBV prevalence rate is compared with actual serosurveys to make adjustments. The Korean National Health and Nutrition Examination Survey (KNHANES) performed serosurveys to estimate the HBsAg prevalence among different age groups since 1998. We compared model results with serosurveys in 1998, 2010, and 2019.

After comparing and optimizing model inputs, the final parameters we used for maternal Maternal HBsAg prevalence, Maternal HBeAg prevalence, and Anti-HBc prevalence at ages 5 and 30 are listed in Table 2.7.1S. We adopted maternal HBsAg data from Ahn et al. (1992), which reviewed articles dealing with serological data for HBV infection published after 1980 and found that 6.5% of pregnant women in South Korea were HBsAg positive. In Ahn et al. (1992) study, HBeAg positivity was in 53.1% of HBsAg positive mothers. Goldstein et al. (2005) estimated 30% of HBeAg positivity among women of childbearing age, and leading models fit actual serosurveys better and are used in the study. The Anti-HBc prevalence rate at 5 years old is from Lee et al. (1997) study among 6-7 years old elementary school students born between 1980-1987; at 30 years old is from Park et al. (2010), the age group 20-29 years old

**Vaccination Coverage**

**Figure 2.7.1S. Comparison between vaccination coverage under the baseline and current vaccination coverage scenarios**

**
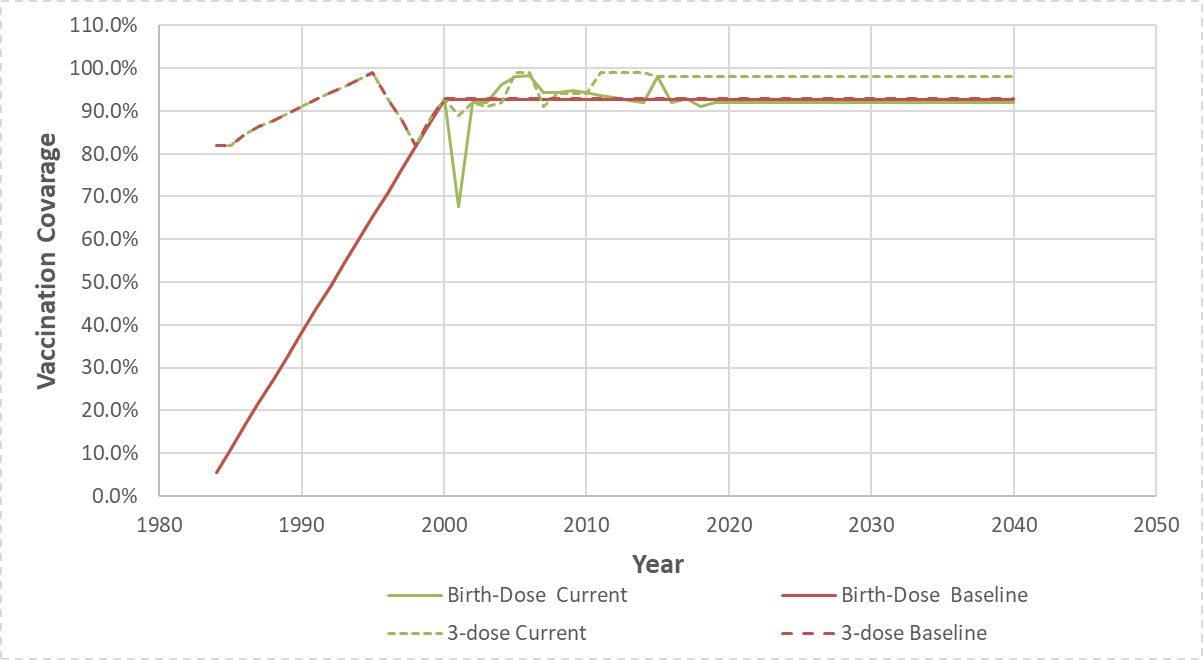
**

* WHO scenario not shown since South Korea has exceeded WHO vaccination goals

| **Table 2.7.1S. Summary of actual serosurvey data in South Korea for comparison** | | | | | | |
| --- | --- | --- | --- | --- | --- | --- |
| **Age Group** | **N** | **HBsAg** | **Lower 95% CI** | **Upper 95% CI** | **Survey year** | **Source** |
| 10-18 | 1654 | 2.20% | 1.42% | 2.98% | 1998 | KNHANES |
| 19-29 | 1648 | 5.10% | 3.73% | 6.47% |  |  |
| 30-39 | 1968 | 6.10% | 5.12% | 7.08% |  |  |
| 40-49 | 1630 | 5.10% | 4.12% | 6.08% |  |  |
| 50-59 | 1260 | 5.30% | 3.93% | 6.67% |  |  |
| 60-69 | 1007 | 2.90% | 1.72% | 4.08% |  |  |
| 70+ | 604 | 2.30% | 1.12% | 3.48% |  |  |
| 10-18 | 873 | 0.10% | -0.10% | 0.30% | 2010 | KNHANES |
| 19-29 | 739 | 2.10% | 0.92% | 3.28% |  |  |
| 30-39 | 1205 | 3.90% | 2.72% | 5.08% |  |  |
| 40-49 | 1137 | 3.70% | 2.52% | 4.88% |  |  |
| 50-59 | 1144 | 4.20% | 2.83% | 5.57% |  |  |
| 60-69 | 981 | 4.00% | 2.43% | 5.57% |  |  |
| 70+ | 736 | 1.80% | 0.82% | 2.78% |  |  |
| 10-18 | 626 | 0.00% | 0.00% | 0.00% | 2019 | KNHANES |
| 19-29 | 742 | 0.20% | 0.00% | 0.40% |  |  |
| 30-39 | 913 | 2.10% | 1.12% | 3.08% |  |  |
| 40-49 | 1104 | 3.40% | 2.22% | 4.58% |  |  |
| 50-59 | 1161 | 3.90% | 2.72% | 5.08% |  |  |
| 60-69 | 1112 | 3.50% | 2.32% | 4.68% |  |  |
| 70+ | 1107 | 1.50% | 0.72% | 2.28% |  |  |

| **Table 2.7.2S. Parameters for Model South Korea** | | |
| --- | --- | --- |
| **Parameter** | **Value** | **Source** |
| Maternal HBsAg prevalence | 6.50% | Ahn et al., 1992 |
| Maternal HBeAg prevalence | 30.00% | Goldstein et al., 2005 |
| Anti-HBc prevalence at age 5 | 9.00% | Lee et al., 1997 |
| Anti-HBc prevalence at age 30 | 66.00% | Park et al.,2010 |

**Model Validation**

1. Comparisons with the serosurvey:

**Figure 2.7.2S. Comparison between 1998, 2010 and 2019 modeled prevalence by age with the reported serosurvey data from KNHANES.**

1. **1998**

**
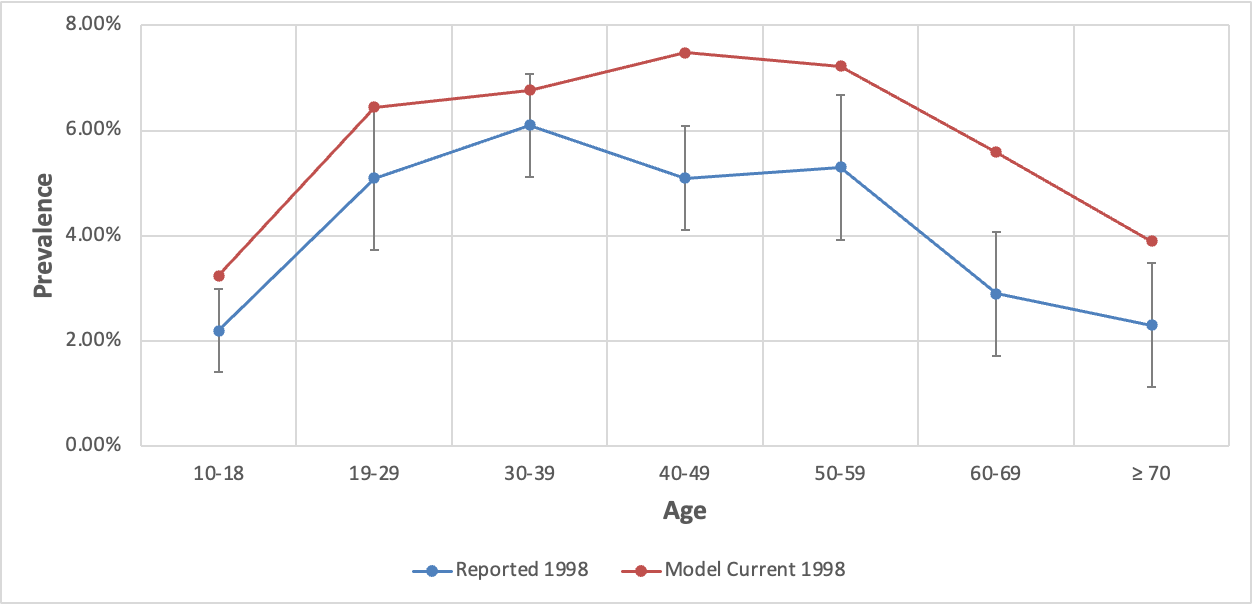
**

1. **2010**

**
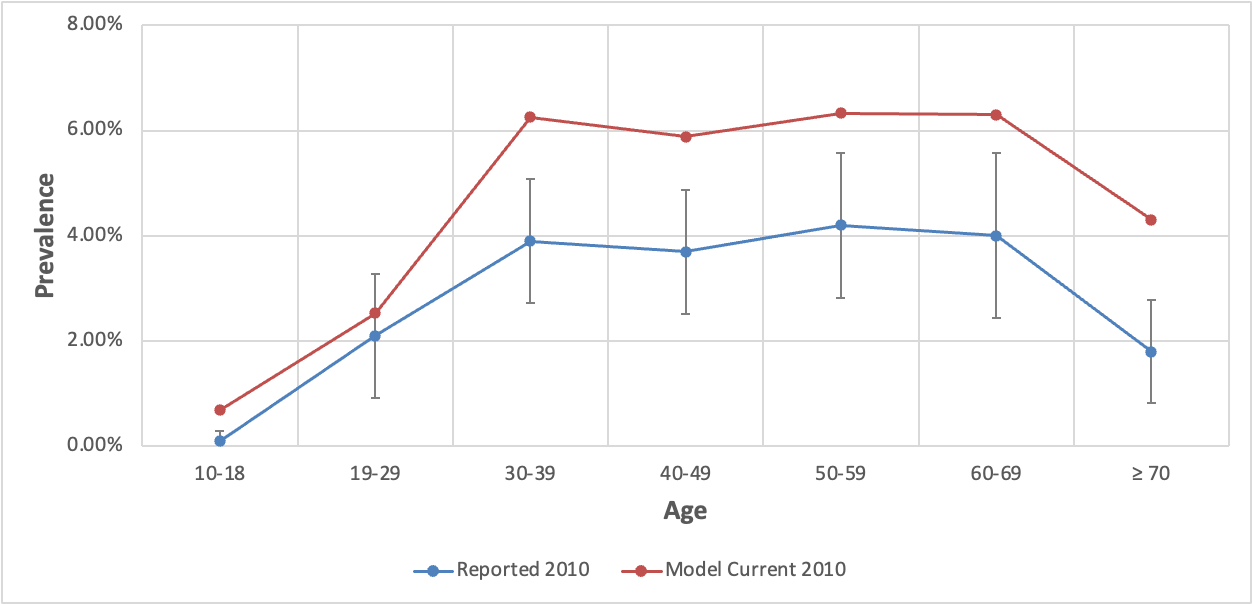
**

1. **2019**

**
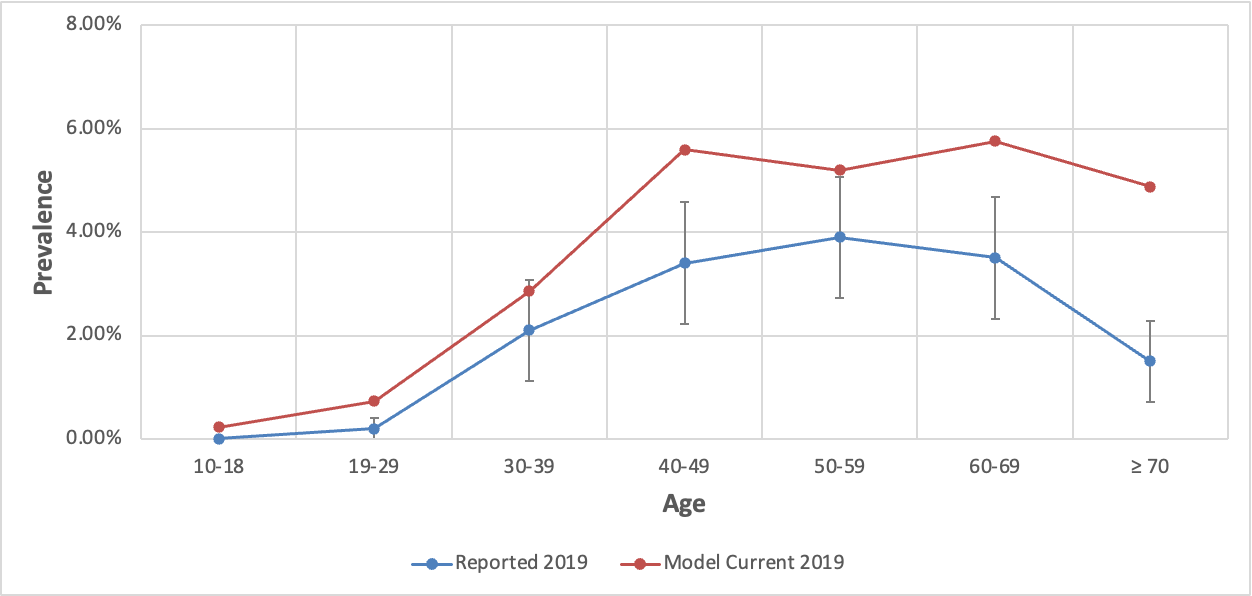
**

1. **Comparisons with Wong et al. (2019)**

**Figure 2.7.3S. Comparison between age-specific modeled prevalence with the overall prevalence in immigrants as estimated by Wong et al., 2019 (not stratified by age)**

**
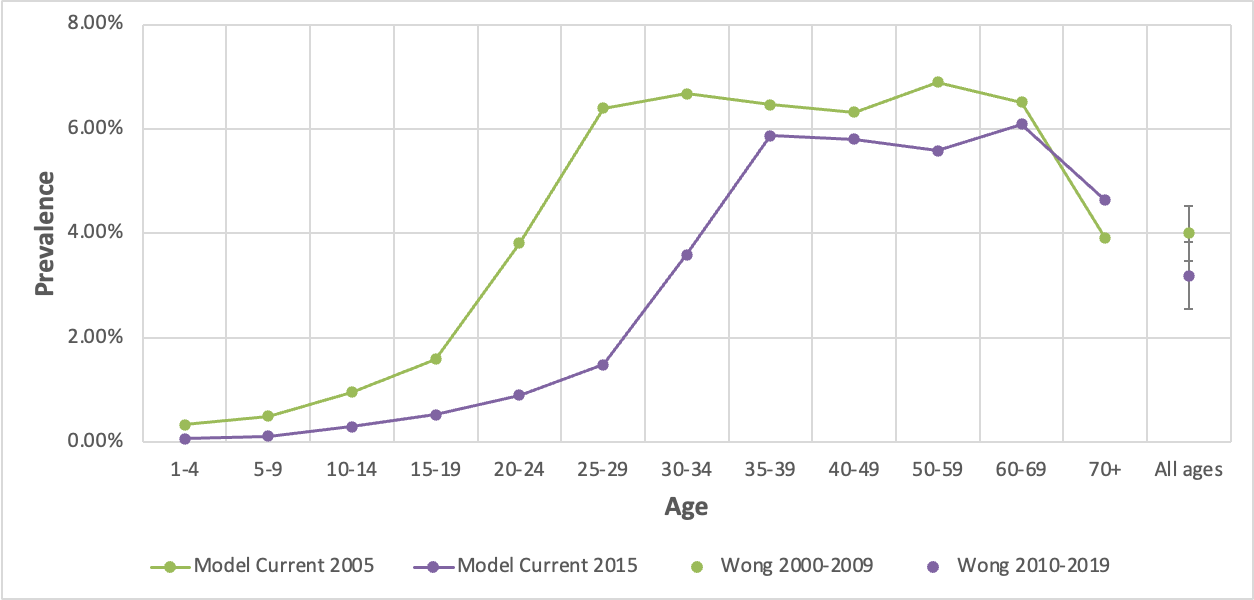
**

**Results**

**Figure 2.7.4.1S. Estimation of prevalence under the baseline and current vaccination coverage scenarios for a 5-year-old South Korean**


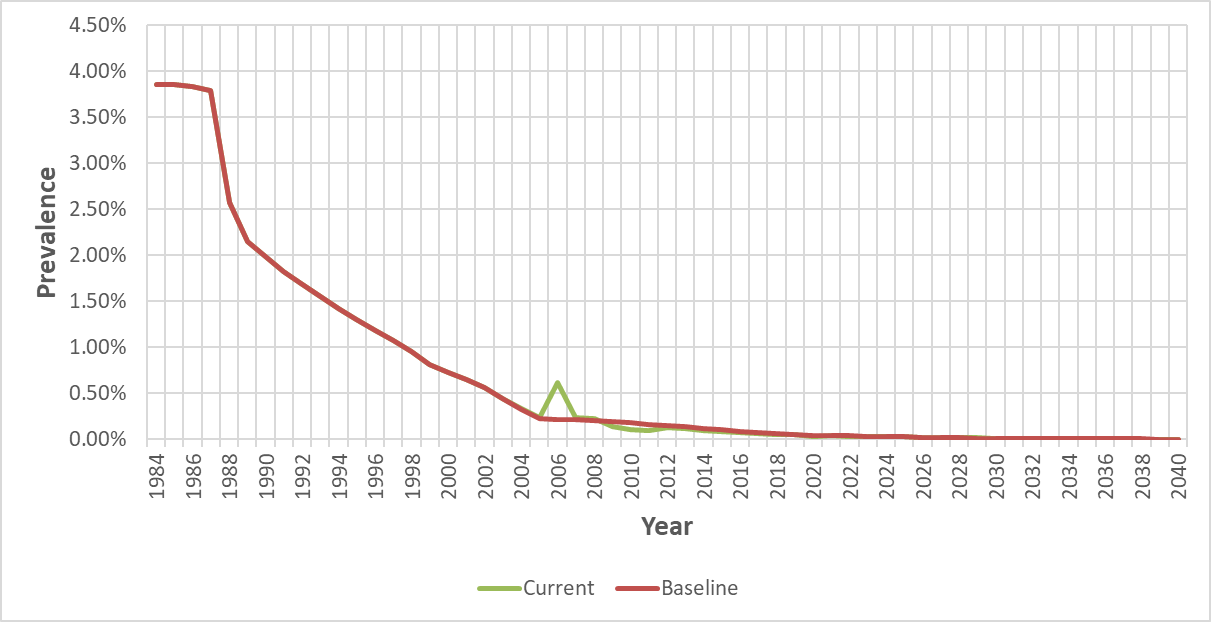


* WHO scenario not shown since South South Korea has exceeded WHO vaccination goals

**Figure 2.7.4.2S. Estimation of prevalence under the baseline and current vaccination coverage scenarios for a 20-year-old South Korean**


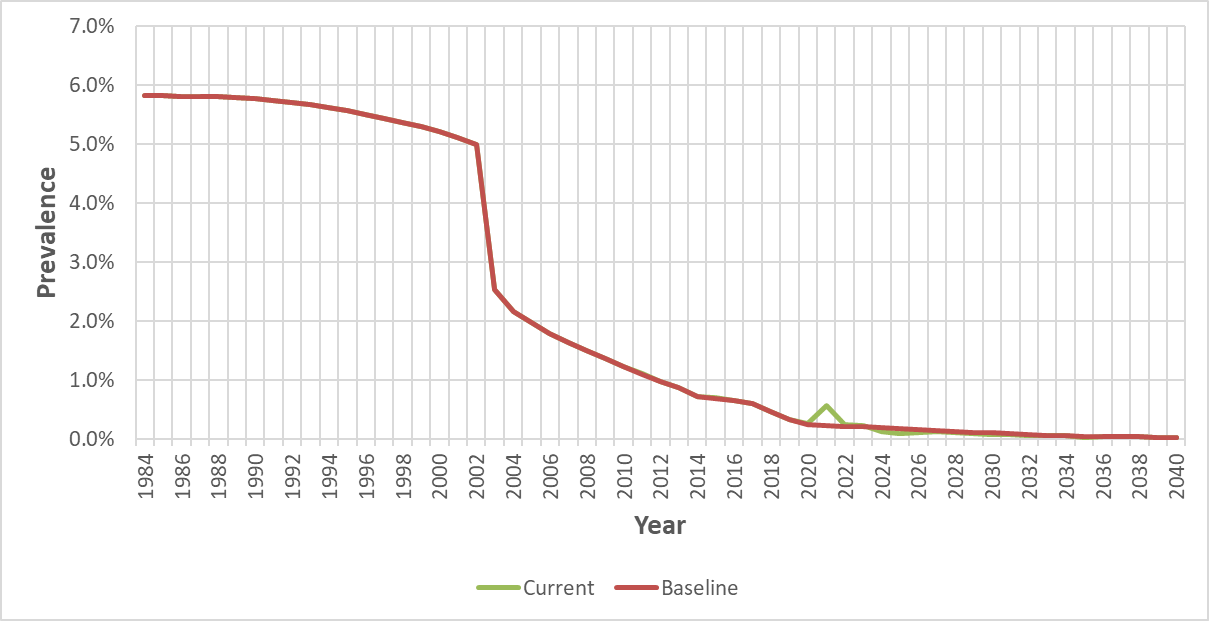


* WHO scenario not shown since South Korea has exceeded WHO vaccination goals

**Figure 2.7.5S. Estimation of immigrants with chronic hepatitis B under the baseline and current vaccination coverage scenarios in Korean**

**
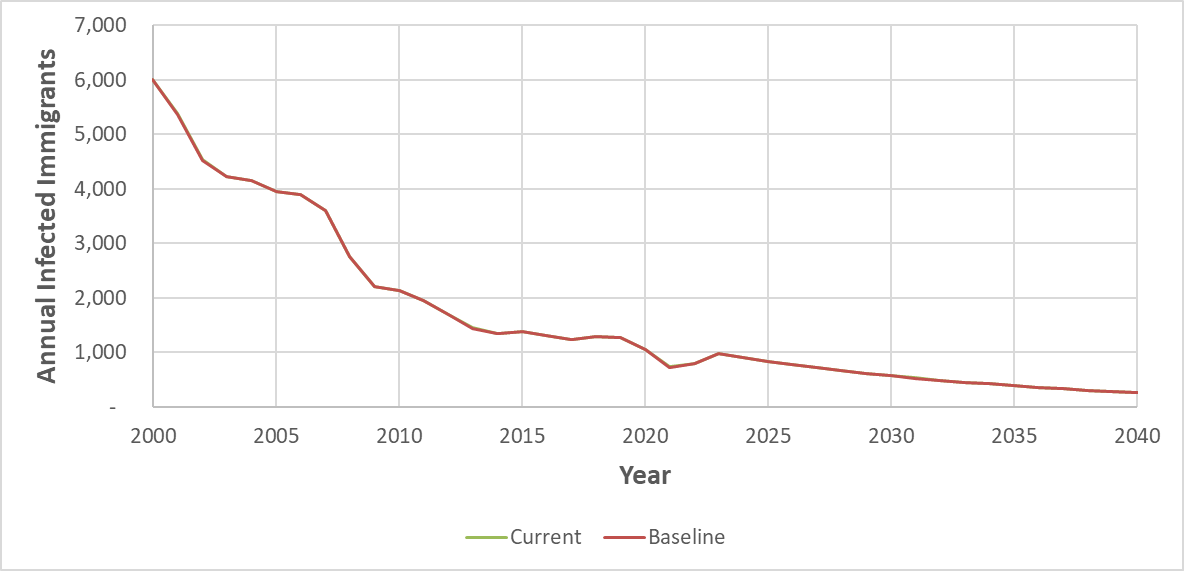
**

* WHO scenario not shown since South Korea has exceeded WHO vaccination goals

**References:**

Chen TW. Paths toward hepatitis B immunization in South Korea and Taiwan. Clin Exp Vaccine Res. 2013 Jul;2(2):76-82. doi: 10.7774/cevr.2013.2.2.76. Epub 2013 Jul 3. PMID: 23858397; PMCID: PMC3710927.

Lee SY, Choi B, Shin YJ, Bang KN, Ki M, Park HB, Yoon JD, Lee KC, Youn BJ. A Seroepidemiological Study on Hepatitis B Vaccination Program-In Elementary School Student from Kyonggi-Do Province. Korean Journal of Pediatric Infectious Diseases. 1997 Nov 1;4(2):240-56.

Korea Centers for Disease Control & Prevention (KCDC). Korea National Health and Nutrition Examination Survey (KNHANES), annual 1998 ~ 2020. Available from: <https://kosis.kr/index/index.do>

Ahn YO, Kim YS, Lee MS, Shin MH. Hepatitis B virus infection rate among Koreans.J Korean Med Sci. 1992 June;33(2):105-114.

Goldstein ST, Zhou F, Hadler SC, Bell BP, Mast EE, Margolis HS. A mathematical model to estimate global hepatitis B disease burden and vaccination impact. Int J Epidemiol. 2005 Dec;34(6):1329-39. doi: 10.1093/ije/dyi206. Epub 2005 Oct 25. PMID: 16249217.

Lee SY, Choi B, Shin YJ, Bang KN, Ki M, Park HB, Yoon JD, Lee KC, Youn BJ. A Seroepidemiological Study on Hepatitis B Vaccination Program-In Elementary School Student from Kyonggi-Do Province. Korean J. Pediatr. Infect. Dis. 1997 Nov 1;4(2):240-56. doi:10.14776/kjpid.1997.4.2.240

Park NH, Chung YH, Lee HS. Impacts of vaccination on hepatitis B viral infections in Korea over a 25-year period. Intervirology. 2010;53(1):20-8. doi: 10.1159/000252780. Epub 2010 Jan 5. PMID: 20068337.

**2.8 Mexico**

**Model Set-up**

Mexico implemented the universal hepatitis B vaccination by scheduling timely birth doses in 2007 and three-dose in 1999 (Ropero et al., 2017). We adopted vaccination coverage from WHO/UNICEF and literature reviews. WHO/UNICEF reported the data for birth dose and 3-dose hepatitis B vaccination in Mexico from 2020 to 2021 and 2000 to 2021. Prior to the data recorded by WHO/UNICEF, we adopted timely birth dose coverage data from WHO Administrative coverage data and Ropero Álvarez et al. (2017). We applied the linear estimation to fulfill this data gap for the birth dose and 3-dose vaccination rate unreported from 1984 to 2000 and from 1988 to 1994.

The HBV prevalence model is developed to estimate the number of CHB-infected Mexicans in the US after 2000. We initiated the Mexico model in 1960, 40 years before we calculated CHB-infected Mexican in 2000. Infants only at age 0 were estimated for the HBV prevalence in the model's first year, and the estimation expanded to broader age groups each year. The model calculated HBsAg prevalence among maternal age groups (21 to 30 years old) in 1999, and most high-risk HBV infected age groups (20 to 40 years old) in 2000.

The modeled HBV prevalence rate is compared with actual serosurveys to make adjustments. We compared our model with the prevalence of HBsAg by age group adopted from the 2018 National Health and Nutrition Survey (ENSANUT).

After comparing and optimizing model inputs, the final parameters we used for maternal Maternal HBsAg prevalence, Maternal HBeAg prevalence, and Anti-HBc prevalence at ages 5 and 30 are listed in Table 2.8.1S. Vázquez-Martínez et al. (2003) conducted a cross-sectional study in 2000, including 9,992 pregnant women attending the health services of the Mexican Institute. Their study found Maternal HBsAg prevalence at 1.65%, and Maternal HBeAg prevalence was 2.02%. Silveira et al. (1999) study investigated the seroprevalence of hepatitis B in over 12 000 subjects in six countries of Latin America and measured anti-HBc to determine hepatitis B infection. They found that the anti-HBc antibodies were 0.8% among groups 1-5 years old and 1.8% among groups 21-20 in Mexico.

**Vaccination Coverage**

**Figure 2.8.1S Comparison between vaccination coverage under the baseline and current vaccination coverage scenarios**


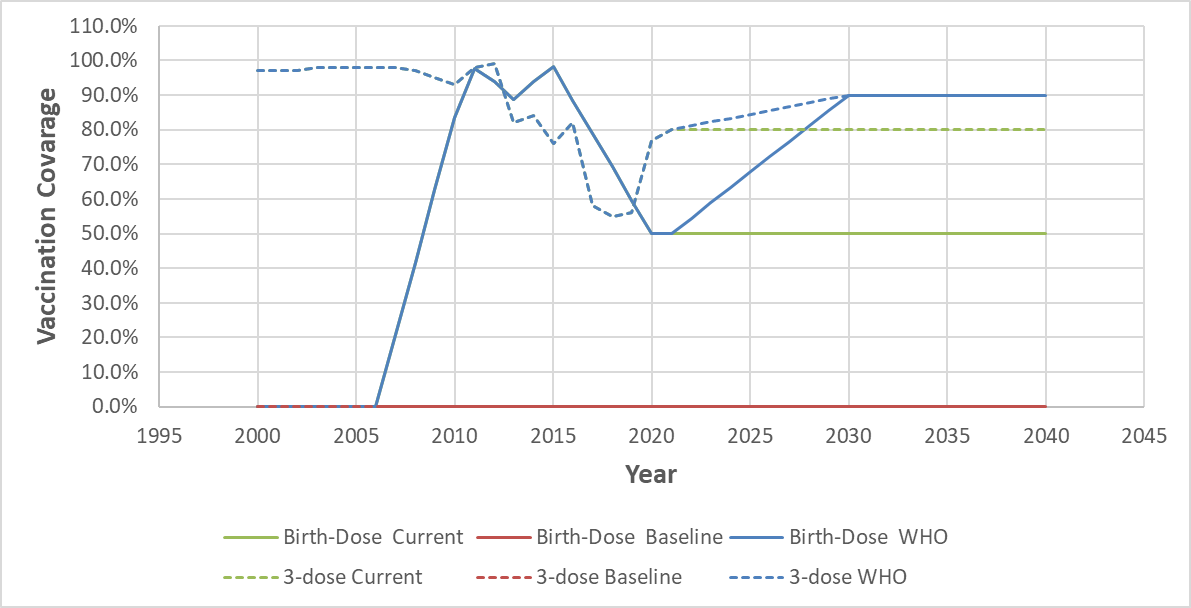


| **Table 2.8.1S Summary of actual serosurvey data in Mexico for comparison** | | | | | | |
| --- | --- | --- | --- | --- | --- | --- |
| **Age Group** | **N** | **HBsAg** | **Lower 95% CI** | **Upper 95% CI** | **Survey year** | **Source** |
| 20-39 | - | 0.00% | 0.00% | 0.00% | 2018 ENSANUT | Carnalla et al., 2021 |
| 40-49 | - | 0.12% | 0.02% | 1.09% |  |  |
| 50-59 | - | 0.00% | 0.00% | 0.00% |  |  |
| 60-69 | - | 2.09% | 0.66% | 5.74% |  |  |
| 70- | - | 0.36% | 0.02% | 0.88% |  |  |

| **Table 2.8.2S Parameters for Model Mexico** | | |
| --- | --- | --- |
| **Parameter** | **Value** | **Source** |
| Maternal HBsAg prevalence | 1.65% | Vázquez-Martínez et al., 2003 |
| Maternal HBeAg prevalence | 2.02% | Vázquez-Martínez et al., 2003 |
| Anti-HBc prevalence at age 5 | 0.80% | Silveira et al., 1999 |
| Anti-HBc prevalence at age 30 | 1.80% | Silveira et al., 1999 |

**Model Validation**

1. Comparisons with the serosurvey:

**Figure 2.8.2S Comparison between 2018 modeled prevalence by age with the reported serosurvey data from ENSANUT**


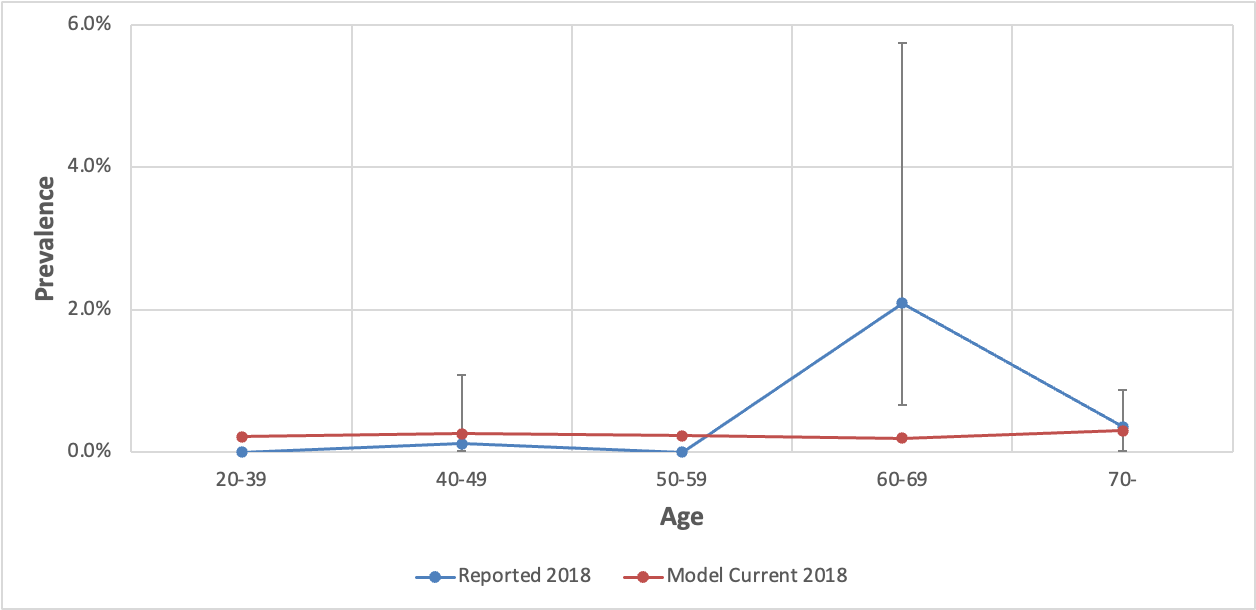


1. Comparisons with Wong et al. (2019)

**Figure 2.8.3S Comparison between age-specific modeled prevalence with the overall prevalence in immigrants as estimated by Wong et al., 2019 (not stratified by age)**


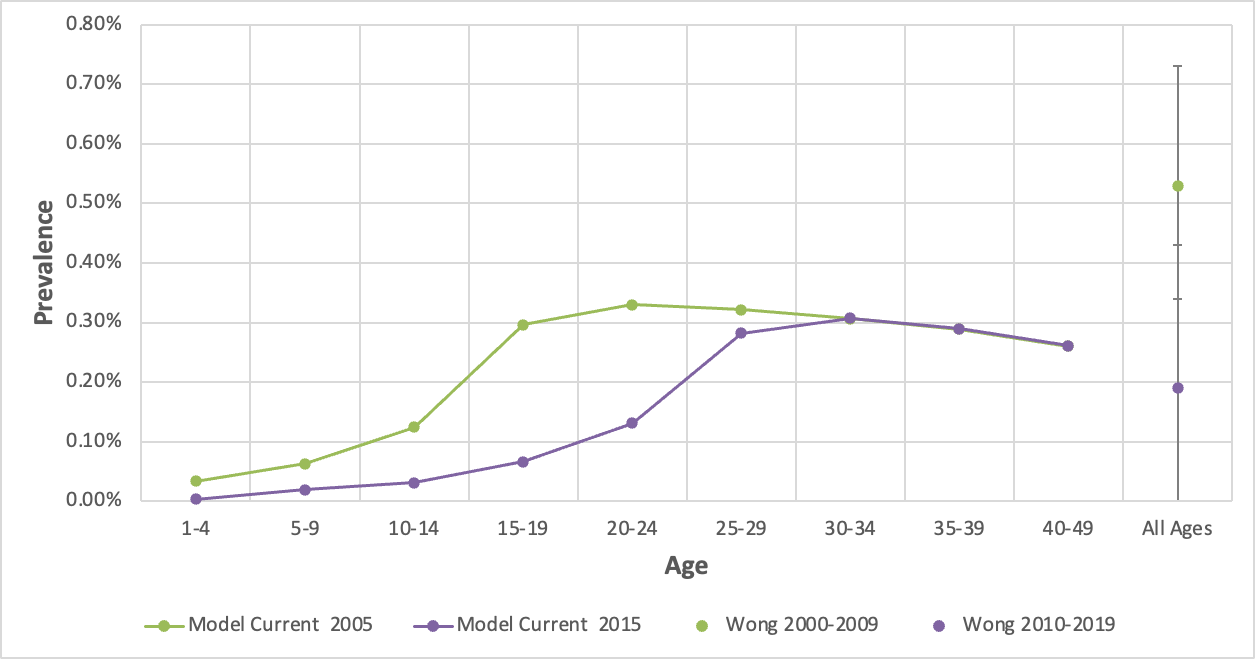


**Results**

**Figure 2.8.4.1S Estimation of prevalence under the baseline and current vaccination coverage scenarios for a 5-year-old Mexican**

**
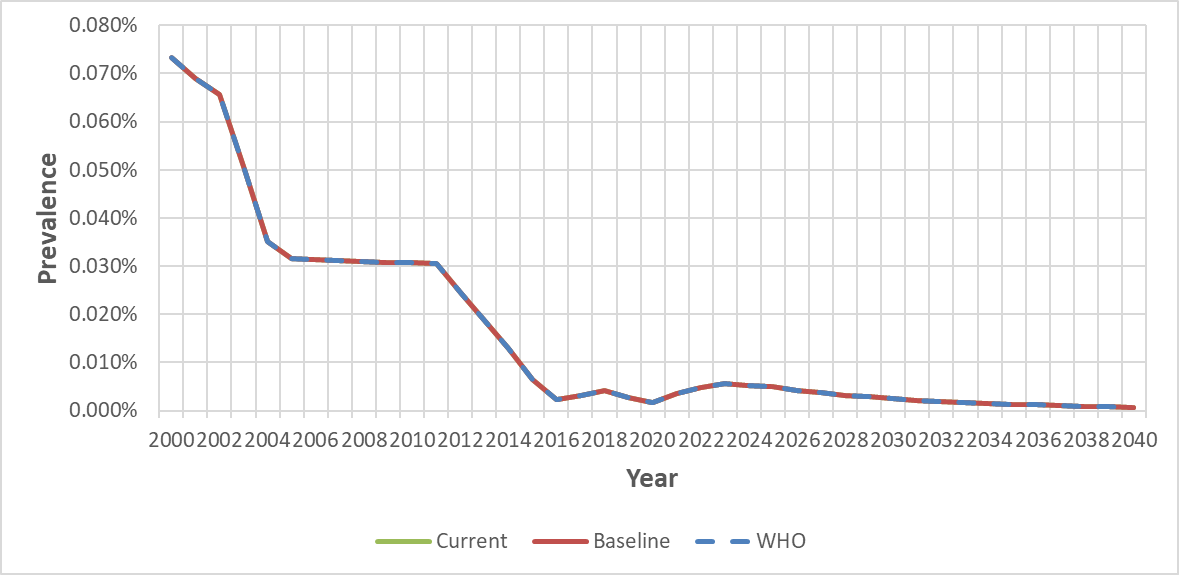
**

**Figure 2.8.4.2S Estimation of prevalence under the baseline and current vaccination coverage scenarios for a 20-year-old Mexican**


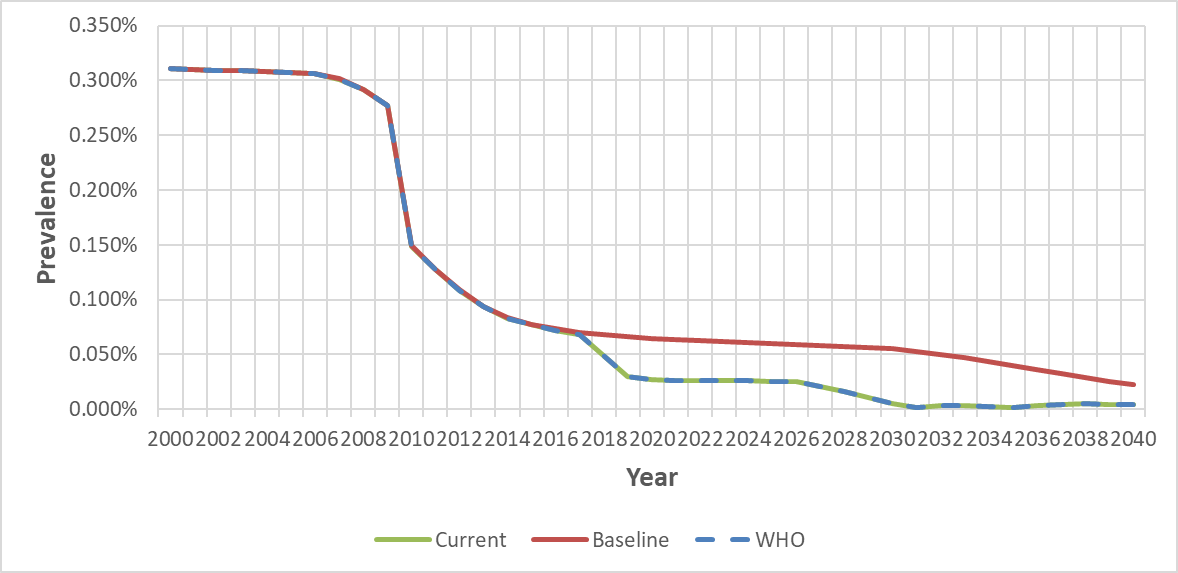


**Figure 2.8.5S Estimation of immigrants with chronic hepatitis B under the baseline and current vaccination coverage scenarios in Mexican**


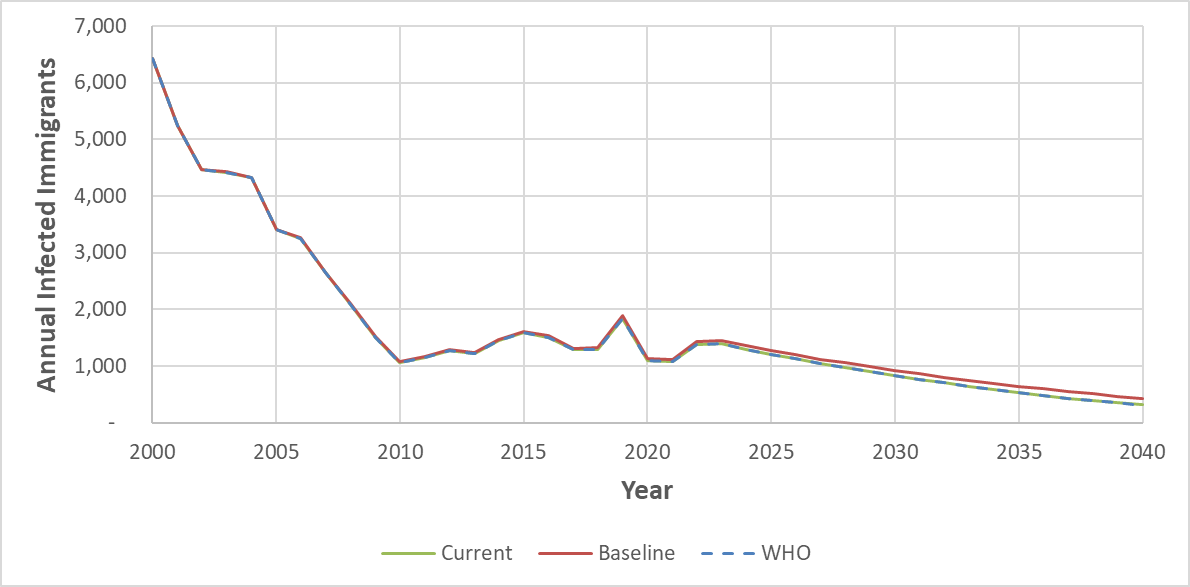


**References:**

Carnalla M, Vidaña-Pérez D, Alpuche-Aranda C, Chávez-Tapia NC, Romero-Martínez M, Shamah-Levy T, Barrientos-Gutiérrez T. Hepatitis B infection in Mexican adults: Results of a nationally representative survey. Ann Hepatol. 2022 Jan;27 Suppl 1:100583. doi: 10.1016/j.aohep.2021.100583. Epub 2021 Nov 19. PMID: 34808394.

Vázquez-Martínez JL, Coreño-Juárez MO, Montaño-Estrada LF, Attlan M, Gómez-Dantés H. Seroprevalence of hepatitis B in pregnant women in Mexico. Salud Publica Mex. 2003 May-Jun;45(3):165-70. doi: 10.1590/s0036-36342003000300005. PMID: 12870417.

Silveira TR, da Fonseca JC, Rivera L, Fay OH, Tapia R, Santos JI, Urdeneta E, Clemens SA. Hepatitis B seroprevalence in Latin America. Rev Panam Salud Publica. 1999 Dec;6(6):378-83. doi: 10.1590/s1020-49891999001100002. PMID: 10659668.

Ropero Álvarez AM, Pérez-Vilar S, Pacis-Tirso C, Contreras M, El Omeiri N, Ruiz-Matus C, Velandia-González M. Progress in vaccination towards hepatitis B control and elimination in the Region of the Americas. BMC Public Health. 2017 Apr 17;17(1):325. doi: 10.1186/s12889-017-4227-6. PMID: 28415981; PMCID: PMC5392937.

**2.9 Nigeria**

**Model Set-up**

Nigeria introduced the hepatitis B vaccine into the National Program on Immunization in 2004. We adopted vaccination coverage from WHO/UNICEF, which estimated the vaccination coverage from 2007 to 2021 for timely birth doses and from 2005 to 2021 for HepB 3-dose.

The HBV prevalence model is developed to estimate the number of CHB-infected Nigerians in the US after 2000. We initiated the Nigeria model in 1960, 40 years before we calculated CHB-infected Nigeria in 2000. Infants only at age 0 were estimated for the HBV prevalence in the model's first year, and the estimation expanded to broader age groups each year. The model calculated HBsAg prevalence among maternal age groups (21 to 30 years old) in 1999, and most high-risk HBV infected age groups (20 to 40 years old) in 2000.

The modeled HBV prevalence rate is compared with actual serosurveys to make adjustments. Hepatitis B prevalence varies in different parts of Nigeria (Fakunle et al., 1981). This study adopted the prevalence of HBsAg by age group from three studies to compare and correct the current model. Adoga et al. (2010) collected blood samples from 1,891 subjects during pre-vaccination tests between 2008 and 2009; Oje et al. (2012) obtained the HBV transmission data from 2,000 individuals at apparently-health patients visiting health centers in 2009; and Onyekwere et al. (2014) conducted a nationwide population survey from 2010 to 2012, screening HBsAg for 5,558 adults.

After comparing and optimizing model inputs, the final parameters we used for maternal Maternal HBsAg prevalence, Maternal HBeAg prevalence, and Anti-HBc prevalence at ages 5 and 30 are listed in Table 2.9.1S. The maternal HBsAg prevalence data comes from Musa et al (2015), which estimated HBV prevalence through selecting systematic reviews and meta-analyses from 2000 to 2013, and reviewed 14 studies for Hepatitis B among pregnant women. HBeAg was present in 16.4% HBsAg positive individuals in Amazigo et al. (1990) study, which measured hepatitis B virus markers in the sera of 804 rural and urban inhabitants and prisoners in Nigeria. Nasidi et al. (1986) estimated the prevalence of hepatitis B infection markers in different groups, finding the Anti-HBc at age 5 years and 30 years old among healthy children among 5-6 years old and healthy females aged 12 and 45 years.

**Vaccination Coverage**

**Figure 2.9.1S. Comparison between vaccination coverage under the baseline and current vaccination coverage scenarios**

**
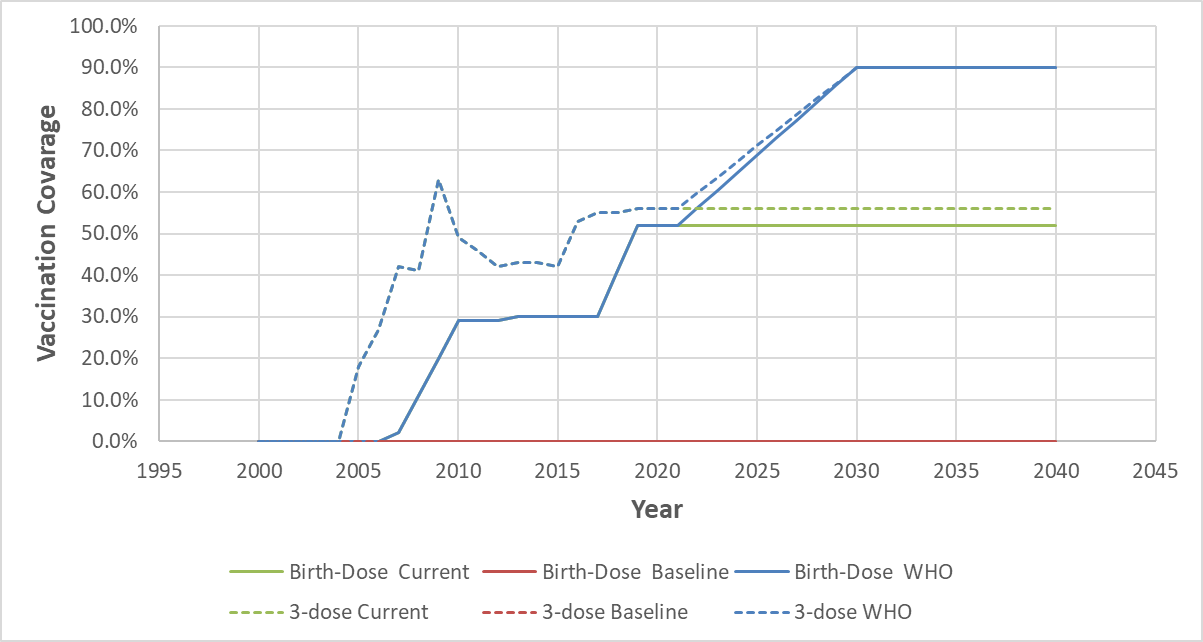
**

| **Table 2.9.1S. Summary of actual serosurvey data in Nigeria for comparison** | | | | | | |
| --- | --- | --- | --- | --- | --- | --- |
| **Age Group** | **N** | **HBsAg** | **Lower 95% CI** | **Upper 95% CI** | **Survey year** | **Source** |
| -10 | 409 | 2.90% | 1.27% | 4.53% | 2008-2009 | Adoga et al., 2010 |
| 11-20 | 647 | 6.50% | 4.60% | 8.40% |  |  |
| 21-30 | 401 | 9.20% | 6.37% | 12.03% |  |  |
| 31-40 | 264 | 6.40% | 3.45% | 9.35% |  |  |
| 41-50 | 138 | 4.30% | 0.92% | 7.68% |  |  |
| 51-60 | 32 | 0.00% | 0.00% | 0.00% |  |  |
| -24 | 944 | 4.66% | 3.32% | 6.01% | 2009 | Oje et al., 2010 |
| 25-34 | 372 | 4.30% | 2.24% | 6.36% |  |  |
| 35-44 | 332 | 21.69% | 17.25% | 26.12% |  |  |
| 45-54 | 216 | 20.37% | 15.00% | 25.74% |  |  |
| 55- | 136 | 14.71% | 8.75% | 20.66% |  |  |
| -20 | 26 | 3.80% | 0.00% | 11.15% | 2010-2012 | Onyekwere et al., 2014 |
| 20-29 | 1219 | 6.00% | 4.67% | 7.33% |  |  |
| 30-39 | 2355 | 7.40% | 6.34% | 8.46% |  |  |
| 40-49 | 1240 | 7.30% | 5.85% | 8.75% |  |  |
| 50-59 | 386 | 4.90% | 2.75% | 7.05% |  |  |
| 60- | 49 | 4.10% | 0.00% | 9.65% |  |  |

| **Table 2.9.2S. Parameters for Model Nigeria** | | |
| --- | --- | --- |
| **Parameter** | **Value** | **Source** |
| Maternal HBsAg prevalence | 14.10% | Musa et al., 2015 |
| Maternal HBeAg prevalence | 16.40% | Amazigo et al., 1990 |
| Anti-HBc prevalence at age 5 | 18.90% | Nasidi et al., 1986 |
| Anti-HBc prevalence at age 30 | 68.10% | Nasidi et al., 1986 |

**Model Validation**

1. Comparisons with the serosurvey:

**Figure 2.9.2S. Comparison between 2008, 2009, and 2010 modeled prevalence by age with the reported serosurvey data from Adoga et al., 2010, Oje et al., 2012, and Onyekwere et al., 2014.**

1. **2008**

**
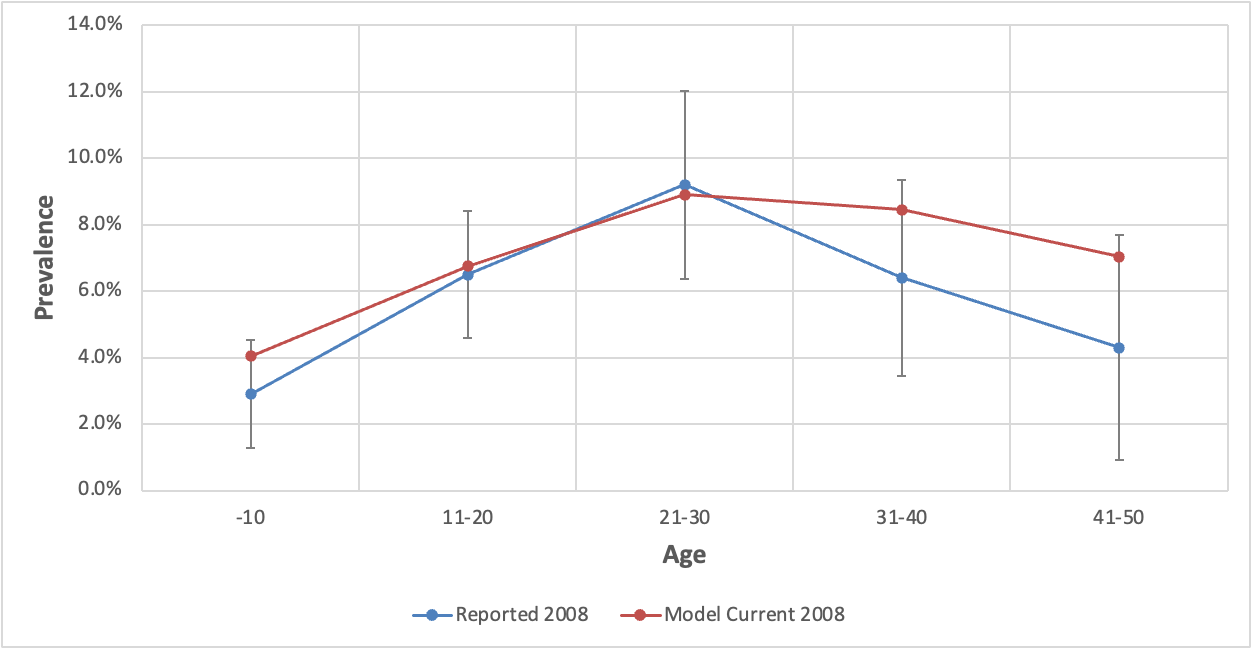
**

1. **2009**


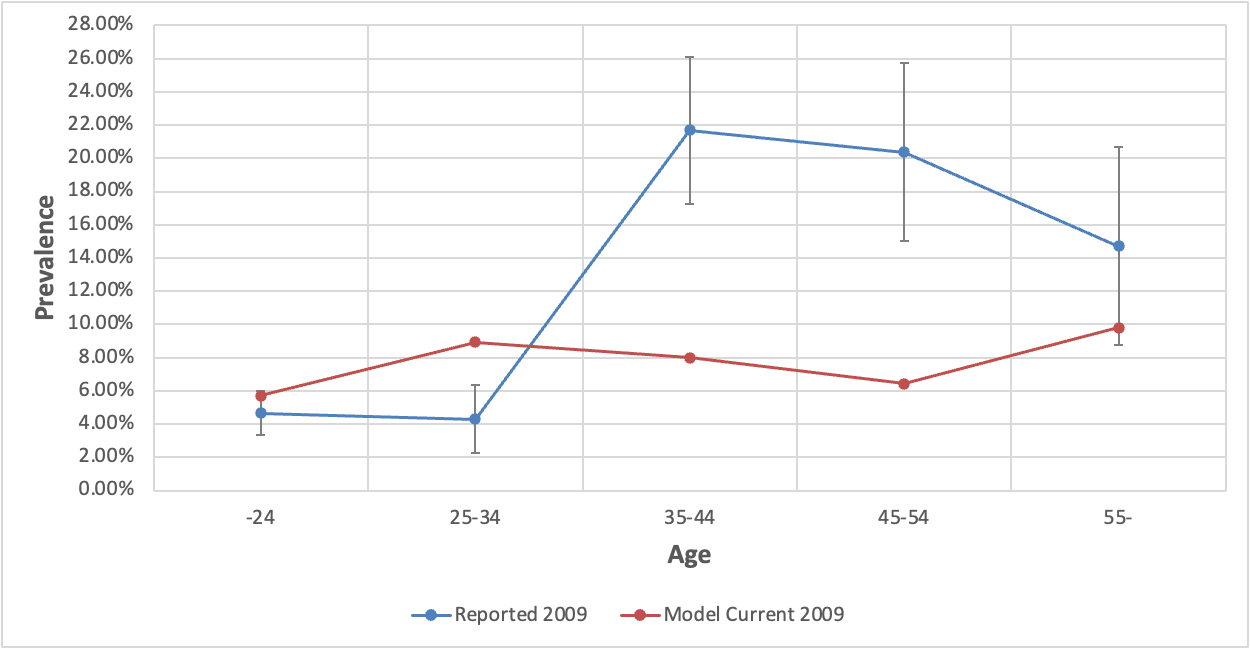


1. **2010**

**
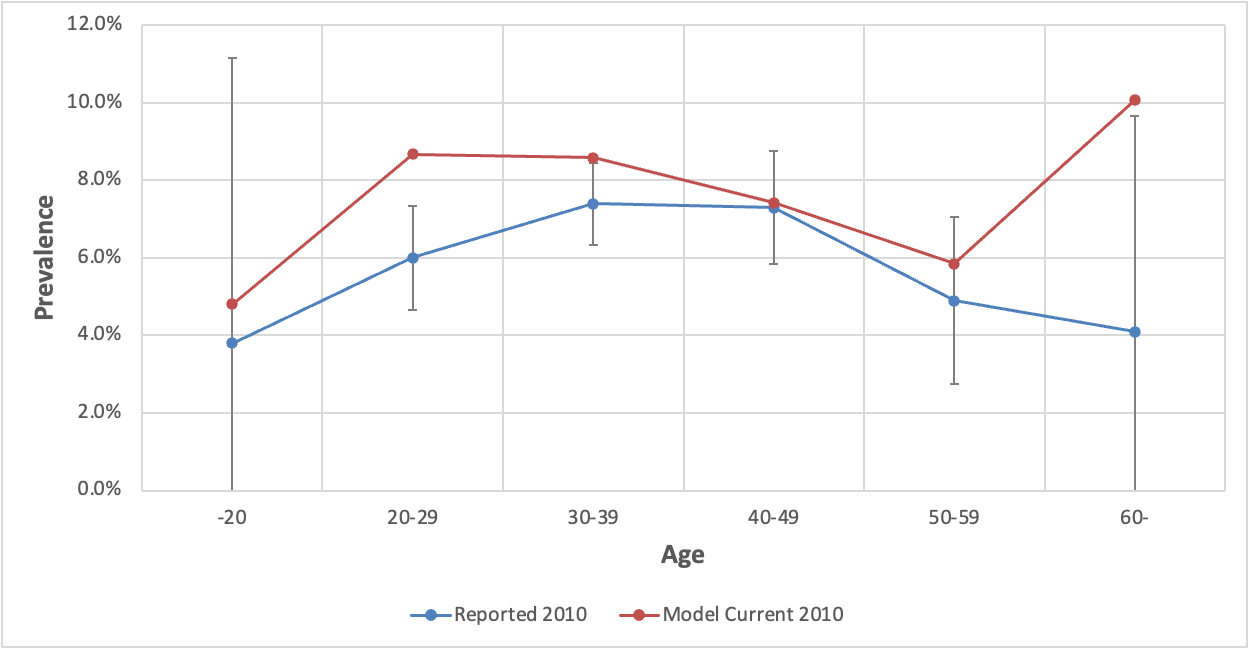
**

1. Comparisons with Wong et al. (2019)

**Figure 2.9.3S Comparison between age-specific modeled prevalence with the overall prevalence in immigrants as estimated by Wong et al., 2019 (not stratified by age)**


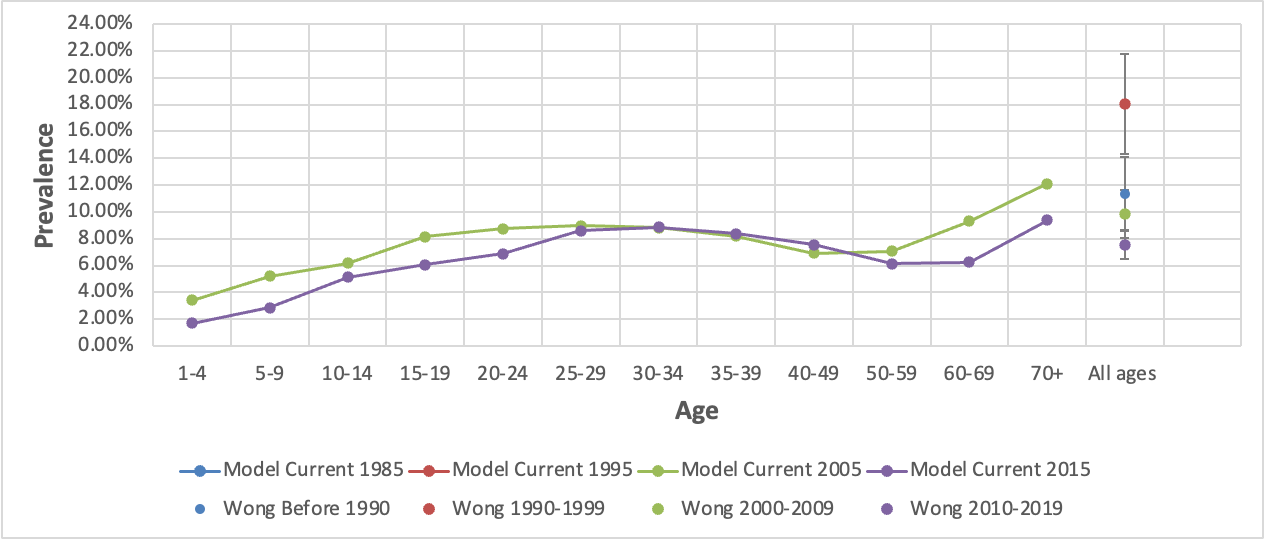


**Results**

**Figure 2.9.4.1S. Estimation of prevalence under the baseline and current vaccination coverage scenarios for a 5-year-old Nigeria**


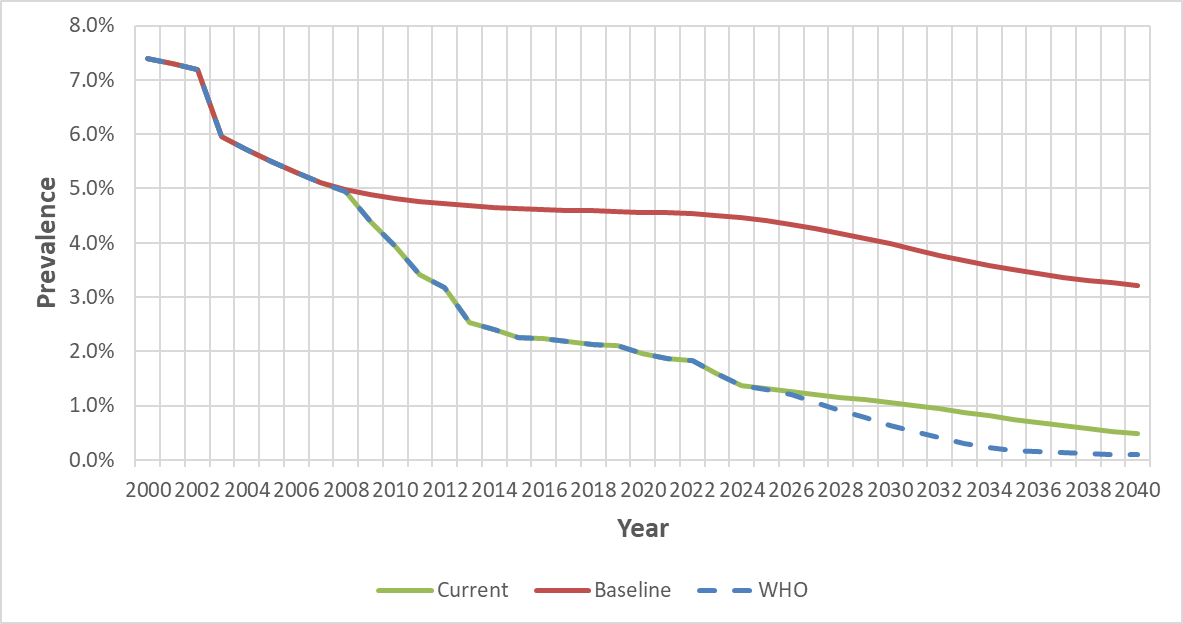


**Figure 2.9.4.2S. Estimation of prevalence under the baseline and current vaccination coverage scenarios for a 20-year-old Nigeria**

**
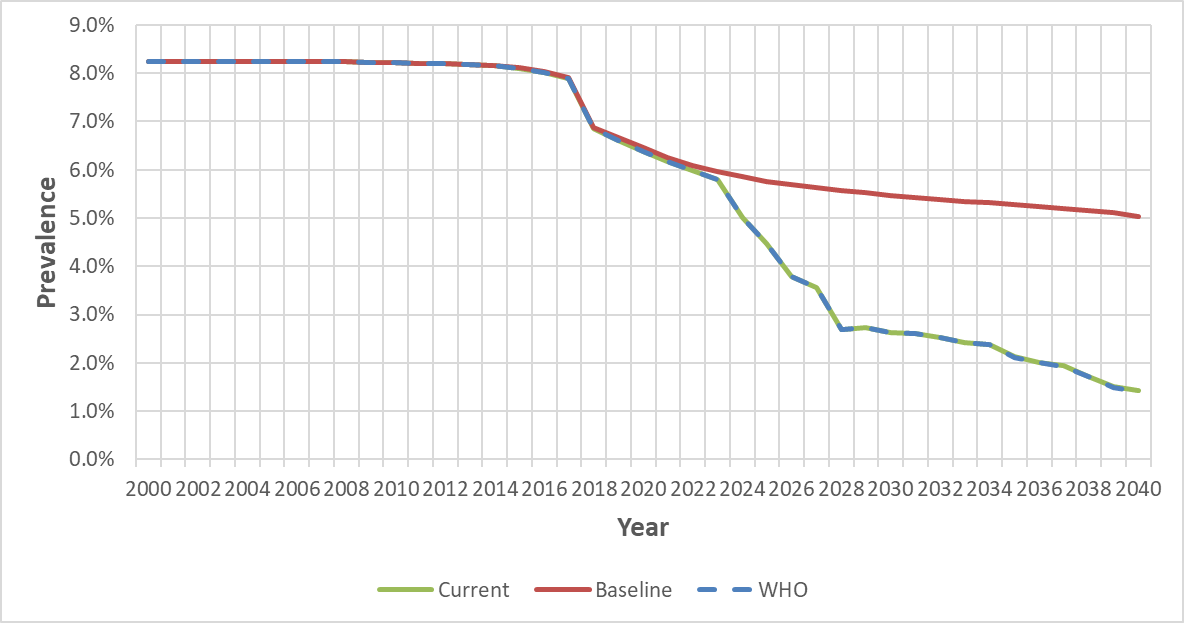
**

**Figure 2.9.5S. Estimation of immigrants with chronic hepatitis B under the baseline and current vaccination coverage scenarios in Nigerian**


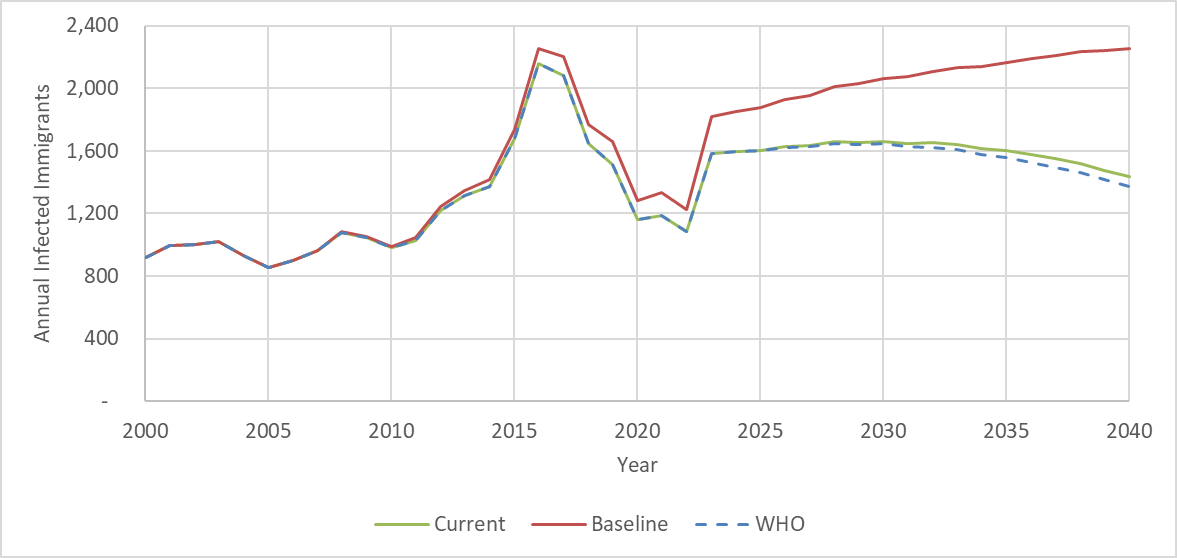


**References:**

Fakunle YM, Abdurrahman MB, Whittle HC. Hepatitis-B virus infection in children and adults in Northern Nigeria: a preliminary survey. Trans R Soc Trop Med Hyg. 1981;75(5):626-9. doi: 10.1016/0035-9203(81)90133-4. PMID: 7330917.

Adoga MP, Gyar SD, Pechulano S, Bashayi OD, Emiasegen SE, Zungwe T, Iperepolu OH, Agupugo C, Agwale SM. Hepatitis B virus infections in apparently healthy urban Nigerians: data from pre-vaccination tests. J Infect Dev Ctries. 2010 Jun 30;4(6):397-400. PMID: 20601793.

Oje OJ, Sule WF, Famurewa D. Dual positivity of hepatitis B surface antigen and anti-hepatitis C virus antibody and associated factors among apparently healthy patients of Ekiti State, Nigeria. Viral Immunol. 2012 Dec;25(6):448-55. doi: 10.1089/vim.2012.0042. Epub 2012 Nov 21. PMID: 23171358.

Onyekwere CA, Hameed L. Hepatitis B and C virus prevalence and association with demographics: report of population screening in Nigeria. Trop Doct. 2015 Oct;45(4):231-5. doi: 10.1177/0049475514560211. Epub 2014 Dec 15. PMID: 25515733.

Musa BM, Bussell S, Borodo MM, Samaila AA, Femi OL. Prevalence of hepatitis B virus infection in Nigeria, 2000-2013: a systematic review and meta-analysis. Niger J Clin Pract. 2015 Mar-Apr;18(2):163-72. doi: 10.4103/1119-3077.151035. PMID: 25665986.

Amazigo UO, Chime AB. Hepatitis-B virus infection in rural and urban populations of eastern Nigeria: prevalence of serological markers. East Afr Med J. 1990 Aug;67(8):539-44. PMID: 2261867.

Nasidi A, Harry TO, Vyazov SO, Munube GM, Azzan BB, Ananiev VA. Prevalence of hepatitis B infection markers in representative areas of Nigeria. Int J Epidemiol. 1986 Jun;15(2):274-6. doi: 10.1093/ije/15.2.274. PMID: 3721692.

**2.10 Haiti**

**Model Set-up**

Haiti scheduled a three-dose hepatitis B vaccine in 2013, but still no birth dose available (Ropero et al., 2017). We adopted vaccination coverage from WHO/UNICEF, which estimated the 3-dose Hep B vaccination coverage from 2013 to 2021.

The HBV prevalence model is developed to estimate the number of CHB-infected Haitians in the US after 2000. We initiated the Haiti model in 1960, 40 years before we calculated CHB-infected Haiti in 2000. Infants only at age 0 were estimated for the HBV prevalence in the model's first year, and the estimation expanded to broader age groups each year. The model calculated HBsAg prevalence among maternal age groups (21 to 30 years old) in 1999, and most high-risk HBV infected age groups (20 to 40 years old) in 2000.

The modeled HBV prevalence rate is compared with actual serosurveys to make adjustments. There are not many studies classified HBsAg prevalence by age for Haitain. Tohme et al. (2016) study tested 1,307 pregnant women for HBsAg positivity rate among women greater than 15 years old, was adopted for the comparison.

After comparing and optimizing model inputs, the final parameters we used for maternal Maternal HBsAg prevalence, Maternal HBeAg prevalence, and Anti-HBc prevalence at ages 5 and 30 are listed in Table 2.10.1S. Centers for Disease Control and Prevention Global AIDS Program estimate of HBsAg prevalence taken in antenatal clinics among 15- to 49- year-old child-bearing Haitian women for whom the prevalence was 4.7% in 2004 (Rein et al., 2010). Maternal HBeAg prevalence rate was obtained from Goldstein et al (2015), which has the average prevalence value for similar prevalence countries. Anti-HBc prevalence at 5 years old and 30 years old from Goldstein et al (2015) and Tohme et al. (2016).

**Vaccination Coverage**

**Figure 2.10.1S. Comparison between vaccination coverage under the baseline and current vaccination coverage scenarios**


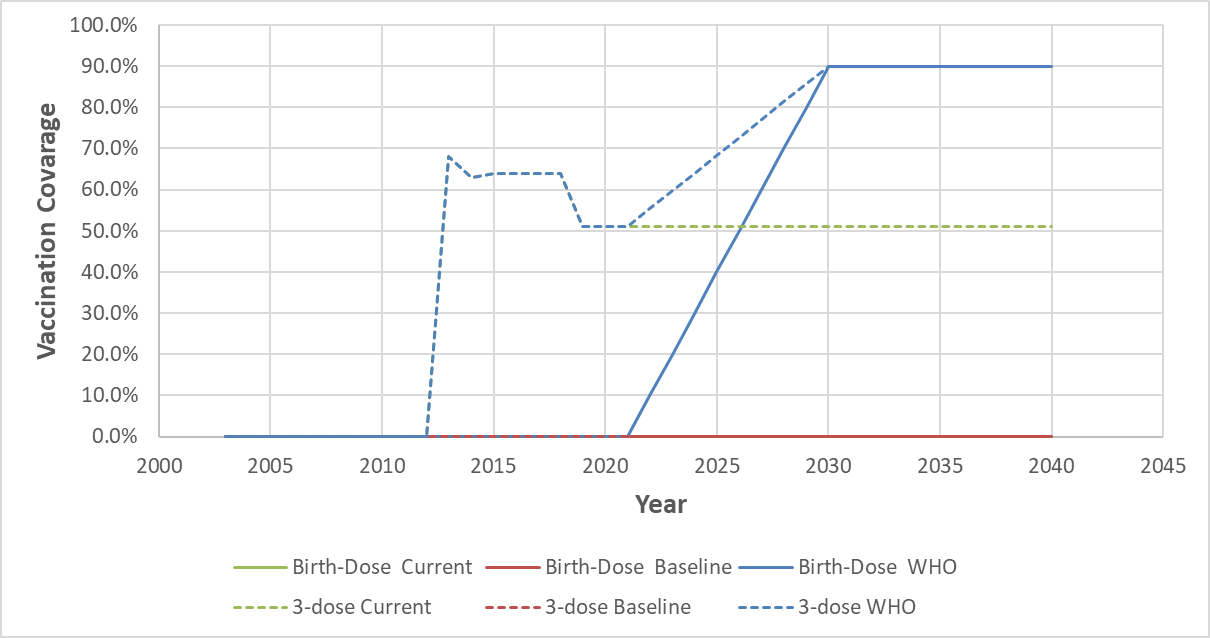


| **Table 2.10.1S. Summary of actual serosurvey data in Haiti for comparison** | | | | | | |
| --- | --- | --- | --- | --- | --- | --- |
| **Age Group** | **N** | **HBsAg** | **Lower 95% CI** | **Upper 95% CI** | **Survey year** | **Source** |
| 15-19 | 183 | 2.73% | 1.68% | 3.79% | 2012 | Tohme et al., 2016 |
| 20-24 | 352 | 1.14% | 0.58% | 1.69% |  |  |
| 25-29 | 356 | 1.97% | 1.42% | 2.51% |  |  |
| 30-34 | 241 | 3.73% | 2.94% | 4.53% |  |  |
| 35-39 | 130 | 3.85% | 2.37% | 5.32% |  |  |
| 40- | 45 | 6.67% | 2.46% | 10.87% |  |  |

| **Table 2.10.2S. Parameters for Model Haiti** | | |
| --- | --- | --- |
| **Parameter** | **Value** | **Source** |
| Maternal HBsAg prevalence | 4.70% | Rein et al., 2010 |
| Maternal HBeAg prevalence | 15.00% | Goldstein et al., 2005 |
| Anti-HBc prevalence at age 5 | 3.00% | Goldstein et al., 2005 |
| Anti-HBc prevalence at age 30 | 30.60% | Tohme et al., 2016 |

**Model Validation**

1. Comparisons with the serosurvey:

**Figure 2.10.2S. Comparison between 2012 modeled prevalence by age with the reported serosurvey data from Tohme et al., 2016.**


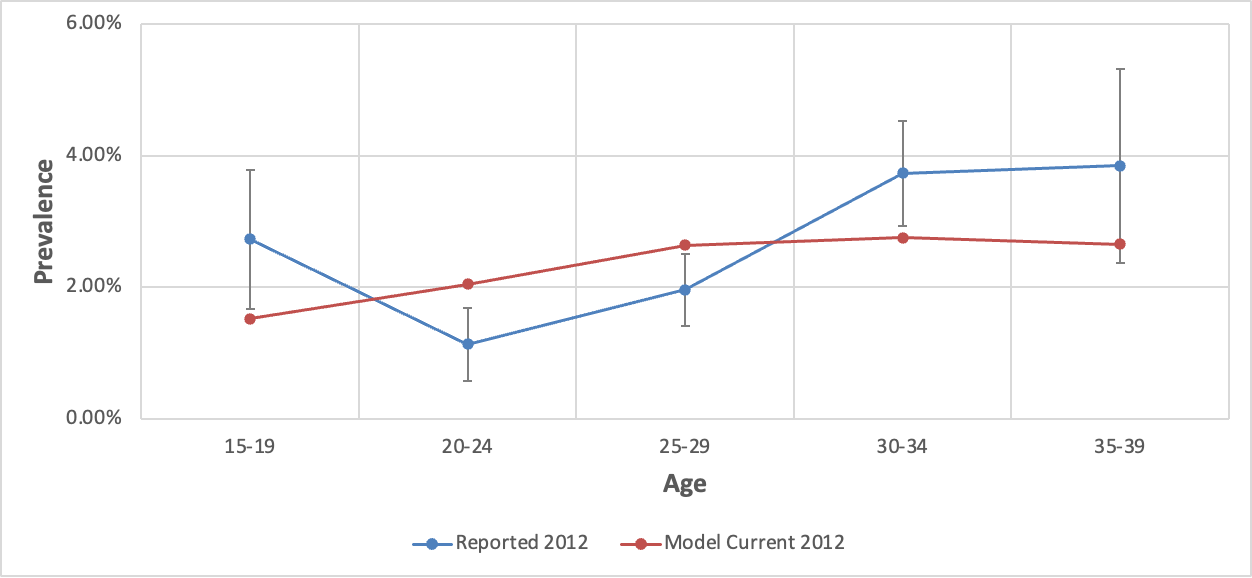


1. Comparisons with Wong et al. (2019)

**Figure 2.10.3S Comparison between age-specific modeled prevalence with the overall prevalence in immigrants as estimated by Wong et al., 2019 (not stratified by age)**


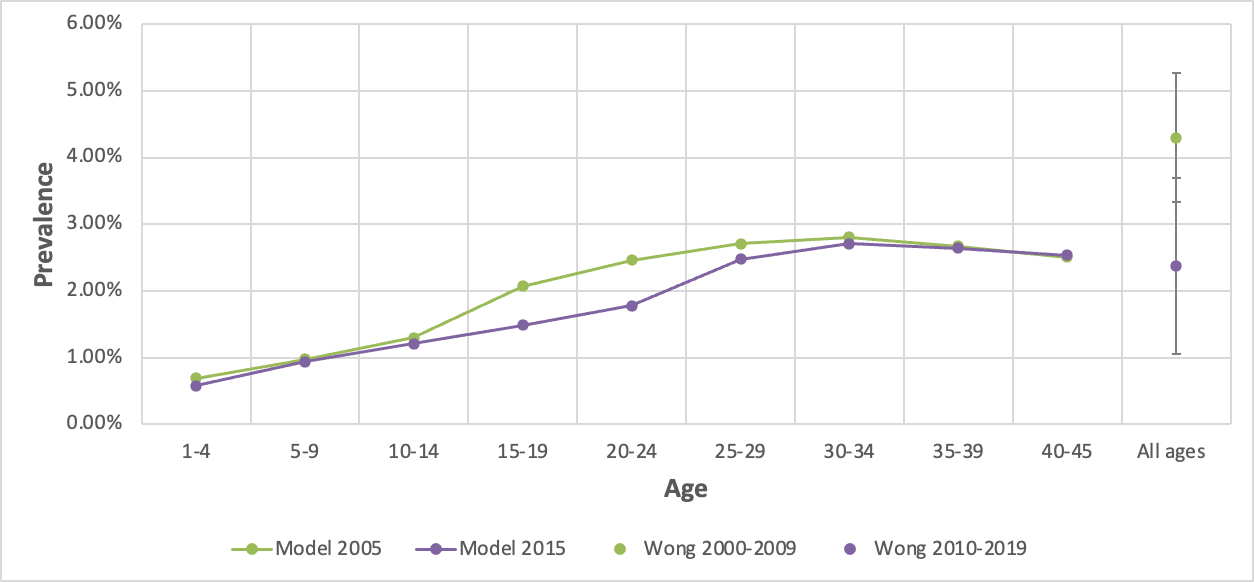


**Results**

**Figure 2.10.4.1S Estimation of prevalence under the baseline and current vaccination coverage scenarios for a 5-year-old Haitian**


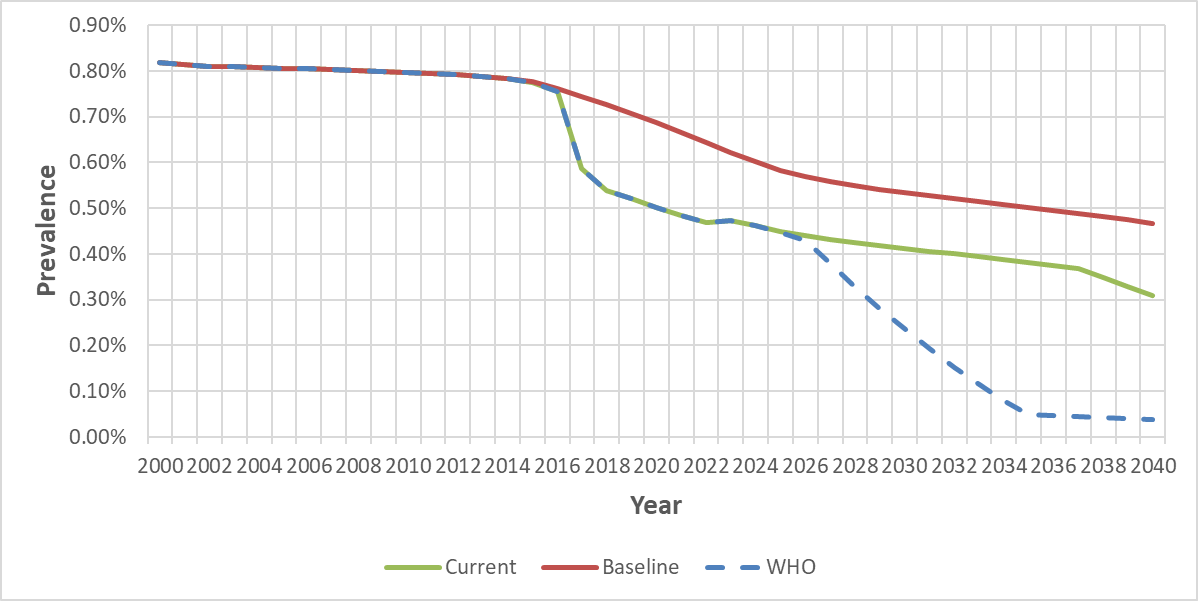


**Figure 2.10.4.2S Estimation of prevalence under the baseline and current vaccination coverage scenarios for a 20-year-old Haitian**

**
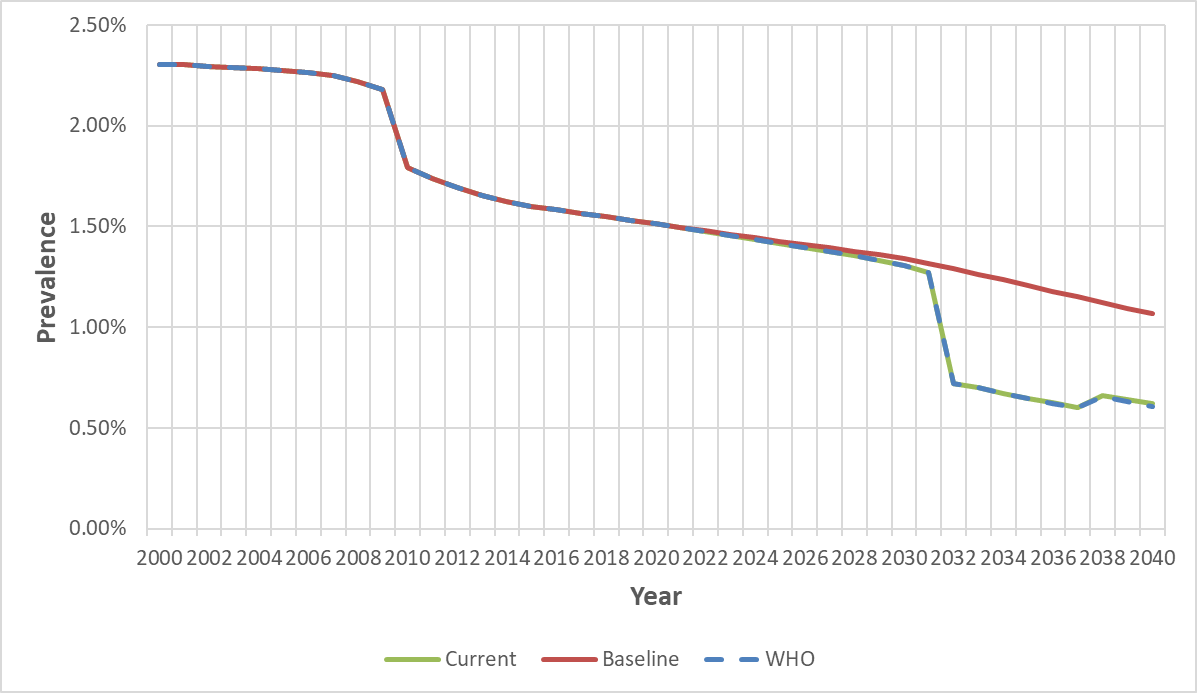
**

**Figure 2.10.5S Estimation of immigrants with chronic hepatitis B under the baseline and current vaccination coverage scenarios in Haitian**

**
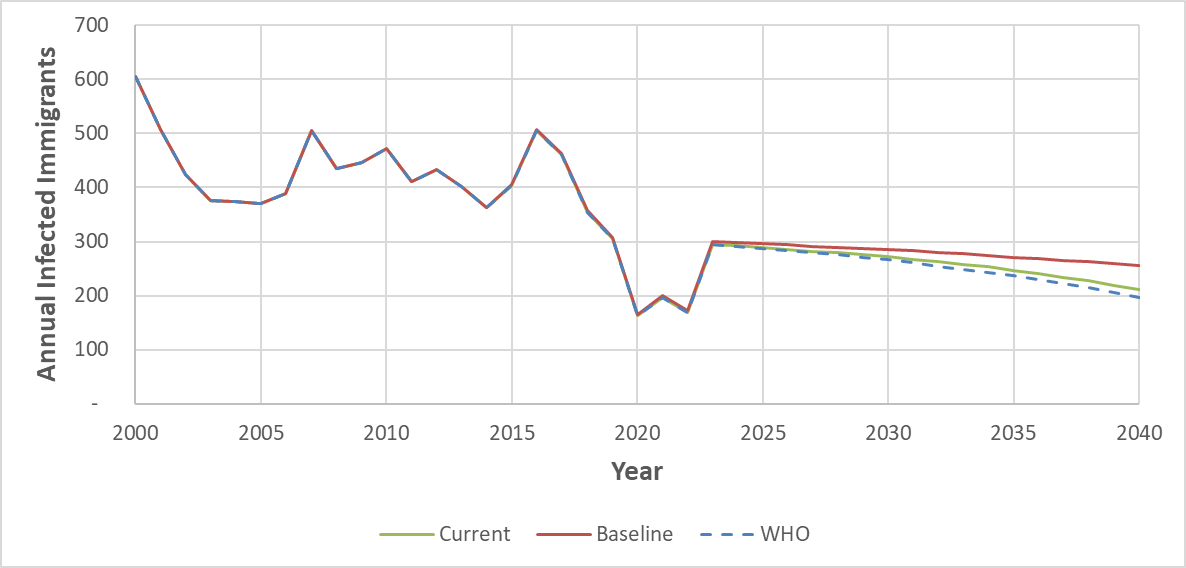
**

**References:**

Tohme RA, Andre-Alboth J, Tejada-Strop A, Shi R, Boncy J, François J, Domercant JW, Griswold M, Hyppolite E, Adrien P, Kamili S. Hepatitis B virus infection among pregnant women in Haiti: A cross-sectional serosurvey. J Clin Virol. 2016 Mar;76:66-71. doi: 10.1016/j.jcv.2016.01.012. Epub 2016 Jan 28. PMID: 26851543; PMCID: PMC5802338.

Rein DB, Lesesne SB, O'Fallon A, Weinbaum CM. Prevalence of hepatitis B surface antigen among refugees entering the United States between 2006 and 2008. Hepatology. 2010 Feb;51(2):431-4. doi: 10.1002/hep.23353. PMID: 19902482.

Ropero Álvarez AM, Pérez-Vilar S, Pacis-Tirso C, Contreras M, El Omeiri N, Ruiz-Matus C, Velandia-González M. Progress in vaccination towards hepatitis B control and elimination in the Region of the Americas. BMC Public Health. 2017 Apr 17;17(1):325. doi: 10.1186/s12889-017-4227-6. PMID: 28415981; PMCID: PMC5392937.
